# Forwards and backwards – synthesis of *Laurencia* natural products using a biomimetic and retrobiomimetic strategy incorporating structural reassignment of laurefurenynes C–F†

**DOI:** 10.1039/d0sc04120c

**Published:** 2020-10-08

**Authors:** Hau Sun Sam Chan, Amber L. Thompson, Kirsten E. Christensen, Jonathan W. Burton

**Affiliations:** Department of Chemistry, Chemistry Research Laboratory, University of Oxford Mansfield Road Oxford OX1 3TA UK jonathan.burton@chem.ox.ac.uk

## Abstract

Laurefurenynes C–F are four natural products isolated from *Laurencia* species whose structures were originally determined on the basis of extensive nuclear magnetic resonance experiments. On the basis of a proposed biogenesis, involving a tricyclic oxonium ion as a key intermediate, we have reassigned the structures of these four natural products and synthesized the four reassigned structures using a biomimetic approach demonstrating that they are the actual structures of the natural products. In addition, we have developed a synthesis of the enantiomers of the natural products laurencin and deacetyllaurencin from the enantiomer of (*E*)-laurefucin using an unusual retrobiomimetic strategy. All of these syntheses have been enabled by the use of tricyclic oxonium ions as pivotal synthetic intermediates.

## Abstract 2

The synthesis and structural reassignment of laurefurenynes C–F has been achieved, with the new structures fitting with a proposed biosynthesis. Also reported is the synthesis of *ent*-laurencin and *ent*-deacetyllaurencin via a retrobiomimetic approach.

## Graphical abstract

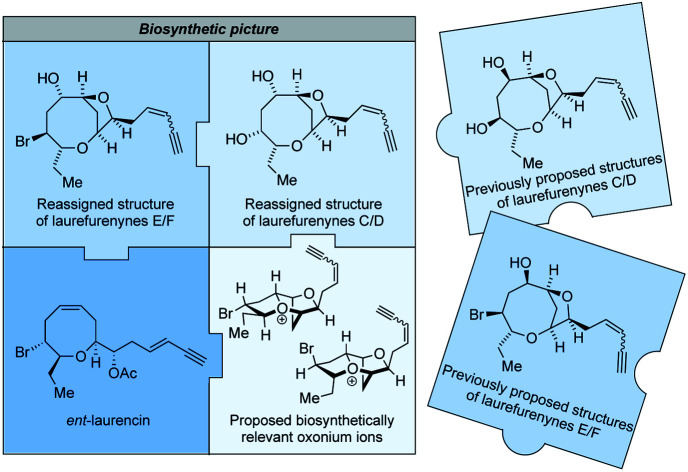

## Introduction

The C_15_-acetogenin natural products isolated from *Laurencia* species display both wide structural diversity and wide ranging biological activity which have piqued the interest of synthetic organic chemists since the initial isolation of (+)-laurencin in 1965.^1–3^ These natural products generally contain cyclic ethers, and frequently medium-ring ethers, and have served as a test-bed for the development of numerous new and efficient methodologies to allow access to complex cyclic ethers.^4–7^ The structural assignment of many of these acetogenic natural products has come primarily from high-field NMR experiments with single crystal X-ray diffraction being used where possible. The unambiguous assignment of natural products structures with compounds not suitable for single crystal X-ray analysis^8^ is a challenging task and it is inevitable that incorrect structures are reported in the literature with these structural misassignments frequently being uncovered through total synthesis.^9–11^ Within the C_15_ acetogenic natural products isolated from *Laurencia* species a number of structural misassignments have also occurred that have varied from gross structural reassignments,^12–15^ through to atom transpositions^16–19^ and stereochemical reassignment.^20–22^ Key to correcting many of these structural misassignments have been postulates regarding the biosyntheses of acetogenic *Laurencia* natural products, many of which have been proposed to proceed through complex oxonium ion intermediates.^15,23–33^ These biogenetic postulates have allowed rational prediction of the likely structures of a number of the natural products^15,21,22,26,34^ and in a number of cases the biogenetic arguments have been augmented by DFT calculations of both proton and carbon NMR chemical shifts^14,22,34^ for a range of candidate structures with ultimately the structure of the natural products being established through total synthesis.^35^ Among the acetogenic *Laurencia* natural products whose structures have been reassigned are laurefucin,^36,37^ obtusallenes V, VI and VII,^16,19,26^ elatenyne,^12–15,38^ laurendecumenyne B,^15,39,40^ aplysiallene,^41,42^ a chloroenyne from *Laurencia* majuscula^13,34,38,43^ and laurefurenynes A and B,^20–22^ additionally the structures of the ocellenynes have been tentatively reassigned on the basis of biogenetic considerations and DFT calculations of NMR chemical shifts.^24,44,45^

**Fig. 1:**
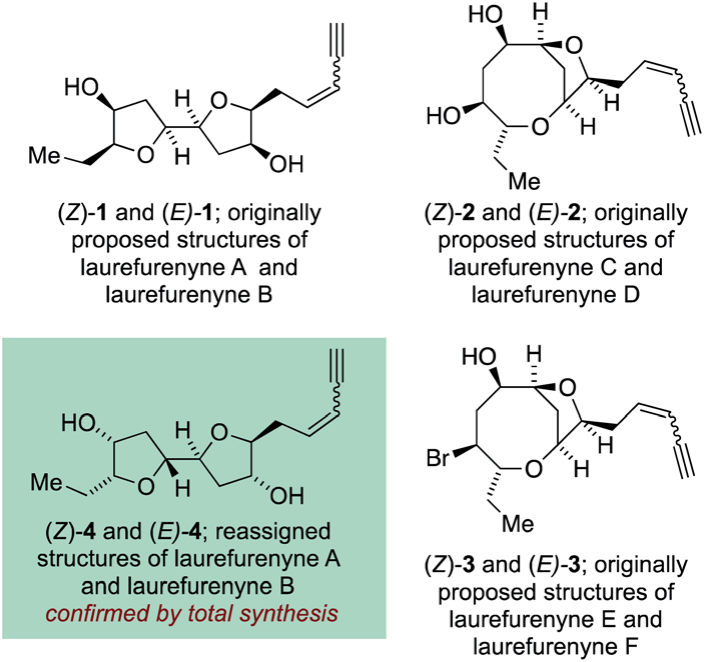
Originally proposed structures of laurefurenynes A–F along with reassigned structures of laurefurenynes A and B.

**Scheme 1:**
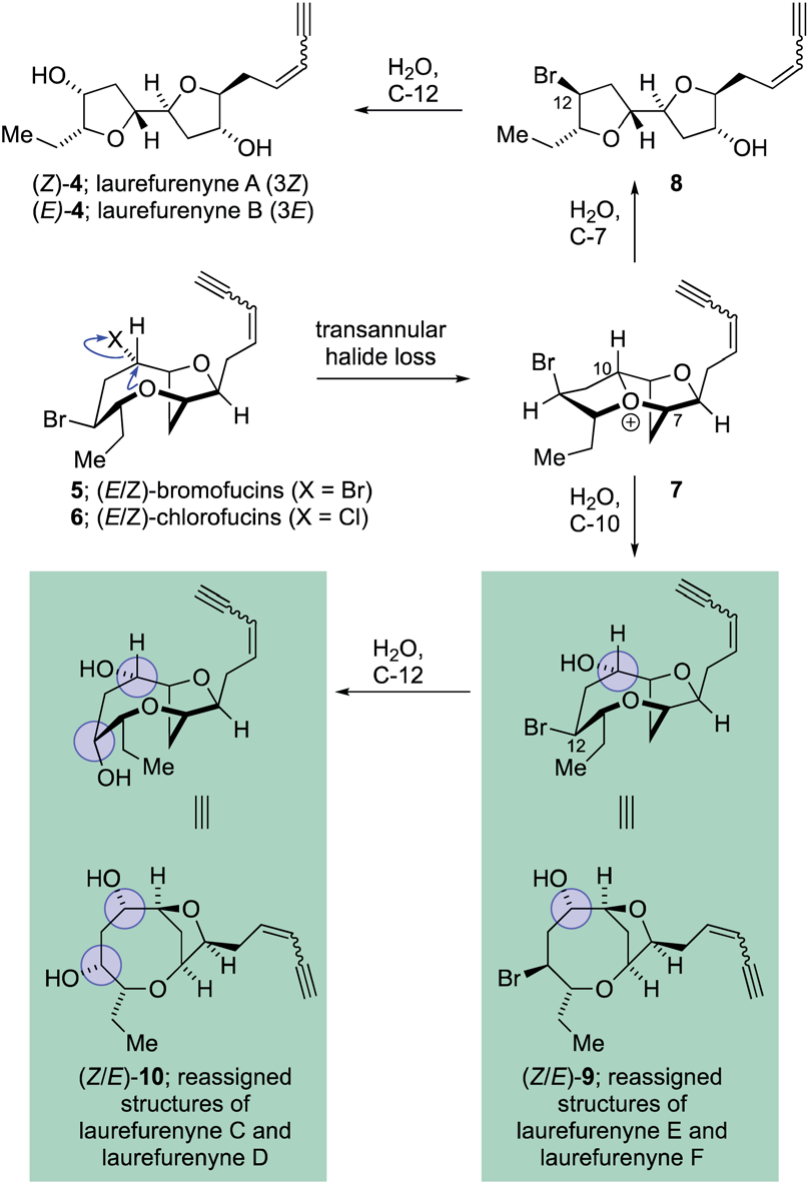
Proposed biosynthesis of the reassigned structures of laurefurenynes A–F from the halofucins.

The laurefurenynes are a series of six acetogenic natural products which were isolated and characterized by Jaspars and co-workers from a sample of *Laurencia* sp. collected in the Philippines in 1991.^20^ The structures of these six natural products ((*Z*/*E*)-**1**, (*Z*/*E*)-**2** and (*Z*/*E*)-**3**) were assigned through extensive 1D and 2D NMR experiments (Fig. 1).^46^ In 2013 the Britton group^21^ and our own group^22^ published structural reassignments of laurefurenynes A and B from (*Z*/*E*)-**1** to (*Z*/*E*)-**4** based on total synthesis and DFT calculations of NMR chemical shifts. In the original paper of Jaspars, a plausible biogenesis of the laurefurenynes (((*Z*/*E*)-**1**, (*Z*/*E*)-**2** and (*Z*/*E*)-**3**) was proposed based on epoxide and/or bromonium ion cyclizations. However, the reassigned structures of laurefurenynes A and B (*Z*/*E*)-**4** fit with a proposed biosynthesis involving the previously characterized tricyclic oxonium ions **7** ^47^ which likely arise *via* transannular displacement of bromide or chloride from the halofucins **5** and **6** (Scheme 1),^15,48,49^ followed by opening of the oxonium ions **7** at C-7 with water and then displacement of the C-12 bromine atom with water with inversion of configuration.^21,22^ If it is indeed the case that laurefurenynes A and B (*Z*/*E*)-**4** are biosynthesized from the halofucins **5** and **6** then it would be reasonable to postulate that laurefurenynes C–F are produced on the same biosynthetic pathway.^21^ Here, opening of the oxonium ions **7** with water, or a water equivalent,^26,34^ at C-10 would produce the reassigned structures of laurefurenynes E and F (*Z*/*E*)-**9** ^50^ and displacement of the bromine substituent with inversion of configuration by water or a water equivalent would yield the reassigned structures of laurefurenynes C and D (*Z*/*E*)-**10**.

Based on the above analysis, we report the first synthesis and the first biomimetic synthesis of the reassigned structures of laurefurenynes C–F (*Z*/*E*)-**10** and (*Z*/*E*)-**9**. Confirmation that the structures of the actual natural products are represented by (*Z*/*E*)-**10** and (*Z*/*E*)-**9** comes through comparative ^1^H and ^13^C nuclear magnetic resonance data for our synthetic material with that of the natural material,^20^ Mosher's ester analysis to establish the absolute configuration of a newly installed secondary alcohol,^51,52^ single crystal X-ray diffraction data giving the structure of the reassigned natural product laurefurenyne D (*E*)-**10** and the reassigned dioxabicyclo[5.2.1]decane cores **12** of laurefurenynes C–F.^53^ In combination with the reported reassigned structures of laurefurenynes A and B (*Z*/*E*)-**4** by Britton and our own group,^21,22^ reassignment of the structures of the remaining laurefurenynes (*Z*/*E*)-**9** and (*Z*/*E*)-**10** offers chemical evidence to prompt reevaluation of the initially proposed biosynthesis of these natural products.^20^ In addition, the development of methods necessary for the total synthesis of the reassigned structures of laurefurenynes C–F (*Z*/*E*)-**9** and (*Z*/*E*)-**10** enabled the total synthesis of the enantiomers of the natural products laurencin^1–3^ and deacetyllaurencin^54,55^ namely *ent*-laurencin and *ent*-deacetyllaurencin using an unusual retrobiomimetic approach.^56^

## Results and discussion

**Fig. 2:**
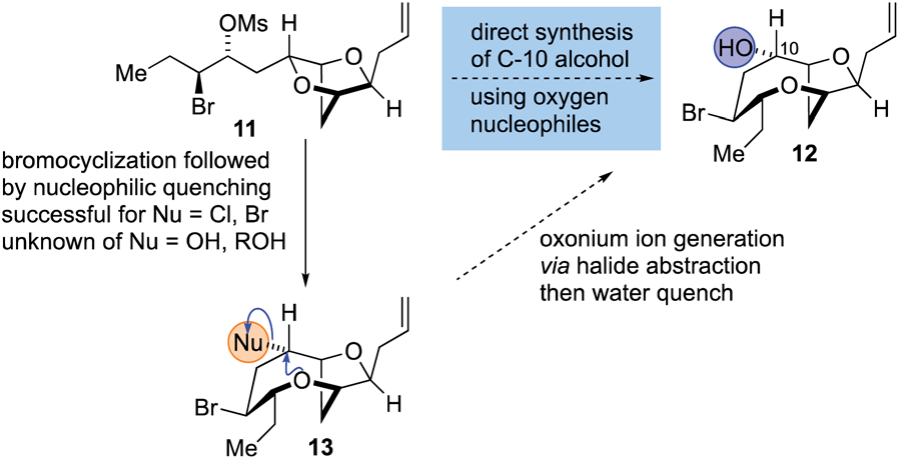
Proposed strategy for the synthesis of **12** by the direct use of oxygen nucleophiles in the bromocyclization–nucleophilic quenching reaction.

**Scheme 2:**
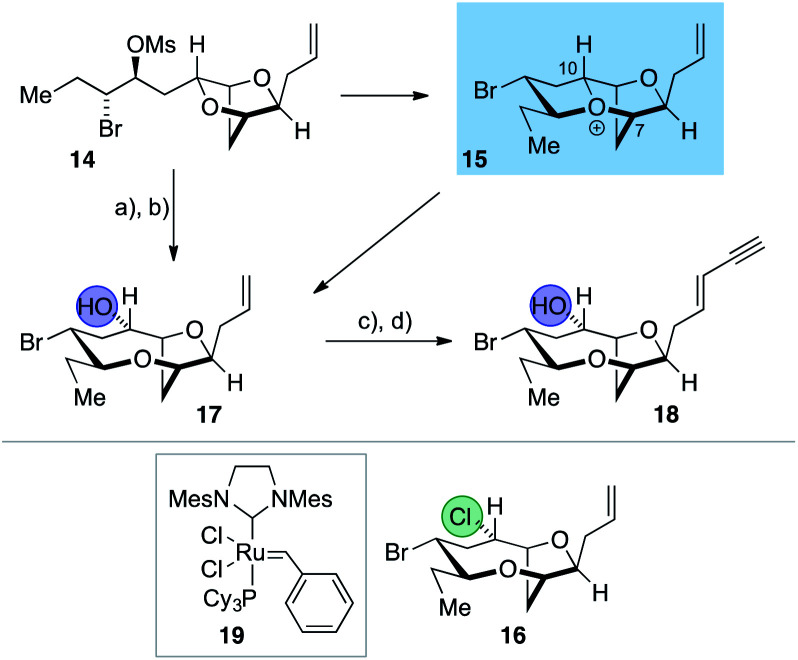
Synthesis of **17** by the direct use of oxygen nucleophile (AgOBz) and its conversion to *ent*-(*E*)-laurefucin **18**. *Reagents and conditions*: (a) AgAl(pftb)_4_·CH_2_Cl_2_, TiCl_4_, CH_2_Cl_2_, −40 °C, 2 h, then AgOBz, −78 °C, 1 h, then work-up with sat. NaHCO_3_ and excess Bu_4_N^+^I^−^; (b) K_2_CO_3_, MeOH, 74% (2 steps); (c) crotonaldehyde, cat. **19**, cat. CuI, Et_2_O, RT, overnight; (d) TMSCHN_2_, lithium diisopropylamide, −78 °C, 1 h, then 0 °C, 1 h, **18** 41% *E* : *Z* > 20 : 1 (two steps).

The synthesis of the reassigned structures of laurefurenynes C–F (*Z*/*E*)-**10** and (*Z*/*E*)-**9** began with studies concerning the expansion of the scope of our previously reported bromocyclization–nucleophilic quenching reaction of known enantiopure bromomesylates **11** ^57^ to include oxygen nucleophiles (Fig. 2). In our previous report, only chloride and bromide had been used as nucleophiles in this transformation and extra steps were required for the introduction of oxygen substituents at the desired C-10 position.^47^ The expansion of the scope of nucleophiles would allow a direct bromocyclization–oxygenation reaction of **11** to provide the dioxabicyclo[5.2.1]decane core of the reassigned structures of laurefurenynes E–F (**12**) more efficiently, obviating the need for the intermediacy of **13** (Nu = Cl, Br) as a synthetic intermediate. We conducted the initial studies on expanding the scope of the bromocyclization/nucleophilic quenching reaction with the known enantiopure bromomesylate **14** ^57^ instead of **11**, as the reaction with **14** was consistently found to give exclusive C-10 opening products with halide nucleophiles,^47^ therefore simplifying reaction analysis. Thus, exposure of the bromomesylate **14** to our previously described conditions (AgAl(pftb)_4_·CH_2_Cl_2_ (pftb = perfluoro-*t*-butoxy) and TiCl_4_) at low temperature which likely generates the oxonium ion **15**, followed by addition of saturated aqueous NaHCO_3_ solution, unexpectedly gave what appeared to be the previously reported chloride **16** as the major product by TLC analysis *versus* an authentic sample (Scheme 2).^47^ However, using excess AgOBz as the oxygen nucleophile in the place of saturated aqueous NaHCO_3_, gave the C-10 alcohol **17** in 74% overall yield after methanolysis of the crude benzoate ester; **17** has previously been reported by both Kim and Snyder.^58–60^ The formation of **17** with AgOBz rather than aqueous NaHCO_3_ solution is most likely due to the chloride anion scavenging ability of Ag^+^, rendering chloride anions unable to compete with benzoate anions for nucleophilic attack at the oxonium ion intermediate **15**.^61^ Following Kim's two step procedure^58^ compound **17** was readily transformed into *ent*-(*E*)-laurefucin **18**.^36,37,58,59,62,63^ Hence, cross-metathesis of **17** with crotonaldehyde using catalyst **19** and copper(i) iodide,^64^ followed by the Colvin–Ohira reaction using trimethylsilyldiazomethane and lithium diisopropylamide installed the (*E*)-enyne in 41% overall yield giving *ent*-(*E*)-laurefucin **18** that had identical ^1^H NMR data to a sample we had prepared previously. The matching spectroscopic data for our synthetic **17** and **18** with material prepared by Kim and Snyder confirmed that the C-10 alcohol had the configuration shown.^65^

**Scheme 3:**
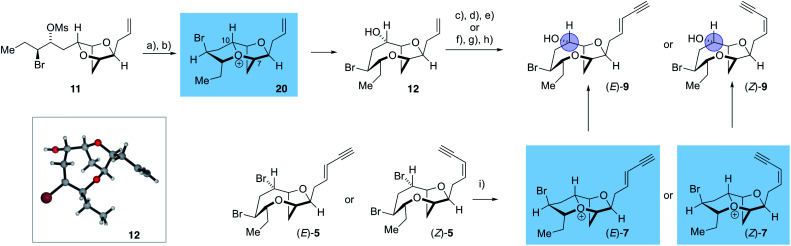
Synthesis of the reassigned structures of laurefurenynes E–F **9***via***12**. (a) TiCl_4_, AgAl(pftb)_4_·CH_2_Cl_2_, CH_2_Cl_2_, −40 °C, 2 h then add AgOBz, −78 °C, 1 h, then work-up with sat. NaHCO_3_ and excess Bu_4_NI; (b) K_2_CO_3_, MeOH, 63% (2 steps); (c) OsO_4_, NaIO_4_, 2,6-lutidine, dioxane, water; (d) ICH_2_PPh_3_I, NaHMDS, HMPA, THF, −78 °C to r.t.; (e) Me_3_SiC≡CH, Pd(PPh_3_)_4_, CuI, Et_3_N, then K_2_CO_3_, MeOH, (*Z*)-**9** 35% *Z* : *E* > 10 : 1 (four steps); (f) crotonaldehyde, cat. **19**, cat. CuI, Et_2_O, r.t., overnight; (g) TMSCHN_2_, lithium diisopropylamide, −78 °C, 1 h, then 0 °C, 1 h; (h) crude treated with K_2_CO_3_, MeOH, (*E*)-**9** 29% *E* : *Z* > 20 : 1 (three steps); (i) AgAl(pftb)_4_·CH_2_Cl_2_, CD_2_Cl_2_, −40 °C, 1.5 h, then filter at −78 °C and NMR analysis of **7**, then retrieve NMR sample, excess H_2_O followed by NaHCO_3_(s), −78 °C, 15 min then warm to RT (*Z*)-**9** 39%; (*E*)-**9** 47%.

The use of AgOBz as the oxygen nucleophile was then applied to the bromocyclization–nucleophilic quenching reaction with known enantiopure bromomesylate **11** (Scheme 3). The desired C-10 oxygenated compound **12** was obtained in 63% overall yield after methanolysis of the crude benzoate ester, without any product from C-7 oxygenation being observed. The structure of alcohol **12** was confirmed by single crystal X-ray diffraction studies^53^ (Flack *x* absolute structure parameter = −0.034(5); shown in Scheme 3).^66^ Alcohol **12** was then transformed into the reassigned structures of laurefurenyne E and F (*Z*)-**9** and (*E*)-**9** using known procedures for the stereoselective synthesis of (*Z*)- and (*E*)-enynes. Thus, oxidative cleavage of the terminal olefin in **12**,^67^ followed by Stork–Zhao olefination gave the corresponding (*Z*)-vinyl iodide,^68^ followed by Sonogashira coupling with trimethylsilylacetylene and TMS deprotection gave (*Z*)-**9**. In a related manner, the reassigned structure of laurefurenyne F (*E*)-**9** was synthesized from **12** by a cross-metathesis, Colvin–Ohira sequence.^58^ We were unable to obtain X-ray crystal structures of (*E*)- and (*Z*)-**9**, however, (*E*)- and (*Z*)-**9** were also synthesized from (*E*)- and (*Z*)-bromofucin (*E*)- and (*Z*)-**5***via* the previously characterized oxonium ions **7** ^47^ suggesting that the synthetic route from **12** to **9** had not resulted in undesired alterations of stereocenters. Hence, the oxonium ions (*Z*)- and (*E*)-**7**, readily formed from the corresponding bromofucins on treatment with a silver(i) salt followed by low temperature NMR analysis, were quenched with water to give (*Z*)- and (*E*)-**9** respectively.

The stereochemical assignment at C-10 of laurefurenynes E and F **9** was further supported by Mosher ester analysis of the C-10 secondary alcohol (blue circle) (see ESI†),^51,52^ providing further evidence that the absolute configuration of C-10 is (*S*), in keeping with the X-ray crystal structure of **12**. The spectroscopic data of (*E*)-**9** were in accordance with the data for the natural product laurefurenyne F ([*α*]_D_^25^ = +17.5 (*c* = 0.12, MeOH)lit.^20^ [*α*]_D_^25^ = +17.0 (*c* = 0.10, MeOH)) for (*Z*)-**9**, the NMR spectroscopic data are in accordance with the published data for the natural product laurefurenyne E; however, the specific rotation of natural laurefurenyne E was reported to be [*α*]_D_^25^ = +11.0 (*c* = 0.10, MeOH) whereas that of our synthetic material was [*α*]_D_^25^ = −5.9 (*c* = 0.12, MeOH) for a sample that has a *Z* : *E* ratio of > 15 : 1. Measurement of the specific rotation of a sample with a lower *Z* : *E* ratio of 7.8 : 1 gave [*α*]_D_^25^ = −4.7 (*c* = 0.17, MeOH) which is still of a lower magnitude and of opposite sign compared to that reported in the isolation paper.^20^

**Scheme 4:**
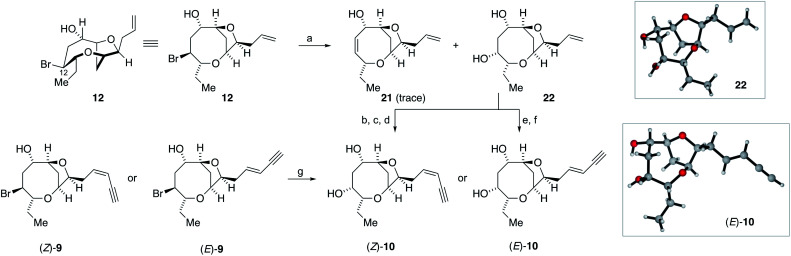
Synthesis of the reassigned structures of laurefurenynes C-D **10***via***12**. (a) CF_3_CO_2_Cs, Me_2_NCHO, 120 °C, 12 h, **22** 62%, **21**, trace; (b) OsO_4_, NaIO_4_, 2,6-lutidine, dioxane, water; (c) ICH_2_PPh_3_I, NaHMDS, HMPA, THF, −78 °C to RT; (d) Me_3_SiC≡CH, Pd(PPh_3_)_4_, CuI, Et_3_N, then K_2_CO_3_, MeOH, (*Z*)-**10** 32% *Z* : *E* > 10 : 1 (four steps); (e) CH_3_CH=CHCHO, cat. **19**, cat. CuI, Et_2_O, RT; (f) TMSCHN_2_, *n*BuLi, −78 °C, 1 h, then 0 °C, 30 min, then 1.0 M HCl (*E*)-**10** 81% *E* : *Z* > 20 : 1 (two steps); (g) CF_3_CO_2_Cs, Me_2_NCHO, 120 °C, 12 h, (*Z*)-**10** 23%, (*E*)-**10** 38%.

The successful generation of laurefurenynes E and F **9** from the bromofucins **5** provides chemical evidence to support their potential biosynthesis *via* the oxonium ions **7** as with other related natural products (Scheme 1).^47^ As noted above laurefurenynes C and D may well therefore arise biosynthetically from laurefurenynes E and F *via* an S_N_2 reaction at C-12 with an oxygen nucleophile. However, this transformation was expected to be challenging due to the neopentyl-like nature of C-12 coupled with the β-oxygen substituent which is known to retard the rates of nucleophilic substitution.^69^ Under a range of conditions reported for substitution at neopentyl-like centres with oxygen nucleophiles,^70–72^ substrate **12** was either unreactive, underwent low conversion or yielded the elimination product **21** (Scheme 4, see ESI† for details). Ultimately, using Fleet's method (cesium trifluoroacetate in DMF at 120 °C for 12 hours)^73^ gave the desired substitution product **22** in 62% yield along with a trace of elimination product **21**. Previously, Fujiwara and Murai have reported that bromination of alcohols with a β-oxygen that is part of a medium ring, can occur with inversion or retention of configuration depending on reaction conditions with retention of configuration being ascribed to neighboring group participation by the medium ring oxygen atom.^74^ We therefore needed to confirm the relative configuration of **22**. Pleasingly, **22** was isolated as a white crystalline solid which allowed single crystal X-ray diffraction analysis (absolute structure parameter of **22**: −0.01(9))^53,66^ which demonstrated that the C-12 alcohol in **22** was formed with inversion of configuration and confirmed that the relative configuration was as expected (Scheme 4). Analogous to the synthesis of the reassigned structures of laurefurenynes E and F **9**, two separate protocols were used for the synthesis of laurefurenynes C and D **10**, with the first synthesis starting from diol **22**. Thus, diol **22** was readily converted into laurefurenyne C by modified Lemieux–Johnson oxidation^67^ of the terminal alkene in **22** followed by Stork–Zhao olefination and Sonogashira coupling followed by deprotection. This provided the (*Z*)-enyne (*Z*)-**10** in 31% yield over the 4 steps with >10 : 1 *Z* : *E* selectivity. Alternatively, the (*E*)-enyne **10** was readily formed by Kim's procedure^58^ involving cross metathesis of **22** with crotonaldehyde followed by Colvin–Ohira reaction giving laurefurenyne D (*E*)-**10**. Laurefurenynes C and D **10** were also synthesized from laurefurenynes E and F **9** by displacement of the secondary bromides with cesium trifluoroacetate (Scheme 4).^73^ The NMR spectroscopic data for the reassigned structures of laurefurenynes C and D **10** was identical with that of the natural products. Furthermore, the optical rotations of laurefurenyne C (*Z*)-**10** ([*α*]_D_^25^ = + 10.8 (*c* = 0.13, MeOH), lit.^20^ [*α*]_D_^25^ = +20.0 (*c* = 0.10, MeOH)) and laurefurenyne D (*E*)-**10** ([*α*]_D_^25^ = +14.0 (*c* = 0.10, MeOH), lit.^20^ [*α*]_D_^25^ = +32.0 (*c* = 0.10, MeOH)) were of the same sign but of reduced magnitude compared to those of the isolated natural products indicating that the absolute configuration of the natural products is likely as shown in Schemes 1 and 4 in keeping with the proposed biosynthesis (Scheme 1). Synthetic laurefurenyne D (*E*)-**10** was initially isolated as a colorless oil. However, a single crystal suitable for single crystal X-ray diffraction analysis was obtained by slow diffusion of pentane into an ethyl acetate solution of (*E*)-**10**. The crystal structure of (*E*)-10 (shown in Scheme 4) was obtained with an absolute structure parameter of −0.07(9), providing further confirmation of its structure.^53^

**Scheme 5:**
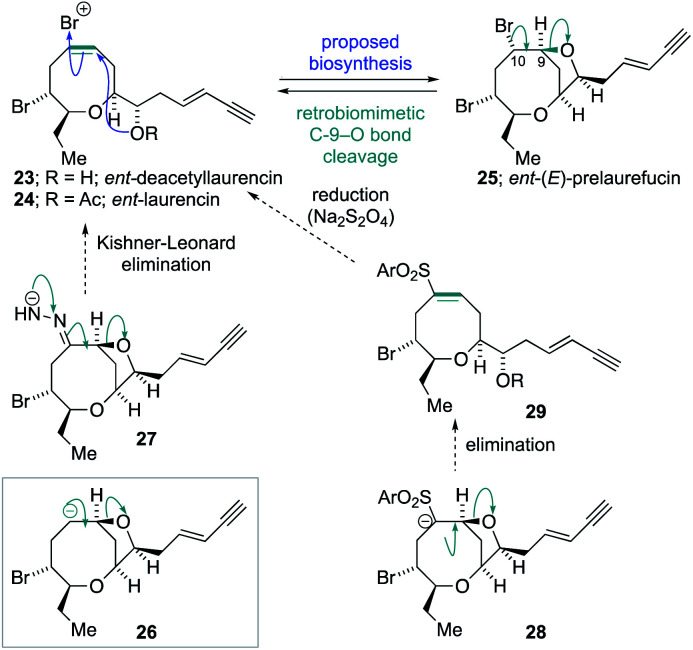
Proposed routes to *ent*-laurencin by a retrobiomimetic strategy.

**Scheme 6:**
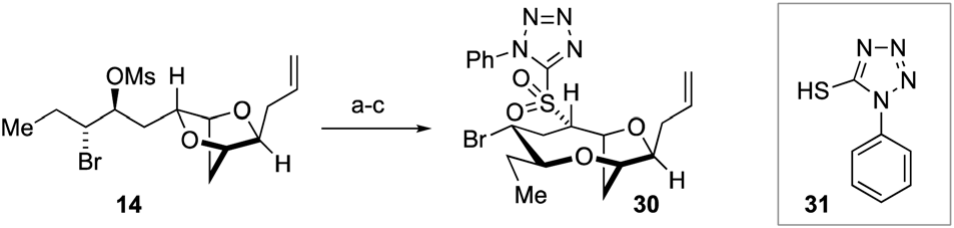
Synthesis of sulfone **30**. *Reagents and conditions*: (a) TiCl_4_, AgAl(pftb)_4_·CH_2_Cl_2_, CH_2_Cl_2,_ −40 °C, 2 h; (b) then add **31**, −78 °C, 1 h; (c) 30% aq. H_2_O_2_, cat. Mo_7_O_24_(NH_4_)_6_·4H_2_O, EtOH, 0 °C to RT overnight, 59% from **14**.

The success of the synthesis of *ent*-(*E*)-laurefucin **18**, and the reassigned structures of the laurefurenynes C–F **9** and **10**, that were enabled by the direct C-10 oxygenation of bromomesylates **11** and **14** in the bromocyclization–nucleophilic quenching reaction, prompted us to further investigate the scope of this reaction. We proposed that the enantiomer of the Δ^4^-oxocene natural products laurencin and deacetyllaurencin,^75–78^ namely *ent*-laurencin **24** and *ent*-deacetyllaurencin **23** could be synthesized *via* an unusual retrobiomimetic C_9_–O bond cleavage from *ent*-(*E*)-prelaurefucin **25** (Scheme 5). This proposed C_9_–O bond cleavage requires the formation of a formal anion at C-10 (**26**) with subsequent E1cB elimination. Reduction of *ent*-(*E*)-prelaurefucin **25** with Zn was unlikely to be selective for formation of *ent*-deacetyllaurencin given the previous precedent in a related system^37,79^ and we therefore elected to investigate two elimination reactions for the formation of **23** and **24**, namely a Kishner–Leonard elimination (**27**)^80,81^ and elimination to form a vinyl sulfone (**28** to **29**) which could undergo subsequent selective reduction with the mild reducing agent sodium dithionite.^82^ In order to put this plan into practice we needed to further expand the scope of the bromocyclization–nucleophilic quenching reaction to include sulfur nucleophiles for the synthesis of sulfones. The requisite sulfone **30** was readily prepared from enantiopure bromomesylate **14**. Oxonium ion formation from **14** was achieved as before followed by the addition of 1-phenyl-1*H*-tetrazole-5-thiol **31** as the sulfur nucleophile to give corresponding C-10 sulfide in 76% yield.^83,84^ The sulfide was then oxidized to the desired sulfone **30** in 77% yield by cat. Mo_7_O_24_(NH_4_)_6_·4H_2_O and 30% aqueous H_2_O_2_ in 77% yield (Scheme 6).

**Scheme 7:**
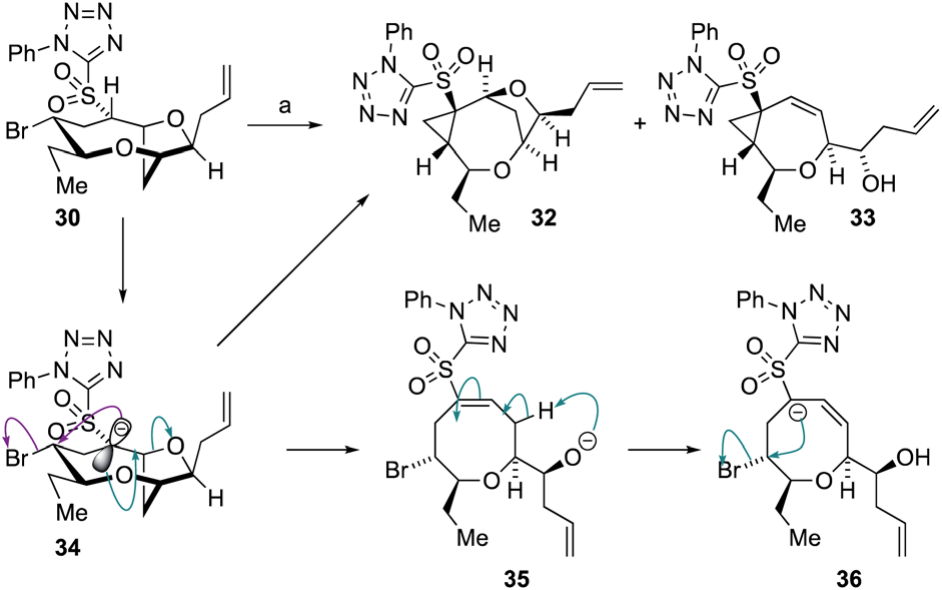
Formation of cyclopropanes **32** and **33** from **30**, and a proposed mechanism for product generation. *Reagents and conditions*: (a) 1.1 eq. NaHMDS, THF, −78 °C to RT, 15 min **32** : **33** = 1 : 2.2 90%.

Treatment of **30** with 1.1 equivalents of strong base (NaHMDS) at −78 °C resulted in no conversion of starting material but on warming to ambient temperature, complete conversion of the sulfone was observed giving the two cyclopropane-containing products **32** and **33** in a 1 : 2.2 ratio in 90% combined yield (Scheme 7). The structures of the sulfones were assigned by extensive NMR experiments including ^1^H–^1^H NOE experiments (see ESI†). A plausible mechanism for the formation of **32** and **33** is shown below. Thus, deprotonation the acidic proton adjacent to the sulfone in **30** would give the anion **34** from which direct cyclopropanation can occur giving **32** or E1cB elimination can occur giving alkoxide **35**. Alkoxide **35** can then undergo intramolecular proton transfer to provide the α-sulfonyl anion **36** from which cyclopropanation can readily occur giving **33**. The generation of product **33** indicated that the desired E1cB fragmentation was possible although the anion stabilizing properties of the sulfone promoted further reactions.

**Scheme 8:**
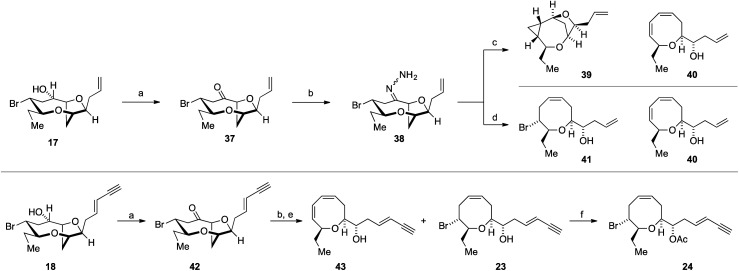
Kishner–Leonard fragmentation for Δ^4^-oxocene synthesis and the synthesis of *ent*-deacetyllaurencin **23** and *ent*-laurencin **24**. *Reagents and conditions*: (a) Dess–Martin periodinane, CH_2_Cl_2_, RT, 1 h, **37** 85%; **42** quant.; (b) N_2_H_4_·H_2_O, EtOH, RT 2 h; (c) 3.5 eq. NaH, DMSO, then add crude **38** RT, 60% 4.5 : 1 mixture of **39** and **40** (from **37**); (d) 1.1 eq. NaHMDS, THF, −78 °C to RT, **41** 42% and **40** trace (two steps); (e) 2.0 eq. NaHMDS, THF, −78 °C to RT, **23** 32% and **43** 11% (two steps); (f) Ac_2_O, DMAP, CH_2_Cl_2_, r.t., 1 h, 91%.

Our attention therefore turned to investigate the Kishner–Leonard elimination from the C-10 hydrazone **38** (Scheme 8). Oxidation of the previously synthesized alcohol **17** using Dess–Martin periodinane gave ketone **37**, which on treatment with hydrazine hydrate gave the requisite hydrazone **38** (not isolated). Following a literature procedure,^85^ treatment of the crude hydrazone **38** with dimsyl sodium on a small scale (0.7 mmol NaH – caution – sodium hydride in DMSO is a known explosion hazard)^86^ gave an inseparable mixture of the cyclopropane **39**, as the major product, along with the diene **40** (**39** : **40**, 4.5 : 1); the structures of **39** and **40** were assigned by extensive ^1^H and ^13^C NMR experiments (Scheme 8). Both the cyclopropane **39** and the diene **40** most likely arise from the hydrazone anion corresponding to **27** with formation of the diene requiring a further elimination of HBr. We were disappointed that the cyclopropane **39** was the major product of this reaction. However, when the crude hydrazone **38** was treated with 1.1 equivalents of NaHMDS in THF at −78 °C with gradual warming to room temperature, during which gas evolution was observed, the desired Δ^4^-oxocene **41** was isolated along with a trace of the 1,3-diene **40** as a side product (42% combined yield over 2 steps). This procedure was then applied to the natural product *ent*-(*E*)-laurefucin **18** for the synthesis of *ent*-laurencin **24**. Thus, oxidation of alcohol **18** gave the ketone **42** from which the corresponding hydrazone was prepared. In order to effect the Kishner–Leonard elimination on the hydrazone derived from ketone **42** it was necessary to increase in the equivalents of NaHMDS from 1.1 to 2.0 equivalents presumably due to the deprotonation of the acidic acetylene C–H. Using this procedure gave *ent*-deacetyllaurencin **23** and diene **43** in a 3 : 1 ratio in 43% combined yield over 2 steps. The natural product *ent*-laurencin **24** was obtained by acetylation of *ent*-deacetyllaurencin **23** in 93% yield. The spectroscopic data of **23** and **24** are in accordance with published data^2,55^ (synthetic *ent*-laurencin: [*α*]_D_^25^ = −61.0 (*c* = 0.10, CHCl_3_), lit.^2^ natural (+)-laurencin [*α*]_D_^23^ = +70.2 (*c* = 1.0, CHCl_3_); synthetic *ent*-deacetyllaurencin [*α*]_D_^25^ = −34.7 (*c* = 0.05, CHCl_3_), lit.^55^ natural deacetyllaurencin [*α*]_D_^17^ = +46.1 (*c* = 1.15, CHCl_3_)).

## Conclusions

In conclusion, we have reported structural reassignment of laurefurenynes C–F **9–10** confirmed through the first synthesis and the first biomimetic synthesis of the natural products based on their proposed biosynthesis. Confirmation of the reassigned natural product structures came from extensive spectroscopic and crystallographic evidence. The structural reassignment and biomimetic generation of the laurefurenynes enables them to be considered in line with a series of structurally analogous *Laurencia* natural products, which further lend weight to their proposed biosynthesis from the bromofucins *via* complex oxonium ions such as **7**. In addition, the expansion of the scope of nucleophiles compatible with the bromocyclization–nucleophilic quenching reactions enabled the preparation of a series of substrates for the investigations that led to the total synthesis of *ent*-deacetyllaurencin **23** and *ent*-laurencin **24** through a retrobiomimetic C_9_–O cleavage, which was ultimately achieved using the Kishner–Leonard elimination reaction. The use of a broad two directional biomimetic and retrobiomimetic strategy has facilitated the synthesis of six natural products. Further development of oxonium ion chemistry in general with applications in natural product synthesis are ongoing and will be reported in due course.

## Conflicts of interest

There are no conflicts to declare.

## Supplementary Material

SC-011-D0SC04120C-s001

SC-011-D0SC04120C-s002
