# Supplementary material for: Forwards and backwards – synthesis of Laurencia natural products using a biomimetic and retrobiomimetic strategy incorporating structural reassignment of laurefurenynes C–F
Source: Chem Sci. 2020 Oct 8;11(42):11592–600. doi: 10.1039/d0sc04120c (PMC8162873; doi:10.1039/d0sc04120c)
Supplement: SC-011-D0SC04120C-s001 [file SC-011-D0SC04120C-s001.pdf]

Electronic Supplementary Material (ESI) for Chemical Science.
This journal is © The Royal Society of Chemistry 2020

Supplementary Information

Forwards and backwards – synthesis of *Laurencia* natural products using a biomimetic and retrobiomimetic strategy incorporating structural reassignment of laurefurenynes C-F.

Hau Sun Sam Chan, Amber J. Thompson, Kirsten E. Christensen and Jonathan W. Burton

Department of Chemistry, Chemistry Research Laboratory,
University of Oxford,
Mansfield Road, Oxford OX1 3TA. UK
E-mail: jonathan.burton@chem.ox.ac.uk

Contents

1) General experimental 2

2) Note on previously prepared compounds 2

3) Synthesis and characterization of novel compounds and natural products 2

4) Structure determination – Mosher ester analysis and assignment of relative configurations 27

5) Comparative NMR data for natural products 31

6) Comparative specific rotation data for natural products 37

7) NMR spectra 40

8) References 66

1) General experimental

^{1}H and ^{13}C NMR spectra were recorded on a Bruker AVIII 700 (700/176 MHz), AVII 500 (500/125 MHz) and Bruker AVIII HD 500 (500/125 MHz) spectrometer. Proton and carbon chemical shifts are quoted in ppm and referenced to residual protonated solvent. Resonances are described as s (singlet), d (doublet), t (triplet), q (quartet), m (multiplet), br (broad), dd (double doublet) and so on. Coupling constants (*J*) are given in Hz and are rounded to the nearest 0.1 Hz. Low resolution mass spectra were recorded on a Fisons Platform spectrometer (ES). High resolution mass spectra were recorded by the mass spectrometry staff at the Chemistry Research Laboratory, University of Oxford, using a Bruker Daltronics microTOF spectrometer (ES) or a Micromass GCT (FI). *m/z* values are reported in Daltons. High resolution values are calculated to four decimal places from the molecular formula, all found values being within a tolerance of 5.0 ppm. Infrared spectra were recorded on a Bruker Tensor 27 Fourier Transform spectrometer using diamond ATR. Absorption maxima (ν_{max}) are described as strong, medium, weak and broad and are quoted in wavenumbers (cm^{-1}). Optical rotations were measured using a Perkin-Elmer 241 polarimeter in a cell of 1.0 dm path length (l). TLC was performed on Merck DC-Alufolien 60 F254 0.2 mm precoated plates and visualised using an acidic vanillin or basic potassium permanganate dip. Retention factors (R_f) are reported with the solvent system used in parentheses. Flash column chromatography was performed on Merck 60 silica (particle size 40– 63 µm, pore diameter 60 Å) and the solvent system used is recorded in parentheses. All non-aqueous reactions were carried out in flame-dried glassware under an inert atmosphere of argon or nitrogen and employing standard techniques for handling air-sensitive materials. Solvents and commercially available reagents were dried and purified before use, as appropriate. Compounds were named using ChemDraw and the assignments of NMR spectra were done on MestReNova. The structures of the compounds were assigned based on analysis of ^{1}H NMR spectra, ^{13}C NMR spectra, ^{1}H-^{1}H COSY (Correlation Spectroscopy) spectra, ^{1}H-^{13}C HSQC (Heteronuclear Single Quantum Correlation) spectra, ^{1}H-^{13}C HMBC (Heteronuclear Multiple Bond Correlation) spectra and ^{1}H-^{1}H NOESY (Nuclear Overhauser Effect Spectroscopy) spectra, unless otherwise specified. For compounds that gave single crystals that were suitable for single crystal X-ray diffraction, their structural assignments were further supported by their corresponding X-ray crystal structures.

2) Note on previously prepared compounds

The compounds **11**, **14**, **16**, **18**, (*E*/*Z*)-**5** and (*E*/*Z*)-**7** were prepared previously and their synthesis and characterization could be found in the referenced publication.[1]

3) Synthesis and characterization of novel compounds and natural products

(1*S*,3*S*,4*R*,6*S*,7*S*,9*S*)-9-Allyl-4-bromo-3-ethyl-2,8-dioxabicyclo[5.2.1]decan-6-ol 17

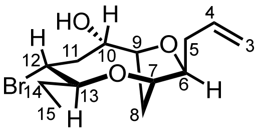

The compound **14** (55.0 mg, 0.147 mmol) was dissolved in CH_2Cl_2 (12.0 mL) and the reaction mixture was cooled to -40 °C. A solution of $TiCl_4$ in CH_2Cl_2 (0.5 M, 0.58 mL, 0.288 mmol) was then

added, quickly followed by AgAl(pftb)$_4$·CH_2Cl_2 (501.0 mg, 0.432 mmol) in CH_2Cl_2 (2.0 mL) and stirred at -40 °C for 2 hours. The reaction mixture was then cooled to -78 °C, AgOBz (315.0 mg, 1.44 mmol) was added and stirred at this temperature for 1 hour. TLC analysis at this stage indicated complete consumption of starting material. The reaction mixture was quenched with sat. aq. $NaHCO_3$ and excess TBAI. The aqueous layer was extracted with CH_2Cl_2, and all the organic layers were combined and dried with $MgSO_4$. The crude was concentrated and then dissolved in minimal volume of 50% EA/Pet. Ether 40-60 followed by filtration through a plug of silica gel. The filtrate was concentrated and MeOH (5.0 mL) and K_2CO_3 (203.0 mg, 1.47 mmol) were added and then stirred for 2 hours. TLC analysis at this stage indicated complete consumption of starting material. The reaction mixture was diluted with water and extracted with ethyl acetate. The organic layers were combined and dried with $MgSO_4$. The crude was concentrated and underwent purification by flash column chromatography (50% EA/Pet. Ether 40-60) to give the compound **17** as a colourless oil (33.4 mg, 0.109 mmol, 74%). R_f = 0.31 (50% EA/Pet. Ether 40-60). ^{1}H NMR (500 MHz, $CDCl_3$) δ 5.82 (ddt, *J* = 17.2, 10.2, 7.0 Hz, 1H, **H4**), 5.14 (dq, *J* = 17.2, 1.6 Hz, 1H, **H3'**), 5.06 (ddt, *J* = 10.2, 2.2, 1.1 Hz, 1H, **H3**), 4.18 (dd, *J* = 8.0, 3.8 Hz, 1H, **H9**), 4.03 (td, *J* = 2.4, 1.1 Hz, 1H, **H7**), 3.96 (dt, *J* = 9.0, 4.1 Hz, 1H, **H10**), 3.87 – 3.84 (m, 1H, **H12**), 3.84 – 3.79 (m, 1H, **H6**), 3.50 (td, *J* = 9.4, 2.6 Hz, 1H, **H13**), 2.52 (d, *J* = 14.4 Hz, 1H, **H8'**), 2.48 – 2.34 (m, 4H, O**H**, **H5**, **H11'**), 2.27 (dt, *J* = 15.1, 9.8 Hz, 1H, **H11**), 2.09 (dqd, *J* = 14.7, 7.4, 2.6 Hz, 1H, **H14'**), 1.89 (ddd, *J* = 14.3, 8.0, 2.5 Hz, 1H, **H8**), 1.48 (ddq, *J* = 14.3, 9.0, 7.3 Hz, 1H, **H14**), 0.95 (t, *J* = 7.3 Hz, 3H, **H15**). ^{13}C NMR (126 MHz, $CDCl_3$) δ 134.7 (**C4**), 117.4 (**C3**), 83.7 (**C9**), 83.4 (**C6**), 80.4 (**C13**), 77.9 (**C10**), 76.8 (**C7**), 53.1 (**C12**), 42.2 (**C11**), 34.3 (**C5**), 33.3 (**C8**), 28.3 (**C14**), 9.8 (**C15**). IR (ν_{max} cm^{-1}): 3394 (O–H stretching, broad), 2961-2918 (aliphatic sp^3 C–H stretchings, broad), 1642 (C=C stretching, medium), 1100 (ether C–O–C antisymmetric stretching, strong). LRMS (ESI) [M+Na]$^+$: *m/z* 327.1 and 329.1. HRMS (ESI) [M+Na]$^+$: calculated for *m/z* 327.05663 and 329.05458, found *m/z* 327.05666 and 329.05462 ($C_{13}H_{21}O_3{}^{79}BrNa$ and $C_{13}H_{21}O_3{}^{81}BrNa$). $[\alpha]_D^{25} = +46.7$ (c=0.60, $CHCl_3$). Literature value: $[\alpha]_D^{25} = -46.5$ (c=0.37, $CHCl_3$)[2]. Spectroscopic data are in accordance with literature data.[2,3]

(1*S*,3*S*,4*R*,6*S*,7*S*,9*S*)-4-Bromo-3-ethyl-9-((*E*)-pent-2-en-4-yn-1-yl)-2,8-dioxabicyclo[5.2.1] decan-6-ol, *ent*-(*E*)-laurefucin 18

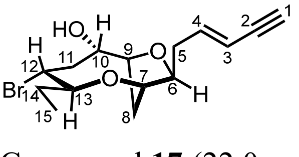

Compound **17** (22.0 mg, 0.072 mmol) was dissolved in dry Et_2O (3.6 mL) and degassed by purging with an Argon. Crotonaldehyde (30.0 µL, 0.360 mmol) was added followed by Grubbs' 2nd Generation catalyst (3.1 mg, 0.004 mmol) and CuI (1.0 mg, 0.004 mmol). The reaction mixture was stirred overnight at r.t. TLC analysis at this stage indicated complete consumption of starting material. All volatiles were removed, and the crude was purified by flash column chromatography (70% EA/ Pet. Ether 40-60) to afford the intermediate enal (16.3 mg, 0.049 mmol, 68%) which was immediately used for the next step. A stock solution of LDA (0.5 M in THF, 1.0 mL, 0.5 mmol) was cooled to −78 °C and $TMSCHN_2$ (2.0 M in Et_2O, 0.25 mL, 0.5 mmol) was added, and stirred for 30 minutes at −78

°C. The intermediate enal (16.3 mg, 0.049 mmol) was dissolved in dry THF (2.0 mL) and cooled to −78 °C. A portion of the TMSCLiN$_2$ solution (0.4 mL) was extracted quickly and added to the solution of the intermediate enal at −78 °C and left to stir at the same temperature for 1 hour. The reaction mixture was warmed to 0 °C and further stirred for an hour. TLC analysis at this stage indicated complete consumption of starting material. The reaction was quenched with 1M HCl, extracted with ethyl acetate, and the combined organic layer was washed with sat. NaHCO$_3$ solution. The washed organic layer was dried with anhydrous MgSO$_4$, filtered and concentrated, and followed by purification by flash column chromatography (30% EA/ Pet. Ether 40-60 to 50% EA/ Pet. Ether 40-60) to afford the desired compound **18** as a white powder. (10.0 mg, 0.030 mmol, 41% over 2 steps). R_f = 0.50 (50% EA/Pet. Ether 40-60). ^{1}H NMR (500 MHz, C_6D_6) δ 6.23 (dt, *J* = 15.9, 7.3 Hz, 1H, **H4**), 5.53 (dq, *J* = 16.0, 1.7 Hz, 1H, **H3**), 4.01 (dd, *J* = 8.0, 3.6 Hz, 1H, **H9**), 3.62 (dt, *J* = 8.1, 3.6 Hz, 1H, **H10**), 3.40 (td, *J* = 10.4, 4.7 Hz, 1H, **H12**), 3.32 (td, *J* = 7.2, 2.4 Hz, 1H, **H6**), 3.21 (q, *J* = 2.0 Hz, 1H, **H7**), 3.03 (ddd, *J* = 11.3, 9.1, 2.5 Hz, 1H, **H13**), 2.56 (d, *J* = 2.3 Hz, 1H, **H1**), 2.35 (dtd, *J* = 13.9, 6.9, 1.7 Hz, 1H, **H5'**), 2.26 (dtd, *J* = 14.0, 7.7, 1.5 Hz, 1H, **H5**), 2.13 (ddd, *J* = 14.9, 4.7, 1.2 Hz, 1H, **H11'**), 2.02 – 1.91 (m, 2H, **H11**, **H14'**), 1.82 (d, *J* = 14.3 Hz, 1H, **H8'**), 1.74 (d, *J* = 4.0 Hz, 1H, O**H**), 1.25 (ddq, *J* = 14.3, 8.9, 7.1 Hz, 1H, **H14**), 1.09 (ddd, *J* = 14.3, 8.0, 2.5 Hz, 1H, **H8**), 0.76 (t, *J* = 7.4 Hz, 3H, **H15**). Spectroscopic data are in accordance with literature data and data from a previous sample prepared by our group. [1,2,4] Our synthetic material also provided single crystals that were suitable for single crystal X-ray diffraction studies which further support our structural assignment (CCDC 2005093).

(1*S*,3*R*,4*S*,6*S*,7*S*,9*S*)-9-Allyl-4-bromo-3-ethyl-2,8-dioxabicyclo[5.2.1]decan-6-ol 12

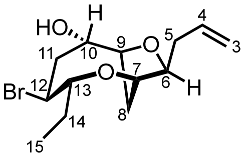

Compound **11** (25.0 mg, 65.0 μmol) was dissolved in dry CH_2Cl_2 (6.5 mL) and the reaction mixture was cooled to -40 °C. $TiCl_4$ (0.26 mL, 0.5 M in CH_2Cl_2, 195.0 μmol) was added followed by a solution of AgAl(pftb)$_4$·CH_2Cl_2 (226.0 mg, 130.0 μmol, in 1.0 mL CH_2Cl_2) immediately. The reaction mixture was stirred at -40˚C for 2 hours, then cooled to -78 °C. AgOBz (149.0 mg, 0.65 mmol) was added to the reaction mixture and stirring was continued for 1 hour. The reaction mixture was then quenched with sat. aq. NaHCO$_3$, warmed to r.t. and excess TBAI was added. The quenched reaction mixture was then extracted with CH_2Cl_2, the organic layers were combined and dried with MgSO$_4$. The crude was concentrated and then dissolved in minimal volume of 50% EA/Pet. Ether 40-60 followed by filtration through a plug of silica gel. The filtrate was concentrated then dissolved in MeOH (2.2 mL), K_2CO_3 (90.0 mg, 0.65 mmol) was added and then stirred for 2 hours. TLC analysis at this stage indicated complete consumption of starting material. The reaction mixture was diluted with water and extracted with ethyl acetate. The organic layers were combined and dried with MgSO$_4$. The crude was concentrated and underwent purification by flash column chromatography (40% EA/ Pet. Ether 40-60) to give the compound **12** as a colourless oil, which sometimes would solidify upon standing

(12.6 mg, 41.2 μmol, 63%). R_f = 0.40 (40% EA/ Pet.Ether 40-60). ^{1}H NMR (500 MHz, $CDCl_3$) δ 5.81 (ddt, *J* = 17.3, 10.2, 7.1 Hz, 1H, **H4**), 5.15 (dq, *J* = 17.2, 1.5 Hz, 1H, **H3'**), 5.06 (ddt, *J* = 10.2, 2.2, 1.1 Hz, 1H, **H3**), 4.59 (t, *J* = 9.5 Hz, 1H, **H12**), 4.14 (ddd, *J* = 9.7, 3.2, 2.0 Hz, 1H, **H9**), 4.02 – 3.99 (m, 1H, **H10**), 3.98 (t, *J* = 2.5 Hz, 1H, **H7**), 3.89 (ddd, *J* = 11.8, 9.5, 2.6 Hz, 1H, **H13**), 3.72 (td, *J* = 7.1, 1.9 Hz, 1H, **H6**), 3.05 (ddd, *J* = 15.2, 9.7, 1.8 Hz, 1H, **H11'**), 2.54 (dt, *J* = 14.1, 7.1 Hz, 1H, **H5'**), 2.44 (dt, *J* = 13.9, 7.1 Hz, 1H, **H5**), 2.31 (dd, *J* = 15.2, 5.2 Hz, 1H, **H11**), 2.27 (d, *J* = 15.3 Hz, 1H, **H8'**), 2.02 – 1.94 (m, 2H, **H8**, **H14'**), 1.70 (ddq, *J* = 14.2, 11.3, 7.1 Hz, 1H, **H14**), 1.04 (t, *J* = 7.3 Hz, 3H, **H15**). ^{13}C NMR (126 MHz, $CDCl_3$) δ 134.9 (**C4**), 117.3 (**C3**), 84.1 (**C6**), 83.4 (**C13**), 78.8 (**C9**), 70.6 (**C10**), 70.0 (**C7**), 52.0 (**C12**), 38.9 (**C11**), 33.5 (**C5**), 33.2 (**C8**), 23.2 (**C14**), 11.9 (**C15**). IR (ν_{max} cm^{-1}): 3409 (O–H stretching, broad), 3077 (alkene sp^2 =C–H stretching, weak), 2964-2931 (aliphatic sp^3 C–H stretchings, broad), 1642 (C=C stretching, medium), 1041 (ether C–O–C antisymmetric stretching, strong), 656 (C–Br stretching, medium). LRMS (ESI) [M+Na]$^+$: *m/z* 327.1 and 329.1. HRMS (ESI) [M+Na]$^+$: calculated for *m/z* 327.05663 and 329.05458, found *m/z* 327.05671 and 329.05469. ($C_{13}H_{21}O_3{}^{79}BrNa$ and $C_{13}H_{21}O_3{}^{81}BrNa$). $[\alpha]_D^{25} = +3.5$ (c=0.20, $CHCl_3$) . The structure of compound **12** was further confirmed by X-ray crystallography (CCDC 2005092).

(1*S*,3*R*,4*S*,6*S*,7*S*,9*S*)-4-Bromo-3-ethyl-9-((*E*)-pent-2-en-4-yn-1-yl)-2,8-dioxabicyclo [5.2.1]decan-6-ol, laurefurenyne F (*E*)-9

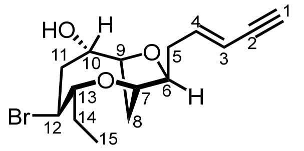

Synthesis from recycled NMR sample:

Compound (*E*)-**5** (5.0 mg, 13.0 μmol) was dissolved in dry CD_2Cl_2 (0.3 mL) and the reaction mixture was cooled to -40 °C. AgAl(pftb)$_4$·CH_2Cl_2 (16.2 mg, 14.0 μmol) in dry CD_2Cl_2 (0.3 mL) was then added at -40 °C. The reaction mixture was stirring was continued for 15 minutes at -40 °C in which precipitation of a brown substance was observed. The reaction mixture was then continued to stir at this temperature for 1 hour. An optional TLC analysis could be carried out at this point to confirm if the starting material is completely consumed. The reaction mixture was then cooled to -78˚C and equilibrated for 30 minutes. The reaction mixture was then filtered at -78˚C into a flame dried and argon filled Young's NMR tube for subsequent NMR analyses. The colour of the filtrate could range from pale yellow to pale pink. After the analysis, the NMR sample was warmed to ca. -20 °C -0 °C, ejected from the spectrometer and incubated at -78 °C in a dry ice-acetone bath, and an excess of water was added. The sample was quickly removed from the bath, shaken vigorously and a pinch of $NaHCO_3$ was added. The sample was then returned to the bath and incubated for 15 minutes before gradually warming to room temperature. The reaction mixture was extracted with CH_2Cl_2, the organic layer was separated, dried with $MgSO_4$ and concentrated. The crude then underwent purification by flash column chromatography (10% EA/CH_2Cl_2 then 20% EA/CH_2Cl_2) to give the desired compound (*E*)-**9** as a colourless oil (2.0 mg, 6.2 μmol, 47%).

Synthesis without NMR analysis:

Compound (E)-**5** (3.8 mg, 9.7 µmol) was dissolved in dry CH_2Cl_2 (1.5 mL) and the reaction mixture was cooled to -40 °C. $AgAl(pftb)_4{\cdot}CH_2Cl_2$ (12.4 mg, 11.0 µmol) in dry CH_2Cl_2 (0.5 mL) was then added at -40 °C. A precipitation of a brown substance was observed. The reaction was stirred at -40 °C for 1 hour, then cooled to -78 °C and excess H_2O was added followed by a pinch of $NaHCO_3$. The reaction mixture was then warmed to room temperature and excess tetrabutylammonium iodide was added to the reaction mixture, in which the colour of the organic layer changed from pale purple to pale yellow. The reaction mixture was extracted with CH_2Cl_2, the organic layer was separated, dried with $MgSO_4$ and concentrated. The crude then underwent purification by flash column chromatography twice (50% EA/Pet.Ether 40-60 to remove tetrabutylammonium salts) then (20% EA/CH_2Cl_2 to separate the desired product from other minor impurities) to give the desired compound (E)-**9** as a colourless oil (2.2 mg, 6.7 µmol, 69%).

Synthesis from 12:

According to the procedures of Voigtritter[5]: The compound **12** (18.0 mg, 59.0 µmol) was dissolved in Et_2O (3.0 mL) and degassed with Argon. Crotonaldehyde (24.0 µL, 0.30 mmol) was added. Grubb's 2nd generation catalyst (2.5 mg, 3.0 µmol) was added, followed by CuI (0.8 mg, 4.4 µmol) and then stirred at room temperature overnight. TLC analysis at this stage confirmed the complete consumption of starting materials. All volatiles were then removed under reduced pressure, and the residue then underwent purification by flash column chromatography (50% EA/ Pet. Ether 40-60, 70% EA/ Pet. Ether 40-60 then 100% EA) to give the enal (14.0 mg, 41.9 µmol, 71%) which was carried forward immediately to the next step.

According to the procedures of Kim[2]: A solution of $TMSCHN_2$ in Et_2O (2.0M, 0.21 mL, 0.42 mmol) was added to a solution of LDA (0.5M, 0.84 mL, 0.42 mmol) at -78 °C dropwise. The enal (14.0 mg, 41.9 µmol) was then dissolved in THF (1.0 mL) and added to the $TMSCLiN_2$ solution at -78 °C dropwise. The reaction mixture was stirred at -78 °C for 1 hour then 0 °C for 1 hour. TLC analysis at this stage confirmed the complete consumption of starting materials. The reaction mixture was quenched with sat. aq. NH_4Cl and extracted with Et_2O. The organic layers were combined, dried with anhydrous $MgSO_4$ and concentrated, diluted with 10% EA/Pet. Ether 40-60 and then filtered through a short plug of silica gel. The filtrate was concentrated and dissolved in MeOH (1.0 mL) and K_2CO_3 (4.0 mg, 32.0 µmol) was added and the reaction mixture was stirring was continued for 1-2 hours at room temperature. TLC analysis at this stage indicated complete consumption of starting materials. The reaction mixture was diluted with water and extracted with ethyl acetate. The organic layers were combined, dried with $MgSO_4$ and concentrated. The crude then underwent purification by flash column chromatography twice (50% EA/Pet.Ether) to give the desired compound (E)-**9** as a colourless oil (4.0 mg, 12.2 µmol, 29%, E:Z > 20:1).

Characterisation data of (E)-**9**: R_f = 0.40 (50% EA/Pet. Ether 40-60). ^{1}H NMR (500 MHz, $CDCl_3$) δ 6.22 (dt, J = 15.4, 7.4 Hz, 1H, **H4**), 5.60 (dq, J = 16.0, 1.7 Hz, 1H, **H3**), 4.58 (t, J = 9.6 Hz, 1H, **H12**), 4.14 (ddd, J = 9.7, 3.2, 2.1 Hz, 1H, **H9**), 4.02 – 3.94 (m, 2H, **H10**, **H7**), 3.88 (ddd, J = 11.8, 9.6, 2.6 Hz, 1H, **H13**), 3.72 (td, J = 7.2, 1.9 Hz, 1H, **H6**), 3.02 (ddd, J = 15.3, 9.7, 1.7 Hz, 1H, **H11'**), 2.80 (d, J = 2.3 Hz, 1H, **H1**), 2.59 (dtd, J = 15.1, 7.5, 1.5 Hz, 1H, **H5'**), 2.50 (dtd, J = 15.1, 7.5, 1.5 Hz, 1H, **H5**), 2.32 (dd, J = 15.2, 5.6 Hz, 1H, **H11**), 2.28 (d, J = 15.7 Hz, 1H, **H8'**), 2.03 – 1.91 (m, 2H, **H8**,

H14'), 1.69 (ddq, *J* = 14.2, 11.4, 7.1 Hz, 1H, **H14**), 1.05 (t, *J* = 7.3 Hz, 3H, **H15**). ^{13}C NMR (126 MHz, $CDCl_3$) δ 142.3 (**C4**), 111.3 (**C3**), 83.5 (**C13**), 83.4 (**C6**), 82.4 (**C2**), 78.8 (**C9**), 76.4 (**C1**), 70.5 (**C10**), 70.1 (**C7**), 51.8 (**C12**), 38.9 (**C11**), 33.1 (**C8**), 32.8 (**C5**), 23.2 (**C14**), 11.9 (**C15**). IR (ν_{max} cm^{-1}): 3403 (O–H stretchings, broad), 3292 (acetylene sp C–H stretching, medium), 2964-2878 (aliphatic sp^3 C–H stretchings, broad), 2102 (acetylene C≡C stretching, weak), 1064 (ether C–O–C antisymmetric stretching, strong), 651 (C–Br stretching, medium). LRMS (ESI) $[M+H]^+$: *m/z* 329.1 and 331.1. HRMS (ESI) $[M+H]^+$: calculated for *m/z* 329.07468 and 331.07264, found *m/z* 329.07477 and 331.07268. ($C_{15}H_{22}O_3{}^{79}Br$ and $C_{15}H_{22}O_3{}^{81}Br$). $[\alpha]_D^{25} = +17.5$ (c=0.12, MeOH), lit. $[\alpha]_D^{25} = +17.0$ $(c = 0.10, \mathrm{MeOH})$.[6] Spectroscopic data are in accordance with literature data.[6]

(1*S*,3*R*,4*S*,6*S*,7*S*,9*S*)-4-Bromo-3-ethyl-9-((*E*)-pent-2-en-4-yn-1-yl)-2,8-dioxabicyclo [5.2.1]decan-6-yl (*S*)-3,3,3-trifluoro-2-methoxy-2-phenylpropanoate S1

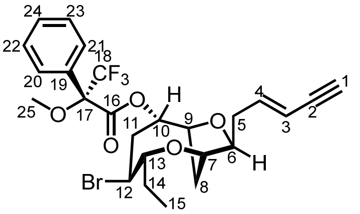

According to the procedures of Hoye[7]: Compound (*E*)-**9** (2.0 mg, 6.1 μmol) was mixed with (*S*)-Mosher Acid (4.3 mg, 18.2 μmol) and dissolved in dry CH_2Cl_2 (1.0 mL). *N*,*N*'-Dicyclohexylcarbodiimide (3.8 mg, 18.2 μmol) was added followed by DMAP (2.2 mg, 18.2 μmol). The reaction mixture was stirred at r.t. overnight and filtered through a plug of silica to remove any insoluble matter. The filtrate was concentrated and underwent purification by flash column chromatography (10% EA/Pet.Ether 40-60) to give the desired compound **S1** as a colourless oil (2.2 mg, 4.0 μmol, 66%). R_f = 0.56 (20% EA/Pet.Ether 40-60). ^{1}H NMR (500 MHz, $CDCl_3$) δ 7.55 – 7.47 (m, 2H, **H22**, **H23**), 7.48 – 7.37 (m, 3H, **H20**, **H21**, **H24**), 6.20 (dt, *J* = 15.3, 7.4 Hz, 1H, **H4**), 5.60 (dq, *J* = 15.9, 1.6 Hz, 1H, **H3**), 5.28 – 5.20 (m, 1H, **H10**), 4.18 (dd, *J* = 9.4, 4.0 Hz, 1H, **H9**), 4.14 (t, *J* = 9.6 Hz, 1H, **H12**), 3.99 (t, *J* = 2.5 Hz, 1H, **H7**), 3.86 (td, *J* = 10.5, 9.5, 2.6 Hz, 1H, **H13**), 3.73 (td, *J* = 7.1, 1.8 Hz, 1H, **H6**), 3.54 (s, 3H), 3.07 (ddd, *J* = 15.8, 9.8, 1.9 Hz, 1H, **H11'**), 2.81 (d, *J* = 2.2 Hz, 1H, **H1**), 2.59 (dtd, *J* = 15.1, 7.4, 1.3 Hz, 1H, **H5'**), 2.55 – 2.48 (m, 2H, **H5**, **H11**), 2.15 (d, *J* = 15.8 Hz, 1H, **H8'**), 2.01 (ddd, *J* = 15.5, 9.5, 3.3 Hz, 1H, **H8**), 1.90 (dqd, *J* = 15.1, 7.6, 2.6 Hz, 1H, **H14'**), 1.60 (ddd, *J* = 14.6, 11.3, 7.2 Hz, 1H, **H14**), 1.03 (t, *J* = 7.3 Hz, 3H, **H15**). ^{13}C NMR (126 MHz, $CDCl_3$) δ 165.1 (**C16**), 141.9 (**C4**), 132.2 (**C19**), 129.9 (**C24**), 128.8 (**C20, C21**), 127.5 (**C22, C23**), 123.5 (q, *J* = 289.4 Hz, **C18**), 111.5 (**C3**), 84.9 (q, *J* = 28.0 Hz, **C17**), 83.7 (**C6**), 83.5 (**C13**), 82.3 (**C2**), 76.6 (**C1**), 76.1 (**C9**), 74.6 (**C10**), 70.0 (**C7**), 55.6 (**C25**), 50.9 (**C12**), 35.8 (**C11**), 33.7 (**C8**), 32.6 (**C5**), 23.0 (**C14**), 11.8 (**C15**). ^{19}F NMR (471 MHz, $CDCl_3$) δ -71.4. Only NMR characterization data was obtained for Mosher ester analysis.

(1*S*,3*R*,4*S*,6*S*,7*S*,9*S*)-4-Bromo-3-ethyl-9-((*E*)-pent-2-en-4-yn-1-yl)-2,8-dioxabicyclo [5.2.1]decan-6-yl (*R*)-3,3,3-trifluoro-2-methoxy-2-phenylpropanoate S2

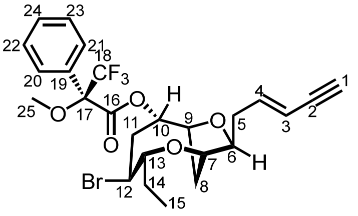

According to the procedures of Hoye[7]: Compound (*E*)-**9** (2.0 mg, 6.1 μmol) was mixed with (*R*)-Mosher Acid (4.3 mg, 18.2 μmol) and dissolved in dry CH_2Cl_2 (1.0 mL). *N*,*N*'-Dicyclohexylcarbodiimide (3.8 mg, 18.2 μmol) was added followed by DMAP (2.2 mg, 18.2 μmol). The reaction mixture was stirred at r.t. overnight and filtered through a plug of silica to remove any insoluble matter. The filtrate was concentrated and underwent purification by flash column chromatography (10% EA/Pet.Ether 40-60) to give the desired compound **S2** as a colourless oil (1.4 mg, 2.6 μmol, 42%). R_f = 0.67 (20% EA/Pet.Ether 40-60). ^{1}H NMR (500 MHz, $CDCl_3$) δ 7.56 – 7.48 (m, 2H, **H22**, **H23**), 7.46 – 7.39 (m, 3H, **H20**, **H21**, **H24**), 6.20 (dt, *J* = 15.4, 7.4 Hz, 1H, **H4**), 5.59 (dq, *J* = 15.7, 1.5 Hz, 1H, **H3**), 5.31 (t, *J* = 3.4 Hz, 1H, **H10**), 4.27 (t, *J* = 9.6 Hz, 1H, **H12**), 4.07 (dd, *J* = 9.6, 4.1 Hz, 1H, **H9**), 3.99 – 3.94 (m, 1H, **H7**), 3.87 (ddd, *J* = 11.8, 9.8, 2.6 Hz, 1H, **H13**), 3.70 (td, *J* = 7.4, 1.8 Hz, 1H, **H6**), 3.56 (s, 3H, **H25**), 3.13 (ddd, *J* = 15.8, 9.6, 1.8 Hz, 1H, **H11'**), 2.81 (d, *J* = 2.2 Hz, 1H, **H1**), 2.58 (dtd, *J* = 15.0, 7.4, 1.3 Hz, 1H, **H5'**), 2.54 – 2.46 (m, 1H, **H5**, **H11**), 2.07 (d, *J* = 15.6 Hz, 1H, **H8'**), 1.95 – 1.86 (m, 1H, **H8**, **H14'**), 1.61 (ddd, *J* = 14.6, 11.4, 7.2 Hz, 1H, **H14**), 1.03 (t, *J* = 7.3 Hz, 3H, **H15**). ^{13}C NMR (126 MHz, $CDCl_3$) δ165.1 (**C16**), 141.9 (**C4**), 132.1 (**C19**), 129.9 (**C24**), 128.8 (**C20**, **C21**), 127.5 (**C22**, **C23**), 111.5 (**C3**), 83.7 (**C6**), 83.4 (**C13**), 82.3 (**C2**), 76.6 (**C1**), 75.8 (**C9**), 74.1 (**C10**), 70.0 (**C7**), 55.7 (**C25**), 51.2 (**C12**), 36.2 (**C11**), 33.6 (**C8**), 32.6 (**C5**), 23.0 (**C14**), 11.8 (**C15**). ^{19}F NMR (471 MHz, $CDCl_3$) δ -71.3. Only NMR characterization data was obtained for Mosher ester analysis.

(1*S*,3*R*,4*S*,6*S*,7*S*,9*S*)-4-Bromo-3-ethyl-9-((*Z*)-p*ent*-2-en-4-yn-1-yl)-2,8-dioxabicyclo [5.2.1]decan-6-ol, laurefurenyne E (*Z*)-9

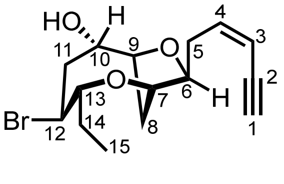

Synthesis from recycled NMR sample:

Compound (*Z*)-**5** (3.0 mg, 7.8 μmol) was dissolved in dry CD_2Cl_2 (0.3 mL) and the reaction mixture was cooled to -40 °C. $AgAl(pftb)_4{\cdot}CH_2Cl_2$ (10.0 mg, 8.6 μmol) in dry CD_2Cl_2 (0.3 mL) was then added at -40 °C. The reaction mixture was stirring was continued for 15 minutes at -40 °C in which precipitation of a brown substance was observed. The reaction mixture was then continued to stir at this temperature for 1 hour. An optional TLC analysis could be carried out at this point to confirm if the starting material is completely consumed. The reaction mixture was then cooled to -78 °C and equilibrated for 30 minutes. The reaction mixture was then filtered at -78 °C into a flame dried and

argon filled Young's NMR tube for subsequent NMR analyses. The colour of the filtrate could range from pale yellow to pale pink. After the analysis, the NMR sample was warmed to ca. -20 °C -0 °C, ejected from the spectrometer and incubated at -78 °C in a dry ice-acetone bath, and an excess of water was added. The sample was quickly removed from the bath, shaken vigorously and a pinch of $NaHCO_3$ was added. The sample was then returned to the bath and incubated for 15 minutes before gradually warming to room temperature. The reaction mixture was extracted with CH_2Cl_2, the organic layer was separated, dried with $MgSO_4$ and concentrated. The crude then underwent purification by flash column chromatography twice (50% EA/Pet. Ether 40-60) then (20% EA/CH_2Cl_2) to give the desired compound (*Z*)-**9** as a colourless oil (1.0 mg, 3.0 μmol, 39%).

Synthesis without NMR analysis:

Compound (*Z*)-**5** (11.3 mg, 29.0 μmol) was dissolved in dry CH_2Cl_2 (2.5 mL) and the reaction mixture was cooled to -40 °C. $AgAl(pftb)_4 \cdot CH_2Cl_2$ (37.1 mg, 32.0 μmol) in dry CH_2Cl_2 (1.5 mL) was then added at -40 °C. A precipitation of a brown substance was observed. The reaction was stirred at -40 °C for 1 hour, then cooled to -78 °C and excess H_2O was added followed by a pinch of $NaHCO_3$. The reaction mixture was then warmed to room temperature and excess tetrabutylammonium iodide was added to the reaction mixture, in which the colour of the organic layer changed from pale purple to pale yellow. The reaction mixture was extracted with CH_2Cl_2, the organic layer was separated, dried with $MgSO_4$ and concentrated. The crude then underwent purification by flash column chromatography (30% EA/Pet. Ether 40-60 then 50% EA/Pet. Ether 40-60) to recover unconsumed (*Z*)-**5** (4.0 mg, 10.2 μmol, 35%) and give the desired compound (*Z*)-**9** as a colourless oil (2.8 mg, 8.5 μmol, 45% brsm).

Synthesis from 12:

According to the procedures of Shirokane[8]: Compound **12** (20.0 mg, 66.0 μmol) was dissolved in 3:1 dioxane:H_2O (1.1 mL), then 2,6-lutidine (15.0 μL, 0.13 mmol) was added followed by a solution of 2.5% OsO_4 in *t*-BuOH (15.0 μL). $NaIO_4$ (55.0 mg, 0.26 mmol) was then added and the reaction mixture was stirring was continued for 1-2 hours. A TLC analysis at this stage showed complete consumption of starting material. The reaction mixture was then diluted with CH_2Cl_2 and H_2O followed by extraction with CH_2Cl_2. The organic layer was separated, dried with $MgSO_4$ and concentrated to give a crude brown oil. This crude brown oil was dried under high vacuum while the procedures below were carried out.

$Ph_3PCH_2I_2$ (140.0 mg, 0.26 mmol) was suspended in dry THF (2.5 mL) at room temperature and a solution of NaHMDS (0.24 mL, 1.0M solution in THF) was added. The reaction mixture was stirred at room temperature for 15 minutes in which the colour of the solution changed from bright yellow to deep orange. This solution was then cooled to -78 °C and HMPA (95.0 μL, 0.53 mmol) was added and stirring was continued for 15 minutes. The dried crude brown oil was then dissolved in dry THF (1.0 mL) and added to the reaction mixture. The reaction mixture was stirred at -78 °C for 30 minutes then room temperature for 1 hour. A TLC analysis at this stage showed complete consumption of starting material. The reaction was then quenched with sat. aq. NH_4Cl and extracted with ethyl acetate. The organic layer was separated, dried with $MgSO_4$ and concentrated. The crude mixture quickly underwent purification by flash column chromatography (50% EA/Pet. Ether 40-60), product R_f =

0.40 (50% EA/Pet. Ether 40-60). The purified vinyl iodide was then quickly used for the following transformations due to its instability.

The vinyl iodide (9.8 mg, 23.0 μmol) was dissolved in dry Et_3N (1.2 mL), then trimethylsilylacetylene (16.0 μL, 11.4 μmol) was added followed by CuI (4.4 mg, 23.0 μmol). The mixture was then degassed with dry argon for 15 minutes. $Pd(PPh_3)_4$ (4.0 mg, 3.5 μmol) was then added and the reaction mixture was stirred at room temperature with exclusion of light for 1 hour. A TLC analysis at this stage showed the complete consumption of starting material. K_2CO_3 (32.0 mg, 0.23 mmol) and MeOH (1.2 mL) were added to the reaction mixture and stirring was continued for 1-2 hours until completion. The reaction mixture was then diluted with H_2O and extracted with ethyl acetate. The organic layer was separated, dried with $MgSO_4$, filtered through a short plug of silica and concentrated. The resulting pale brown crude oil then underwent purification by flash column chromatography (50% EA/Pet. Ether 40-60) to give the desired compound (*Z*)-**9** as a colourless oil (7.5 mg, 22.8 μmol, 35% over 4 steps, *Z*:*E* > 15:1).

Characterisation data of (*Z*)-**9**: R_f = 0.35 (50% EA/Pet. Ether 40-60). ^1H NMR (500 MHz, $CDCl_3$) δ 6.06 (dtd, *J* = 10.8, 7.7, 0.8 Hz, 1H, **H4**), 5.55 (ddt, *J* = 10.9, 2.6, 1.4 Hz, 1H, **H3**), 4.59 (t, *J* = 9.6 Hz, 1H, **H12**), 4.15 (dd, *J* = 9.7, 4.0 Hz, 1H, **H9**), 4.01 (s, 1H, **H10**), 3.99 – 3.97 (m, 1H, **H7**), 3.90 (ddd, *J* = 11.7, 9.5, 2.6 Hz, 1H, **H13**), 3.77 (td, *J* = 7.1, 1.9 Hz, 1H, **H6**), 3.10 (dd, *J* = 2.4, 0.8 Hz, 1H, **H1**), 3.06 (ddd, *J* = 15.3, 9.7, 1.8 Hz, 1H, **H11'**), 2.83 (dtd, *J* = 13.8, 7.0, 1.2 Hz, 1H, **H5')**, 2.71 (dtd, *J* = 13.8, 7.0, 1.2 Hz, 1H, **H5**), 2.33 (dd, *J* = 15.2, 5.2 Hz, 1H, **H11**), 2.28 (d, *J* = 15.6 Hz, 1H, **H8'**), 2.03 – 1.92 (m, 2H, **H8**, **H14'**), 1.70 (ddq, *J* = 14.2, 11.2, 7.1 Hz, 1H, **H14**), 1.06 (t, *J* = 7.4 Hz, 3H, **H15**). ^{13}C NMR (126 MHz, $CDCl_3$) δ 141.7 (**C4**), 110.4 (**C3**), 83.4 (**C13**), 83.4 (**C6**), 82.1 (**C1**), 80.3 (**C2**), 78.8 (**C9**), 70.6 (**C10**), 70.3 (**C7**), 52.0 (**C12**), 38.9 (**C11**), 33.2 (**C8**), 30.3 (**C5**), 23.2 (**C14**), 12.0 (**C15**). IR (ν_{max} cm^{-1}): 3412 (O–H stretchings, broad), 3292 (acetylene sp C–H stretching, medium), 2964-2903 (aliphatic sp^3 C–H stretchings, broad), 2096 (acetylene C≡C stretching, weak), 1064 (ether C–O–C antisymmetric stretching, strong), 651 (C–Br stretching, medium). HRMS (APCI) $[M+H]^+$: calculated for *m/z* 329.07468 and 331.07264, found *m/z* 329.07431 and 331.07227. ($C_{15}H_{22}O_3{}^{79}Br$ and $C_{15}H_{22}O_3{}^{81}Br$). $[\alpha]_D^{25} = -4.7$ (c=0.17, MeOH, $Z:E = 7.8:1$) , $[\alpha]_D^{25} = -5.9$ (c=0.10, MeOH, $Z:E > 15:1$). Literature value: $[\alpha]_D^{25} = +11.0$ ($c = 0.10$, MeOH).[6] NMR spectroscopic data are in accordance with literature data.[6]

(1*S*,3*R*,4*S*,6*S*,7*S*,9*S*)-4-Bromo-3-ethyl-9-((*Z*)-pent-2-en-4-yn-1-yl)-2,8-dioxabicyclo [5.2.1]decan-6-yl (*S*)-3,3,3-trifluoro-2-methoxy-2-phenylpropanoate S3

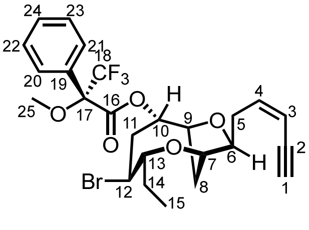

According to the procedures of Hoye[7]: Compound (*Z*)-**9** (2.0 mg, 6.1 μmol) was mixed with (*S*)-Mosher Acid (4.3 mg, 18.2 μmol) and dissolved in dry CH_2Cl_2 (1.0 mL). *N*,*N*'-Dicyclohexylcarbodiimide (3.8 mg, 18.2 μmol) was added followed by DMAP (2.2 mg, 18.2 μmol).

The reaction mixture was stirred at r.t. overnight and filtered through a plug of silica to remove any insoluble matter. The filtrate was concentrated and underwent purification by flash column chromatography (10% EA/Pet.Ether 40-60) to give the desired compound **S3** as a colourless oil (1.2 mg, 2.2 μmol, 36%). R_f = 0.65 (20% EA/Pet.Ether 40-60). ^{1}H NMR (500 MHz, $CDCl_3$) δ 7.53 – 7.49 (m, 2H, **H22**, **H23**), 7.45 – 7.36 (m, 3H, **H20**, **H21**, **H24**), 6.04 (ddd, *J* = 10.5, 7.7, 6.8 Hz, 1H, **H4**), 5.56 (ddt, *J* = 10.8, 2.4, 1.3 Hz, 1H, **H3**), 5.30 – 5.20 (m, 1H, **H10**), 4.19 (dd, *J* = 9.4, 4.0 Hz, 1H, **H9**), 4.15 (t, *J* = 9.9 Hz, 1H, **H12**), 3.99 (t, *J* = 2.6 Hz, 1H, **H7**), 3.87 (ddd, *J* = 11.9, 9.6, 2.6 Hz, 1H, **H13**), 3.78 (td, *J* = 7.1, 1.8 Hz, 1H, **H6**), 3.55 (s, 3H, **H25**), 3.17 – 3.05 (m, 2H, **H11'**, **H1**), 2.88 – 2.79 (m, 1H, **H5'**), 2.75 – 2.68 (m, 1H, **H5**), 2.52 (dd, *J* = 15.7, 5.1 Hz, 1H, **H11**), 2.15 (d, *J* = 15.5 Hz, 1H, **H8'**), 2.01 (ddd, *J* = 15.5, 9.5, 3.3 Hz, 1H, **H8**), 1.90 (dqd, *J* = 15.3, 7.6, 2.5 Hz, 1H, **H14'**), 1.61 (ddd, *J* = 14.6, 11.3, 7.2 Hz, 1H, **H14**), 1.03 (t, *J* = 7.3 Hz, 3H, **H15**). ^{13}C NMR (126 MHz, $CDCl_3$) δ 165.1 (**C16**), 141.3 (**C4**), 132.2 (**C19**), 129.9 (**C24**), 128.7 (**C20**, **C21**), 127.5 (**C22**, **C23**), 110.6 (**C3**), 84.9 (q, *J* = 28.0 Hz, **C17**), 83.8 (**C6**), 83.3 (**C13**), 82.2 (**C1**), 80.2 (**C2**), 76.1 (**C9**), 74.7 (**C10**), 70.3 (**C7**), 55.6 (**C25**), 51.1 (**C12**), 35.8 (**C11**), 33.8 (**C8**), 30.1 (**C5**), 23.1 (**C14**), 11.9 (**C15**). ^{19}F NMR (471 MHz, $CDCl_3$) δ -71.4. Only NMR characterization data was obtained for Mosher ester analysis.

(1*S*,3*R*,4*S*,6*S*,7*S*,9*S*)-4-Bromo-3-ethyl-9-((*Z*)-pent-2-en-4-yn-1-yl)-2,8-dioxabicyclo [5.2.1]decan-6-yl (*R*)-3,3,3-trifluoro-2-methoxy-2-phenylpropanoate S4

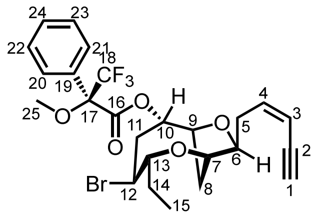

According to the procedures of Hoye[7]: Compound (*Z*)-**9** (2.0 mg, 6.1 μmol) was mixed with (*R*)-Mosher Acid (4.3 mg, 18.2 μmol) and dissolved in dry CH_2Cl_2 (1.0 mL). *N*,*N*'-Dicyclohexylcarbodiimide (3.8 mg, 18.2 μmol) was added followed by DMAP (2.2 mg, 18.2 μmol). The reaction mixture was stirred at r.t. overnight and filtered through a plug of silica to remove any insoluble matter. The filtrate was concentrated and underwent purification by flash column chromatography (10% EA/Pet.Ether 40-60) to give the desired compound **S4** as a colourless oil (1.5 mg, 2.8 μmol, 45%). R_f = 0.65 (20% EA/Pet.Ether 40-60). ^{1}H NMR (500 MHz, $CDCl_3$) δ 7.54 – 7.50 (m, 2H, **H22**, **H23**), 7.46 – 7.38 (m, 3H, **H20**, **H21**, **H24**), 6.04 (dtd, *J* = 10.7, 7.4, 0.7 Hz, 1H, **H4**), 5.56 (ddt, *J* = 10.9, 2.5, 1.3 Hz, 1H, **H3**), 5.32 (t, *J* = 4.7 Hz, 1H, **H10**), 4.28 (t, *J* = 9.6 Hz, 1H, **H12**), 4.07 (dd, *J* = 9.5, 4.0 Hz, 1H, **H9**), 3.97 (t, *J* = 2.6 Hz, 1H, **H7**), 3.89 (ddd, *J* = 11.8, 9.6, 2.6 Hz, 1H, **H13**), 3.75 (td, *J* = 7.1, 1.8 Hz, 1H, **H6**), 3.56 (s, 3H, **H25**), 3.17 (ddd, *J* = 15.8, 9.7, 1.9 Hz, 1H, **H11'**), 3.11 (dd, *J* = 2.2, 0.9 Hz, 1H, **H1**), 2.83 (dtd, *J* = 14.1, 7.7, 1.1 Hz, 1H, **H5'**), 2.71 (dtd, *J* = 13.9, 7.4, 1.2 Hz, 1H, **H5**), 2.50 (dd, *J* = 15.7, 5.2 Hz, 1H, **H11**), 2.08 (d, *J* = 15.6 Hz, 1H, **H8'**), 1.97 – 1.85 (m, 2H, **H8**, **H14'**), 1.62 (ddd, *J* = 14.5, 11.3, 7.2 Hz, 1H, **H14**), 1.04 (t, *J* = 7.3 Hz, 3H, **H15**). ^{13}C NMR (126 MHz, $CDCl_3$) δ 165.1 (**C16**), 141.3 (**C4**), 132.2 (**C19**), 129.9 (**C24**), 128.8 (**C20**, **C21**), 127.5 (**C22**, **C23**), 110.6 (**C3**), 83.7 (**C6**), 83.3 (**C13**), 82.2 (**C1**), 80.2 (**C2**), 75.8 (**C9**), 74.1 (**C10**),

70.3 (**C7**), 55.7 (**C25**), 51.4 (**C12**), 36.2 (**C11**), 33.6 (**C8**), 30.1 (**C5**), 23.1 (**C14**), 11.9 (**C15**). ^{19}F NMR (471 MHz, $CDCl_3$) δ -71.3. Only NMR characterization data was obtained for Mosher ester analysis.

(1*S*,3*R*,6*S*,7*S*,9*S*,*Z*)-9-Allyl-3-ethyl-2,8-dioxabicyclo[5.2.1]dec-4-en-6-ol 21

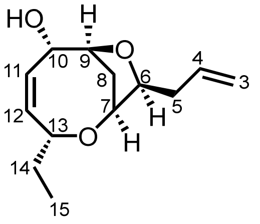

Compound **12** (5.0 mg, 16.0 μmol) was dissolved in DMF (0.8 mL). CsOAc (47.0 mg, 0.25 mmol) was added and the reaction mixture was stirred at 90˚C overnight. TLC analysis suggested that the reaction was complete. Crude NMR analysis revealed that the major product was the elimination product **21**. The crude mixture then underwent purification by flash column chromatography (50% EA/CH_2Cl_2) to give the desired compound **21** in moderate purity, as an inseperable mixture with an unknown minor component in ca. 6:1 ratio (3.0 mg, 13.3 μmol, 84%). R_f = 0.40 (50% EA/Pet. Ether 40-60). ^{1}H NMR (500 MHz, $CDCl_3$) δ 5.87 (ddt, *J* = 17.3, 10.2, 7.0 Hz, 1H, **H4**), 5.60 – 5.51 (m, 2H, **H11**, **H12**), 5.17 (dq, *J* = 17.2, 1.6 Hz, 1H, **H3'**), 5.09 (ddt, *J* = 10.1, 2.1, 1.1 Hz, 1H, **H3**), 4.82 (s, 1H, **H10**), 4.29 – 4.23 (m, 2H, **H13**, **H9**), 4.16 (dd, *J* = 4.5, 2.8 Hz, 1H, **H7**), 3.78 (td, *J* = 7.2, 2.8 Hz, 1H, **H6**), 2.63 (dt, *J* = 14.1, 0.9 Hz, 1H, **H8'**), 2.48 (m, 2H, **H5**), 1.97 (d, *J* = 4.4 Hz, 1H, O**H**), 1.94 (dt, *J* = 8.7, 4.8 Hz, 1H, **H8**), 1.74 (dt, *J* = 13.8, 7.4 Hz, 1H, **H14'**), 1.53 – 1.48 (m, 1H, **H14**), 0.94 (t, *J* = 7.4 Hz, 3H, **H15**). ^{13}C NMR (126 MHz, $CDCl_3$) δ 136.2 (**C11**), 135.0 (**C4**), 133.5 (**C12**), 117.3 (**C3**), 84.8 (**C9**), 83.4 (**C6**), 76.7 (**C10**), 75.4 (**C7**), 75.0 (**C13**), 34.2 (**C8**), 33.7 (**C5**), 27.8 (**C14**), 10.4 (**C15**). IR (ν_{max} cm^{-1}): 3413 (O–H stretching, broad), 2930 (aliphatic sp^3 C–H stretchings, broad), 1642 (C=C stretching, medium), 1045 (ether C–O–C antisymmetric stretching, strong). LRMS (ESI) [M+Na]$^+$: *m/z* 247.1. HRMS (ESI) [M+Na]$^+$: calculated for *m/z* 247.13047, found *m/z* 247.13046. ($C_{13}H_{20}O_3Na$). $[\alpha]_D^{25} = -10.0$ (c=0.10, $CHCl_3$).

(1*S*,3*R*,4*R*,6*S*,7*S*,9*S*)-9-Allyl-3-ethyl-2,8-dioxabicyclo[5.2.1]decane-4,6-diol 22

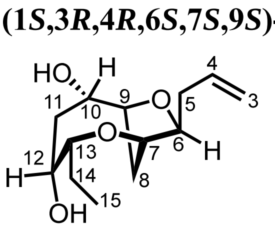

The compound **12** (3.0 mg, 10.0 μmol) was dissolved in DMF (0.1 mL) and caesium trifluoroacetate (12.3 mg, 50.0 μmol) was added. The reaction was then stirred at 120 °C overnight. TLC analysis at this stage indicated complete consumption of starting materials. The reaction was diluted with water and extracted with ethyl acetate. The organic layers were combined, dried with anhydrous $MgSO_4$ and concentrated. The crude then underwent purification by flash column chromatography (100% EA) to give the desired compound **22** as a white solid (1.5 mg, 6.2 μmol, 62%). R_f = 0.31 (100% EA). ^{1}H NMR (500 MHz, $CDCl_3$) δ 5.85 (ddt, *J* = 17.2, 10.2, 7.0 Hz, 1H, **H4**), 5.15 (dq, *J* = 17.1, 1.6 Hz, 1H,

H3'), 5.07 (ddt, J = 10.3, 2.3, 1.2 Hz, 1H, **H3**), 4.30 (ddd, J = 8.4, 3.5, 1.4 Hz, 1H, **H9**), 4.27 (ddd, J = 7.0, 5.2, 2.7 Hz, 1H, **H12**), 4.09 (q, J = 3.9 Hz, 1H, **H10**), 4.02 (dd, J = 4.1, 2.1 Hz, 1H, **H7**), 3.79 (dt, J = 8.5, 4.9 Hz, 1H, **H13**), 3.76 (td, J = 7.1, 2.1 Hz, 1H, **H6**), 3.29 (s, 1H, O**H**), 2.97 (d, J = 15.1 Hz, 1H, **H8'**), 2.74 (s, 1H, O**H**), 2.53 (dt, J = 13.9, 6.9 Hz, 1H, **H5'**), 2.46 (dt, J = 14.0, 7.1 Hz, 1H, **H5**), 2.24 – 2.17 (m, 2H, **H11**), 1.96 (ddd, J = 14.4, 10.0, 4.0 Hz, 1H, **H8**), 1.77 (ddq, J = 14.6, 9.2, 7.3 Hz, 1H, **H14'**), 1.60 (dqd, J = 14.7, 7.3, 2.1 Hz, 1H, **H14**), 1.01 (t, J = 7.4 Hz, 3H, **H15**). ^{13}C NMR (126 MHz, $CDCl_3$) δ 135.2 (**C4**), 117.1 (**C3**), 83.9 (**C6**), 79.7 (**C9**), 79.2 (**C13**), 73.5 (**C10**), 73.0 (**C12**), 71.0 (**C7**), 33.8 (**C5**), 32.1 (**C8**), 31.1 (**C11**), 23.4 (**C14**), 11.4 (**C15**). IR (ν_{max} cm^{-1}): 3336 (O–H stretchings, broad), 2963-2857 (aliphatic sp^3 C–H stretchings, broad), 1642 (alkene C=C stretching, medium), 1078 (ether C–O–C antisymmetric stretching, strong). LRMS (ESI) [M+H]$^+$: 243.2. HRMS (ESI) [M+H]$^+$: calculated for *m/z* 243.15909, found *m/z* 243.15933. ($C_{13}H_{23}O_4$). $[\alpha]_D^{25} = +27.0$ (c=0.10, $CHCl_3$). Melting point: 118.4 °C. The structure of compound **22** was further confirmed by X-ray crystallography (CCDC 2005094).

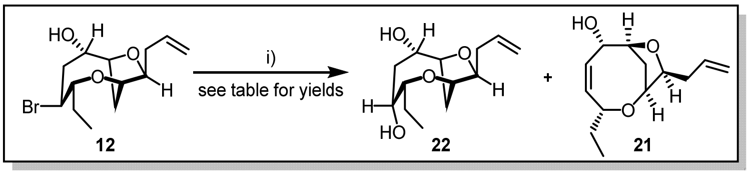

Entry	Nucleophile	Equivalents	T/°C	Time/hrs	Additives	Results
**1**	$NaNO_2$	15	90	12	-	No reaction
**2**	$NaNO_2$	15	120	36	-	Trace **21** was observed
**3**	LiOAc	15	90	12.	-	No reaction
**4**	LiOAc	15	120	12	-	Limited conversion, messy crude ^{1}H NMR
**5**	CsOAc	15	90	36	-	**21** in **84%** yield, moderate purity
**6**	KNO_2	5	r.t.	12	cat. 18-crown-6	No reaction
**7**	KNO_2	5	90	12	cat. 18-crown-6	Limited conversion
**8**	KNO_2	5	120	12	cat. 18-crown-6	Decent conversion, messy crude ^{1}H NMR
**9**	CsTFA	5	120	12	-	**22** in **62%** yield, trace **21**

Table S1. Optimisation of the reaction conditions for the desired S_N2 reaction.

Reagents and conditions: **i)** Nucleophile (x equivalents), additives, DMF, T °C, time.

(1*S*,3*R*,4*R*,6*S*,7*S*,9*S*)-3-Ethyl-9-((*E*)-pent-2-en-4-yn-1-yl)-2,8-dioxabicyclo[5.2.1] decane-4,6-diol laurefurenyne D (*E*)-10

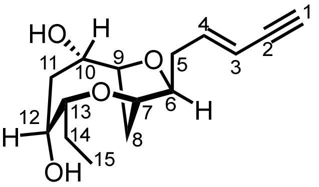

Synthesis from (*E*)-9:

The compound laurefurenyne F (*E*)-**9** (4.0 mg, 12.0 μmol) was dissolved in DMF (0.12 mL), and caesium trifluoroacetate (15.0 mg, 60.0 μmol) was added. The reaction mixture was then stirred at 120 °C for 12 hours. TLC analysis at this stage indicated the complete consumption of starting material. The reaction mixture was cooled to room temperature and quenched with water. The aqueous layer was extracted with ethyl acetate and all the organic layers were combined and dried with $MgSO_4$. The crude was concentrated and underwent purification by flash column chromatography (50% EA/Pet. Ether 40-60 then 100% EA) to give the desired compound (*E*)-**10** as a colourless oil (1.2 mg, 4.5 μmol, 38%). The compound was crystallised by slow diffusion of pentane into a solution of (*E*)-**10** in ethyl acetate for X-ray crystallographic analysis.

Synthesis from 22:

According to the procedures of Shepherd[9]: The compound **22** (3.0 mg, 12.0 μmol) was dissolved in CH_2Cl_2 (0.4 mL) and degassed with argon. Crotonaldehyde (10.0 μL, 0.12 mmol) was added followed by Grubb's 2nd generation catalyst (0.5 mg, 0.6 μmol). The reaction mixture was then stirred at 40 °C for 1 hour. TLC analysis at this stage confirmed the complete consumption of starting materials. DMSO (0.1 mL) was added and stirred open to air overnight. All volatiles were then removed under reduced pressure, and the residue quickly underwent purification by flash column chromatography (100% EA) to give the enal (3.4 mg, 12.0 μmol, quant.) which was carried forward immediately to the next step.

According to the procedures of Kim[2]: A solution of $TMSCHN_2$ in Et_2O (2.0M, 60.0 μL, 0.12 mmol) was added to a solution of LDA (0.5M, 0.24 mL, 0.12 mmol) at -78 °C dropwise. The enal (3.4 mg, 12.0 μmol) was then dissolved in THF (0.3 mL) and added to the $TMSCLiN_2$ solution at -78 °C dropwise. The reaction mixture was stirred at -78 °C for 1 hour then 0 °C for 1 hour. TLC analysis at this stage confirmed the complete consumption of starting materials. The reaction mixture was quenched with aq. HCl (1.0M) and extracted with ethyl acetate. The organic layers were combined, dried with anhydrous $MgSO_4$ and concentrated. The crude then underwent purification by flash column chromatography (50% EA/Pet.Ether 40-60 then 100% EA) to give the desired compound (*E*)-**10** as a colourless oil (2.6 mg, 9.8 μmol, 81%, *E*:*Z* > 20:1).

Characterisation of (*E*)-**10**: R_f = 0.43 (100% EA). ^{1}H NMR (500 MHz, $CDCl_3$) δ 6.26 (dt, *J* = 15.3, 7.4 Hz, 1H, **H4**), 5.59 (dq, *J* = 15.9, 1.7 Hz, 1H, **H3**), 4.32 (ddd, *J* = 7.3, 5.5, 1.7 Hz, 1H, **H12**), 4.29 (dd, *J* = 9.6, 4.0 Hz, 1H, **H9**), 4.07 (s, 1H, **H10**), 3.99 (dd, *J* = 3.7, 2.0 Hz, 1H, **H7**), 3.79 – 3.68 (m, 2H, **H6**, **H13**), 3.37 (s, 1H, O**H**), 3.02 (d, *J* = 15.0 Hz, 1H, **H8'**), 2.80 (d, *J* = 2.2 Hz, 1H, **H1**), 2.77 (s, 1H, O**H**), 2.58 (dtd, *J* = 14.7, 7.4, 1.5 Hz, 1H, **H5'**), 2.50 (dtd, *J* = 14.9, 7.4, 1.1 Hz, 1H, **H5**), 2.25 – 2.14 (m, 2H, **H11**), 1.94 (ddd, *J* = 14.3, 9.9, 3.8 Hz, 1H, **H8'**), 1.79 (ddq, *J* = 14.6, 9.5, 7.3 Hz, 1H,

H14'), 1.60 (ddd, *J* = 14.5, 7.5, 2.3 Hz, 1H, **H14**), 1.02 (t, *J* = 7.4 Hz, 3H, **H15**). ^{13}C NMR (126 MHz, $CDCl_3$) δ 142.7 (**C4**), 111.1 (**C3**), 83.2 (**C6**), 82.4 (**C2**), 79.9 (**C9**), 79.5 (**C13**), 76.4 (**C1**), 73.6 (**C10**), 73.2 (**C12**), 70.4 (**C7**), 33.0 (**C5**), 31.9 (**C8**), 30.2 (**C11**), 22.6 (**C14**), 11.5 (**C15**). IR (ν_{max} cm^{-1}): 3291 (O–H stretching overlapped with acetylene sp C–H stretching, broad), 2921 (aliphatic sp^3 C–H stretchings, broad), 2100 (acetylene C≡C stretching, weak), 1664 (C=C stretching, medium), 1084 (ether C–O–C antisymmetric stretching, strong). LRMS (ESI) $[M+H]^+$: *m/z* 267.2. HRMS (ESI) $[M+H]^+$: calculated for *m/z* 267.15909, found *m/z* 267.15919 ($C_{15}H_{23}O_4$). $[\alpha]_D^{25}$ = +14.0 (c=0.10, MeOH). Literature value: $[\alpha]_D^{25} = +32.0\ (c = 0.10, \mathrm{MeOH})$.[6] Spectroscopic data are in accordance with literature data.[6] The structure of compound (*E*)-**10** was further confirmed by X-ray crystallography (CCDC 2005091). No melting point was obtained for this compound.

(1*S*,3*R*,4*R*,6*S*,7*S*,9*S*)-3-Ethyl-9-((*Z*)-pent-2-en-4-yn-1-yl)-2,8-dioxabicyclo[5.2.1] decane-4,6-diol laurefurenyne C (*Z*)-10

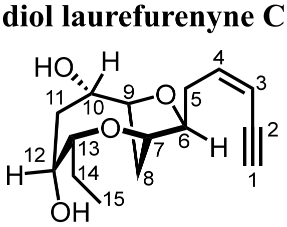

Synthesis from (*Z*)-9:

The compound laurefurenyne E (*Z*)-**9** (4.0 mg, 12.0 µmol) was dissolved in DMF (0.12 mL), and caesium trifluoroacetate (30.0 mg, 0.12 mmol) was added. The reaction mixture was then stirred at 120 °C for 12 hours. TLC analysis at this stage indicated the complete consumption of starting material. The reaction mixture was cooled to room temperature and quenched with water. The aqueous layer was extracted with ethyl acetate and all the organic layers were combined and dried with $MgSO_4$. The crude was concentrated and underwent purification by flash column chromatography (50% EA/Pet. Ether 40-60 then 100% EA) to give the desired compound (*Z*)-**10** as a colourless oil (0.8 mg, 2.8 µmol, 23%).

Synthesis from 22:

According to the procedures of Shirokane[8]: Compound **22** (3.0 mg, 12.0 µmol) was dissolved in 3:1 dioxane:H_2O (0.2 mL), then 2,6-lutidine (3.0 µL, 24.0 µmol) was added followed by a solution of 2.5% OsO_4 in *t*-BuOH (3.0 µL). $NaIO_4$ (10.0 mg, 47.0 µmol) was then added and the reaction mixture was stirring was continued for 1-2 hours. A TLC analysis at this stage showed complete consumption of starting material. The reaction mixture was then diluted with CH_2Cl_2 and H_2O followed by extraction with CH_2Cl_2. The organic layer was separated, dried with $MgSO_4$ and concentrated to give a crude brown oil. This crude brown oil was dried under high vacuum while the procedures below were carried out.

$Ph_3PCH_2I_2$ (32.0 mg, 60.0 µmol) was suspended in dry THF (0.3 mL) at room temperature and a solution of NaHMDS (55.0 µL, 1.0M solution in THF) was added. The reaction mixture was stirred at room temperature for 15 minutes in which the colour of the solution changed from bright yellow to deep orange. This solution was then cooled to -78 °C and HMPA (21.0 µL, 0.12 mmol) was added and stirring was continued for 15 minutes. The dried crude brown oil was then dissolved in dry THF

(0.3 mL) and added to the reaction mixture. The reaction mixture was stirred at -78 °C for 30 minutes then room temperature for 1 hour. A TLC analysis at this stage showed complete consumption of starting material. The reaction was then quenched with sat. aq. NH_4Cl and extracted with ethyl acetate. The organic layer was separated, dried with $MgSO_4$ and concentrated. The crude mixture quickly underwent purification by flash column chromatography (70% EA/Pet. Ether 40-60 then 100% EA), product R_f = 0.40 (100% EA). The purified vinyl iodide was then quickly used for the following transformations due to its instability.

The vinyl iodide (1.5 mg, 4.1 μmol) was dissolved in dry Et_3N (0.3 mL), then trimethylsilylacetylene (3.0 μL, 20.0 μmol) was added followed by CuI (0.8 mg, 4.1 μmol). The mixture was then degassed with dry argon for 15 minutes. $Pd(PPh_3)_4$ (0.7 mg, 0.61 μmol) was then added and the reaction mixture was stirred at room temperature with exclusion of light for 1 hour. A TLC analysis at this stage showed the complete consumption of starting material. K_2CO_3 (6.0 mg, 41.0 μmol) and MeOH (0.3 mL) were added to the reaction mixture and stirring was continued for 1-2 hours until completion. The reaction mixture was then diluted with H_2O and extracted with ethyl acetate. The organic layer was separated, dried with $MgSO_4$, filtered through a short plug of silica and concentrated. The resulting pale brown crude oil then underwent purification by flash column chromatography (100% EA) to give the desired compound (*Z*)-**10** as a colourless oil (1.0 mg, 3.8 μmol, 32% over 4 steps, *Z:E* > 15:1).

Characterisation of (*Z*)-**10**: R_f = 0.40 (100% EA). ^{1}H NMR (500 MHz, $CDCl_3$) δ 6.10 (dt, *J* = 10.9, 7.4 Hz, 1H, **H4**), 5.55 (dd, *J* = 10.8, 2.1 Hz, 1H, **H3**), 4.32 – 4.29 (m, 1H, **H9**), 4.29 – 4.27 (m, 1H, **H12**), 4.09 (s, 1H, **H10**), 4.02 (t, *J* = 3.1 Hz, 1H, **H7**), 3.84 – 3.80 (m, 1H, **H13**), 3.80 – 3.77 (m, 1H, **H6**), 3.32 (d, *J* = 8.2 Hz, 1H, O**H**), 3.10 (d, *J* = 2.3 Hz, 1H, **H1**), 2.98 (d, *J* = 15.0 Hz, 1H, **H8'**), 2.85 (dt, *J* = 14.3, 7.2 Hz, 1H, **H5'**), 2.76 – 2.67 (m, 2H, O**H**, **H5**), 2.29 – 2.14 (m, 2H, **H11**), 1.96 (ddd, *J* = 14.5, 10.0, 4.0 Hz, 1H, **H8**), 1.78 (dp, *J* = 14.7, 8.5, 7.4 Hz, 1H, **H14'**), 1.64 – 1.59 (m, 1H, **H14**), 1.02 (t, *J* = 7.4 Hz, 3H, **H15**). ^{13}C NMR (126 MHz, $CDCl_3$) δ 142.0 (**C4**), 110.2 (**C3**), 83.2 (**C6**), 82.0 (**C1**), 80.3 (**C2**), 79.7 (**C9**), 79.2 (**C13**), 73.5 (**C10**), 72.9 (**C12**), 71.2 (**C7**), 32.1 (**C8**), 31.0 (**C11**), 30.5 (**C5**), 23.3 (**C14**), 11.4 (**C15**). IR (ν_{max} cm^{-1}): 3350 (O–H stretching , broad), 3291 (acetylene C–H stretching, medium), 2963-2854 (aliphatic sp^3 C–H stretchings, broad), 2096 (C≡C stretching, weak), 1668 (C=C stretching, medium), 1084 (ether C–O–C antisymmetric stretching, strong). LRMS (ESI) $[M+Na]^+$: *m/z* 289.1. HRMS (ESI) $[M+Na]^+$: calculated for *m/z* 289.14103, found *m/z* 289.14105 ($C_{15}H_{22}O_4Na$). $[\alpha]_D^{25} = +10.8$ (c=0.13, MeOH) . Literature value: $[\alpha]_D^{25} = +20.0$ (c=0.10, MeOH).[6] Spectroscopic data are in accordance with literature data.[6]

5-(((1*S*,3*S*,4*R*,6*S*,7*S*,9*S*)-9-Allyl-4-bromo-3-ethyl-2,8-dioxabicyclo[5.2.1]decan-6-yl)thio)-1-phenyl-1*H*-tetrazole S5

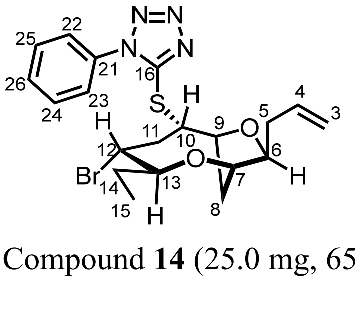

Compound **14** (25.0 mg, 65.0 μmol) was dissolved in dry CH_2Cl_2 (6.5 mL) and the reaction mixture

was cooled to -40 °C. $TiCl_4$ (0.26 mL, 0.5 M in CH_2Cl_2, 195.0 μmol) was added followed by a solution of $AgAl(pftb)_4{\cdot}CH_2Cl_2$ (226.0 mg, 130.0 μmol, in 1.0 mL CH_2Cl_2) immediately. The reaction mixture was stirred at -40 °C for 2 hours, then cooled to -78 °C. PTSH (1-phenyl-1*H*-tetrazole-5-thiol) (116.0 mg, 0.65 mmol) was added to the reaction mixture and stirring was continued for 1 hour. The reaction mixture was then quenched with sat. aq. $NaHCO_3$ then warmed to r.t. The quenched reaction mixture was then extracted with CH_2Cl_2, the organic layers combined and dried with $MgSO_4$ and concentrated. The crude then underwent purification by flash column chromatography (30% EA/Pet. Ether 40-60) to give the desired compound **S5** as a white foam (23.0 mg, 49.4 μmol, 76%). R_f = 0.60 (50% EA/Pet. Ether 40-60). ^{1}H NMR (500 MHz, $CDCl_3$) δ 7.64 – 7.48 (m, 5H, **H22**-**H26**), 5.80 (ddt, *J* = 17.2, 10.2, 7.1 Hz, 1H, **H4**), 5.14 (dq, *J* = 17.1, 1.6 Hz, 1H, **H3'**), 5.06 (ddt, *J* = 10.2, 2.1, 1.1 Hz, 1H, **H3**), 4.32 (dd, *J* = 7.5, 5.7 Hz, 1H, **H9**), 4.11 (p, *J* = 1.1 Hz, 1H, **H7**), 4.06 (td, *J* = 10.3, 6.3 Hz, 1H, **H12**), 4.00 (t, *J* = 7.3 Hz, 1H, **H10**), 3.88 (ddd, *J* = 8.6, 6.4, 2.5 Hz, 1H, **H6**), 3.58 (ddd, *J* = 10.8, 9.0, 2.5 Hz, 1H, **H13**), 2.76 – 2.65 (m, 3H, **H8'**, **H11**), 2.55 – 2.48 (m, 1H, **H5'**), 2.48 – 2.41 (m, 1H, **H5**), 2.17 (dqd, *J* = 14.8, 7.5, 2.5 Hz, 1H, **H14'**), 1.96 (ddd, *J* = 14.3, 7.5, 2.3 Hz, 1H, **H8**), 1.57 (ddd, *J* = 14.0, 9.0, 7.1 Hz, 1H, **H14**), 0.98 (t, *J* = 7.3 Hz, 3H, **H15**). ^{13}C NMR (126 MHz, $CDCl_3$) δ 153.9 (**C16**), 134.3 (**C4**), 133.8 (**C21**), 130.2 (**C26**), 129.9 (**C24**, **C25**), 123.9 (**C22**, **C23**), 117.6 (**C3**), 83.8 (**C6**), 81.1 (**C13**), 80.4 (**C9**), 77.0 (**C7**), 55.5 (**C10**), 53.3 (**C12**), 34.5 (**C11**), 34.4 (**C8**, **C5**), 28.4 (**C14**), 9.8 (**C15**). IR (ν_{max} cm^{-1}): 3009-2876 (aliphatic sp^3 C–H stretchings, broad), 1642 (C=C stretching, medium), 1597 (tetrazole C=C and C=N stretchings, medium), 1499 (tetrazole C=N and N=N stretchings, strong), 1061 (ether C–O–C antisymmetric stretching, strong), 756 (monosubstituted benzene C–H out of plane deformation, strong), 693 (C–Br stretching, medium). LRMS (ESI) $[M+H]^+$: *m/z* 465.1 and 467.1. HRMS (ESI) $[M+H]^+$: calculated for *m/z* 465.09544 and 467.09339, found *m/z* 465.09492 and 467.09270. ($C_{20}H_{26}O_2N_4{}^{79}Br^{32}S$ and $C_{20}H_{26}O_2N_4{}^{81}Br^{32}S$). $[\alpha]_D^{25}$ = +84.4 (c=0.32, $CHCl_3$). The structure of **S5** was assigned by analysis of its ^{1}H NMR spectrum, ^{13}C NMR spectrum, ^{1}H-^{1}H COSY spectrum, ^{1}H-^{13}C HSQC spectrum, ^{1}H-^{13}C HMBC spectrum, and by analogy with the assignment of compound **17** and their correspond chloride **16** as prepared previously.[1]

5-(((1*S*,3*S*,4*R*,6*S*,7*S*,9*S*)-9-Allyl-4-bromo-3-ethyl-2,8-dioxabicyclo[5.2.1]decan-6-yl)sulfonyl)-1-phenyl-1*H*-tetrazole 30

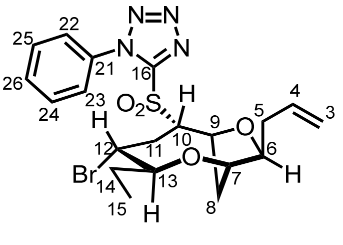

Compound **S5** (20.0 mg, 43.0 μmol) was dissolved in EtOH (0.4 mL) and the reaction mixture was cooled to 0 °C. A solution of 30% aq. H_2O_2 (40 μL) was mixed with $Mo_7O_{24}(NH_4)_6{\cdot}4H_2O$ (8.0 mg, 6.5 μmol) and the reaction mixture was cooled to 0 °C. The solution containing compound **7.9** was then mixed with the oxidant solution at 0 °C and then gradually warmed to r.t. and stirred overnight. TLC analyses have revealed that the reaction was not complete, thus a further addition of 30% aq.

H_2O_2 (40 μL) and $Mo_7O_{24}(NH_4)_6{\cdot}4H_2O$ (8.0 mg, 6.5 μmol) as an oxidant solution at 0 °C was performed. The reaction was stirred at r.t. for a further 7 hours where TLC analysis indicated completion of the reaction. The reaction mixture was diluted with ethyl acetate, then the organic layer was separated, washed with sat. aq. $Na_2S_2O_3$ and concentrated. The crude then underwent purification by flash column chromatography (30% EA/Pet. Ether 40-60) to give the desired compound **30** as a crispy white foam (16.4 mg, 33.0 μmol, 77%). R_f = 0.7 (50% EA/Pet. Ether). ^1H NMR (500 MHz, $CDCl_3$) δ 7.71 – 7.65 (m, 2H, **H22**, **H23**), 7.65 – 7.62 (m, 1H, **H26**), 7.62 – 7.57 (m, 2H, **H24**, **H25**), 5.73 (ddt, *J* = 17.3, 10.2, 7.1 Hz, 1H, **H4**), 5.12 (dq, *J* = 17.1, 1.6 Hz, 1H, **H3'**), 5.07 (ddt, *J* = 10.2, 2.1, 1.1 Hz, 1H, **H3**), 4.79 (dd, *J* = 7.4, 5.3 Hz, 1H, **H9**), 4.08 (q, *J* = 1.9 Hz, 1H, **H7**), 3.91 – 3.78 (m, 3H, **H6**, **H10**, **H12**), 3.56 (ddd, *J* = 10.8, 8.7, 2.5 Hz, 1H, **H13**), 2.80 (dd, *J* = 15.5, 4.7 Hz, 1H, **H11'**), 2.61 (d, *J* = 14.4 Hz, 1H, **H8'**), 2.42 – 2.37 (m, 2H, **H5**), 2.33 (ddd, *J* = 15.4, 12.0, 9.7 Hz, 1H, **H11**), 2.16 (dqd, *J* = 14.0, 7.3, 2.5 Hz, 1H, **H14'**), 2.04 (ddd, *J* = 14.4, 7.7, 2.1 Hz, 1H, **H8**), 1.61 – 1.55 (m, 1H, **H14**), 0.97 (t, *J* = 7.3 Hz, 3H, **H15**). ^{13}C NMR (126 MHz, $CDCl_3$) δ 152.7 (**C16**), 133.8 (**C4**), 133.1 (**C21**), 131.7 (**C26**), 129.7 (**C24**, **C25**), 125.8 (**C22**, **C23**), 117.9 (**C3**), 84.0 (**C6**), 81.2 (**C13**), 76.8 (**C7**), 75.3 (**C10**), 73.5 (**C9**), 51.4 (**C12**), 35.0 (**C11**), 34.1 (**C8**, **C5**), 28.2 (**C14**), 9.6 (**C15**). IR (ν_{max} cm^{-1}): 2961-2853 (aliphatic sp^3 C–H stretchings, broad), 1642 (C=C stretching, medium), 1595 (tetrazole C=C and C=N stretchings, medium), 1497 (tetrazole C=N and N=N stretchings, strong), 1346 (sulfone SO_2 antisymmetric stretching, strong), 1152 (sulfone SO_2 symmetric stretching, strong), 1063 (ether C–O–C antisymmetric stretching, strong), 760 (monosubstituted benzene C–H out of plane deformation, strong), 689 (C–Br stretching, medium). LRMS (ESI) $[M+H]^+$: *m/z* 497.1 and 499.1. HRMS (ESI) $[M+H]^+$: calculated for *m/z* 497.08527 and 499.08322, found *m/z* 497.08535 and 499.08308. ($C_{20}H_{26}O_4N_4{}^{79}Br^{32}S$ and $C_{20}H_{26}O_4N_4{}^{81}Br^{32}S$). $[\alpha]_D^{25} = +36.0$ (c=0.20, $CHCl_3$). The structure of **30** was assigned by analysis of its ^1H NMR spectrum, ^{13}C NMR spectrum, ^1H-^1H COSY spectrum, ^1H-^{13}C HSQC spectrum, and by analogy with the assignment of **S5**.

5-(((1*S*,2*R*,4*S*,5*R*,7*S*,8*S*)-8-Allyl-5-ethyl-6,9-dioxatricyclo[5.2.1.0^{2,4}]decan-2-yl)sulfonyl)-1-phenyl-1*H*-tetrazole 32

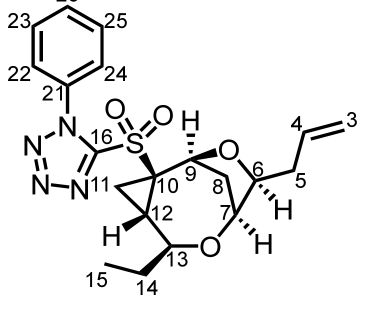

Compound **30** (6.5 mg, 13.0 μmol) was dissolved in dry THF (0.2 mL) and the reaction mixture was cooled to -78 °C. NaHMDS (0.14 mL, 0.1 M in THF, 14.0 μmol) was added at -78 °C where the solution turned from colourless to bright yellow. The reaction mixture was then gradually warmed to r.t. and the solution decolourised and became cloudy over 15 minutes. TLC analysis at this point indicated completion of reaction. The reaction mixture was quenched with sat. aq. NH_4Cl and

extracted with ethyl acetate. The organic layer was dried with $MgSO_4$ and concentrated. The crude then underwent purification by flash column chromatography (30% EA/Pet. Ether 40-60 to 50% EA/Pet. Ether 40-60) to give a mixture of **32** and **33**. This mixture underwent further purification by flash column chromatography (5% EA/DCM to 10% EA/DCM) to give **32** as a colourless oil (1.7 mg, 4.1 μmol, 31%). R_f = 0.57 (50% EA/Pet. Ether 40-60). ^{1}H NMR (500 MHz, $CDCl_3$) δ 7.81 – 7.66 (m, 2H, **H22**, **H24**), 7.63 – 7.50 (m, 3H, **H23**, **H25**, **H26**), 5.53 (ddt, *J* = 17.2, 10.3, 6.9 Hz, 1H, **H4**), 5.23 (dd, *J* = 8.0, 1.5 Hz, 1H, **H9**), 5.00 (dq, *J* = 17.2, 1.6 Hz, 1H, **H3'**), 4.95 (ddt, *J* = 10.3, 2.1, 1.1 Hz, 1H, **H3**), 4.15 (q, *J* = 1.9, 1.3 Hz, 1H, **H7**), 3.63 (ddd, *J* = 8.4, 6.1, 2.1 Hz, 1H, **H6**), 3.44 (q, *J* = 6.5 Hz, 1H, **H13**), 2.57 (dtd, *J* = 10.1, 7.1, 1.5 Hz, 1H, **H12**), 2.12 (d, *J* = 15.1 Hz, 1H, **H8'**), 2.08 (dd, *J* = 10.1, 6.3 Hz, 1H, **H11'**), 2.02 – 1.98 (m, 1H, **H5'**), 1.97 – 1.91 (m, 1H, **H8**), 1.90 – 1.79 (m, 2H, **H14**), 1.60 (dt, *J* = 14.0, 6.8 Hz, 1H, **H5**), 1.16 (dd, *J* = 7.4, 6.3 Hz, 1H, **H11**), 1.05 (t, *J* = 7.3 Hz, 3H, **H15**). ^{13}C NMR (126 MHz, $CDCl_3$) δ 155.1 (**C16**), 134.1 (**C21**), 134.0 (**C4**), 131.1 (**C26**), 129.1 (**C23**, **C25**), 126.8 (**C22**, **C24**), 117.5 (**C3**), 85.7 (**C6**), 76.5 (**C9**), 75.5 (**C7**), 73.7 (**C13**), 50.0 (**C10**), 32.3 (**C5**), 31.8 (**C8**), 31.5 (**C12**), 30.3 (**C14**), 15.6 (**C11**), 10.1 (**C15**). IR (ν_{max} cm^{-1}): 2962-2852 (aliphatic sp^3 C–H stretchings, broad), 1641 (C=C stretching, weak), 1596 (tetrazole C=C and C=N stretchings, weak), 1497 (tetrazole C=N and N=N stretchings, strong), 1348 (sulfone SO_2 antisymmetric stretching, strong), 1180 (sulfone SO_2 symmetric stretching, strong), 1051 (ether C–O–C antisymmetric stretching, strong), 761 (monosubstituted benzene C–H out of plane deformation, strong). LRMS (ESI) $[M+H]^+$: *m/z* 417.2. HRMS (ESI) $[M+H]^+$: calculated for *m/z* 417.15910, found *m/z* 417.15919. ($C_{20}H_{25}O_4N_4{}^{32}S$). $[\alpha]_D^{25} = +107$ (c=0.10, $CHCl_3$). The structure of **32** was further confirmed by analysis of nOe correlations (see below).

(*S*)-1-((1*S*,2*S*,4*S*,7*S*)-2-Ethyl-7-((1-phenyl-1*H*-tetrazol-5-yl)sulfonyl)-3-oxabicyclo[5.1.0] oct-5-en-4-yl)but-3-en-1-ol 33

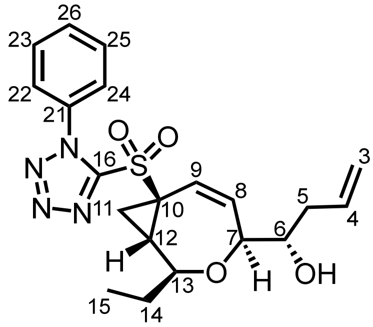

Compound **30** (6.5 mg, 13.0 μmol) was dissolved in dry THF (0.2 mL) and the reaction mixture was cooled to -78 °C. NaHMDS (0.14 mL, 0.1 M in THF, 14.0 μmol) was added at -78 °C where the solution turned from colourless to bright yellow. The reaction mixture was then gradually warmed to r.t. and the solution decolourised and became cloudy over 15 minutes. TLC analysis at this point indicated completion of reaction. The reaction mixture was quenched with sat. aq. NH_4Cl and extracted with ethyl acetate. The organic layer was dried with $MgSO_4$ and concentrated. The crude then underwent purification by flash column chromatography (30% EA/Pet. Ether 40-60 to 50% EA/Pet. Ether 40-60) to give a mixture of **32** and **33**. This mixture underwent further purification by flash column chromatography (5% EA/DCM to 10% EA/DCM) to give **33** as a colourless oil (3.2

mg, 7.7 μmol, 59%). R_f = 0.57 (50% EA/Pet. Ether 40-60). ^{1}H NMR (500 MHz, $CDCl_3$) δ 7.68 – 7.64 (m, 2H, **H22**, **H23**), 7.63 – 7.60 (m, 1H, **H26**), 7.59 – 7.54 (m, 2H, **H24**, **H25**), 6.08 (ddd, *J* = 11.8, 2.0, 1.3 Hz, 1H, **H9**), 5.80 (ddt, *J* = 17.3, 10.2, 7.1 Hz, 1H, **H4**), 5.62 (dd, *J* = 11.8, 2.6 Hz, 1H, **H8**), 5.14 – 5.07 (m, 2H, **H3**), 4.10 (dt, *J* = 3.1, 2.3 Hz, 1H, **H7**), 3.51 (qd, *J* = 6.4, 3.2 Hz, 1H, **H6**), 2.92 (ddd, *J* = 9.2, 7.4, 5.8 Hz, 1H, **H13**), 2.35 – 2.27 (m, 3H, **H5**, **H12**), 2.15 (d, *J* = 6.1 Hz, 1H, O**H**), 2.11 (dd, *J* = 9.5, 5.5 Hz, 1H, **H11'**), 1.72 – 1.65 (m, 2H, **H14**), 1.32 (dd, *J* = 6.9, 5.5 Hz, 1H, **H11**), 0.97 (t, *J* = 7.4 Hz, 3H, **H15**). ^{13}C NMR (126 MHz, $CDCl_3$) δ 153.1 (**C16**), 137.6 (**C8**), 134.5 (**C4**), 133.4 (**C21**), 131.5 (**C26**), 129.3 (**C24**, **C25**), 126.5 (**C22**, **C23**), 120.8 (**C9**), 118.1 (**C3**), 83.7 (**C7**), 81.3 (**C13**), 72.6 (**C6**), 44.3 (**C10**), 38.0 (**C5**), 32.9 (**C12**), 29.1 (**C14**), 22.7 (**C11**), 10.2 (**C15**). IR (ν_{max} cm^{-1}): 3563-3466 (O–H stretching, broad), 2964-2853 (aliphatic sp^3 C–H stretchings, broad), 1641 (C=C stretching, weak), 1595 (tetrazole C=C and C=N stretchings, weak), 1498 (tetrazole C=N and N=N stretchings, strong), 1348 (sulfone SO_2 antisymmetric stretching, strong), 1168 (sulfone SO_2 symmetric stretching, strong), 1049 (ether C–O–C antisymmetric stretching, strong), 761 (monosubstituted benzene C–H out of plane deformation, strong). LRMS (ESI) [M+H]$^+$: *m/z* 417.2. HRMS (ESI) [M+H]$^+$: calculated for *m/z* 417.15910, found *m/z* 417.15918. ($C_{20}H_{25}O_4N_4{}^{32}S$). $[\alpha]_D^{25} = +36.5$ (c=0.26, $CHCl_3$). The structure of **33** was further confirmed by analysis of nOe correlations (see below).

(1*S*,3*S*,4*R*,7*S*,9*S*)-9-Allyl-4-bromo-3-ethyl-2,8-dioxabicyclo[5.2.1]decan-6-one 37

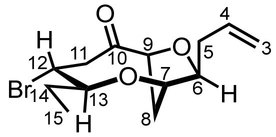

The compound **17** (10.0 mg, 33.0 μmol) was dissolved in CH_2Cl_2 (0.7 mL) at room temperature. Dess-Martin periodinane (17.0 mg, 39.0 μmol) was added and the reaction mixture stirring was continued for 1 hour. TLC analysis at this stage indicated the complete consumption of starting material. The reaction mixture was quenched with sat. aq. $NaHCO_3$ and sat. aq. $Na_2S_2O_3$. The aqueous layer was extracted with CH_2Cl_2 and the organic layers were combined and dried with $MgSO_4$. The crude was concentrated and underwent purification by flash column chromatography (50% EA/Pet. Ether 40-60) to give the compound **37** as a colourless oil (8.5 mg, 28.0 μmol, 85%). R_f = 0.57 (10% EA/CH_2Cl_2). ^{1}H NMR (500 MHz, $CDCl_3$) δ 5.78 (ddt, *J* = 17.2, 10.2, 7.0 Hz, 1H, **H4**), 5.14 (dq, *J* = 17.2, 1.6 Hz, 1H, **H3'**), 5.07 (ddt, *J* = 10.2, 2.1, 1.1 Hz, 1H, **H3**), 4.26 (d, *J* = 7.1 Hz, 1H, **H9**), 4.19 (q, *J* = 1.9 Hz, 1H, **H7**), 3.98 (ddd, *J* = 11.4, 10.3, 5.2 Hz, 1H, **H12**), 3.92 (ddd, *J* = 8.7, 6.3, 2.4 Hz, 1H, **H6**), 3.58 (ddd, *J* = 10.7, 8.4, 2.6 Hz, 1H, **H13**), 3.00 (t, *J* = 11.5 Hz, 1H, **H11'**), 2.95 – 2.85 (m, 2H, **H8'**, **H11**), 2.53 – 2.44 (m, 1H, **H5'**), 2.40 (dddt, *J* = 13.8, 8.6, 7.4, 1.2 Hz, 1H, **H5**), 2.15 – 2.06 (m, 2H, **H8**, **H14'**), 1.55 – 1.48 (m, 1H, **H14**), 0.96 (t, *J* = 7.4 Hz, 3H, **H15**). ^{13}C NMR (126 MHz, $CDCl_3$) δ 206.5 (**C10**), 134.2 (**C4**), 117.7 (**C3**), 83.2 (**C6**), 81.2 (**C13**), 79.9 (**C9**), 77.1 (**C7**), 51.7 (**C12**), 46.8 (**C11**), 34.6 (**C8**), 33.9 (**C5**), 27.0 (**C14**), 9.6 (**C15**). IR (ν_{max} cm^{-1}): 2963-2921 (aliphatic sp^3 C–H stretchings, broad), 1713 (ketone C=O stretching, strong), 1641 (C=C stretching, medium), 1080 (ether C–O–C antisymmetric stretching, strong). LRMS (ESI) [M+Na]$^+$: *m/z* 325.0 and 327.0. HRMS (ESI) [M+Na]$^+$: calculated for *m/z* 325.04098 and 327.03893, found *m/z* 325.04102 and

327.03891 ($C_{13}H_{19}O_3{}^{79}BrNa$ and $C_{13}H_{19}O_3{}^{81}BrNa$). $[\alpha]_D^{25} = +10.0$ (c=0.30, $CHCl_3$).

(1*S*,2*S*,4*R*,5*S*,7*S*,8*S*)-8-Allyl-5-ethyl-6,9-dioxatricyclo[5.2.1.0^{2,4}]decane 39 and (*S*)-1-((2*S*,4*Z*,6*Z*,8*S*)-8-ethyl-3,8-dihydro-2*H*-oxocin-2-yl)but-3-en-1-ol 40

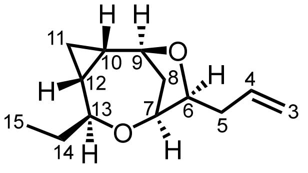

A 39

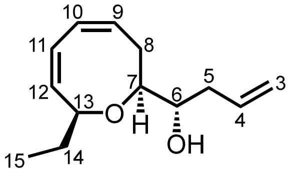

B 40

The compound **37** (6.0 mg, 20.0 μmol) was dissolved in EtOH (0.4 mL) and a solution of $N_2H_4{\cdot}H_2O$ in EtOH (1.0 M, 51.0 μL, 51.0 μmol) was added. The reaction mixture was stirred at room temperature till reaction completion as suggested by TLC analysis. All volatiles were removed under reduced pressure to yield the crude hydrazone. In parallel with the preparation of the hydrazone, DMSO (4.0 mL) was mixed with NaH (27.0 mg, 0.7 mmol) and heated to 60 °C for 3 hours. The resulting dimsyl anion solution was cooled to room temperature. The crude hydrazone was then mixed with the dimsyl anion solution (0.44 mL) at room temperature and stirring was continued for 1 hour, in which effervescence was observed upon addition of the dimsyl anion solution. TLC analysis at this stage indicated the complete consumption of starting materials. The reaction mixture was quenched with sat. aq. NH_4Cl and extracted with ethyl acetate. The organic layers were combined and dried with $MgSO_4$. The crude was concentrated and underwent purification by flash column chromatography (15% EA/Pet. Ether 40-60) to give an inseparable mixture of compounds **39** and **40** as a colourless oil (2.5 mg, 12.0 μmol, 60%, **39**:**40** = 4.5:1). R_f = 0.69 (30% EA/Pet. Ether 40-60). NMR characterisation for **39**: ^{1}H NMR (500 MHz, $CDCl_3$) δ 5.96 – 5.85 (m, 1H, **H4A**), 5.15 (d, *J* = 17.2 Hz, 1H, **H3A'**), 5.05 (d, *J* = 10.2 Hz, 1H, **H3A**), 4.73 (d, *J* = 7.4 Hz, 1H, **H9A**), 4.25 (t, *J* = 3.8 Hz, 1H, **H7A**), 3.82 (td, *J* = 7.0, 3.3 Hz, 1H, **H6A**), 3.35 (q, *J* = 6.7 Hz, 1H, **H13A**), 2.60 – 2.53 (m, 1H, **H5A'**), 2.47 (dt, *J* = 14.5, 7.6 Hz, 1H, **H5A**), 2.05 (d, *J* = 14.5 Hz, 1H, **H8A'**), 1.75 – 1.61 (m, 3H, **H8A**, **H14A**), 1.11 (q, *J* = 8.4 Hz, 1H, **H10A**), 0.95 (m, 4H, **H12A**, **H15A**), 0.78 (td, *J* = 8.6, 5.1 Hz, 1H, **H11A'**), 0.24 (q, *J* = 5.5 Hz, 1H, **H11A**). ^{13}C NMR (126 MHz, $CDCl_3$) δ 136.2 (**C4A**), 116.5 (**C3A**), 84.7 (**C6A**), 78.8 (**C9A**), 76.8 (**C7A**), 76.4 (**C13A**), 35.6 (**C5A**), 30.4 (**C14A**), 30.2 (**C8A**), 22.8 (**C12A**), 20.9 (**C10A**), 10.4 (**C15A**), 9.0 (**C11A**). NMR characterisation for **40**: ^{1}H NMR (500 MHz, $CDCl_3$) δ 6.10 (dd, *J* = 11.1, 3.4 Hz, 1H, **H10B**), 6.02 – 5.96 (m, 1H, **H11B**), 5.96 – 5.85 (m, 1H, **H4B**), 5.79 (q, *J* = 8.9 Hz, 1H, **H9B**), 5.51 (dd, *J* = 11.1, 6.9 Hz, 1H, **H12B**), 5.15 (d, *J* = 17.2 Hz, 1H, **H3B'**), 5.10 (d, *J* = 11.3 Hz, 1H, **H3B**), 3.56 (p, *J* = 5.4, 4.7 Hz, 1H, **H6B**), 3.50 (q, *J* = 6.4 Hz, 1H, **H13B**), 3.01 (t, *J* = 6.4 Hz, 1H, **H7B**), 2.60 – 2.53 (m, 2H, O**H**, **H8B'**), 2.47 (dt, *J* = 14.5, 7.6 Hz, 1H, **H5B'**), 2.32 (dt, *J* = 14.3, 7.4 Hz, 1H, **H5B**), 1.95 (dt, *J* = 14.4, 7.4 Hz, 1H, **H8B**), 1.75 – 1.61 (m, 2H, **H14B**), 0.95 (m, 3H, **H15B**). ^{13}C NMR (126 MHz, $CDCl_3$) δ 136.2 (**C4B**), 133.1 (**C12B**), 129.4 (**C9B**), 129.0 (**C10B**), 127.5 (**C11B**), 117.4 (**C3B**), 78.2 (**C13B**), 76.7 (**C7B**), 74.1 (**C6B**), 38.3 (**C5B**), 32.6 (**C8B**), 30.3 (**C14B**), 9.0 (**C15B**). LRMS (ESI) $[M+Na]^+$: *m/z* 231.1. HRMS (ESI) $[M+Na]^+$: calculated for *m/z* 231.13555, found *m/z* 231.13576 ($C_{13}H_{20}O_2Na$). The structure of **39** was further assigned by analysis of nOe correlations (see below).

(*S*)-1-((2*S*,7*R*,8*S*,*Z*)-7-Bromo-8-ethyl-3,6,7,8-tetrahydro-2*H*-oxocin-2-yl)but-3-en-1-ol 41

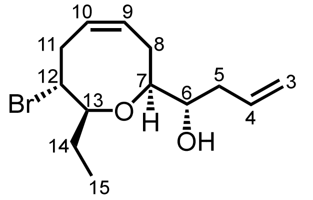

The compound **37** (10.5 mg, 35.0 μmol) was dissolved in EtOH (0.7 mL) at room temperature. A solution of $N_2H_4{\cdot}H_2O$ in EtOH (1.0 M, 90.0 μL, 90.0 μmol) was added. The reaction mixture was stirred at room temperature till reaction completion as suggested by TLC analysis. All volatiles were removed under reduced pressure to yield the crude hydrazone. The crude hydrazone was dissolved in THF (1.0 mL) and the reaction mixture was cooled to -78 °C, a solution of NaHMDS in THF (0.1 M, 0.39 mL, 39.0 μmol) was added and gradually warmed to room temperature. Effervescence was observed during the warming of the reaction mixture. TLC analysis at this stage indicated the complete consumption of starting materials. The reaction mixture was quenched with sat. aq. NH_4Cl and extracted with ethyl acetate. The organic layers were combined and dried with $MgSO_4$. The crude was concentrated and underwent purification by flash column chromatography (3% EA/CH_2Cl_2 to 5% EA/CH_2Cl_2) to give the compound **41** as a colourless oil (4.2 mg, 14.5 μmol, 42%), with trace **40** that was discarded after column chromatography. R_f = 0.60 (5% EA/CH_2Cl_2). ^{1}H NMR (700 MHz, $CDCl_3$) δ 6.05 – 5.93 (m, 1H, **H9**), 5.93 – 5.84 (m, 2H, **H4**, **H10**), 5.24 – 5.02 (m, 2H, **H3**), 4.09 (dt, *J* = 10.2, 3.4 Hz, 1H, **H12**), 3.57 – 3.54 (m, 1H, **H6**), 3.54 – 3.51 (m, 1H, **H13**), 3.24 (dd, *J* = 10.6, 5.6 Hz, 1H, **H7**), 3.17 (ddd, *J* = 13.9, 9.3, 3.5 Hz, 1H, **H11'**), 2.48 (ddd, *J* = 14.0, 6.2, 3.3 Hz, 1H, **H11**), 2.46 – 2.41 (m, 2H, O**H**, **H8'**), 2.41 – 2.36 (m, 1H, **H5'**), 2.24 (dtd, *J* = 14.2, 7.8, 1.3 Hz, 1H, **H5**), 2.14 (dd, *J* = 14.3, 8.4 Hz, 1H, **H8**), 1.98 (dqd, *J* = 14.9, 7.4, 2.5 Hz, 1H, **H14'**), 1.64 (dp, *J* = 14.6, 7.4 Hz, 1H, **H14**), 0.98 (t, *J* = 7.4 Hz, 3H, **H15**). ^{13}C NMR (176 MHz, $CDCl_3$) δ 134.8 (**C4**), 129.8 (**C9**), 128.8 (**C10**), 117.7 (**C3**), 83.9 (**C7**), 83.8 (**C13**), 73.6 (**C6**), 55.9 (**C12**), 38.2 (**C5**), 32.5 (**C11**), 30.8 (**C8**), 25.8 (**C14**), 9.3 (**C15**). IR (ν_{max} cm^{-1}): 3446 (O–H stretching, broad), 2962-2923 (aliphatic sp^3 C–H stretchings, broad), 1641 (C=C stretching, medium), 1064 (ether C–O–C antisymmetric stretching, strong). LRMS (ESI) [M–Br+Na]$^+$: *m/z* 231.1. HRMS (ESI) [M–Br+Na]$^+$: calculated for *m/z* 231.13555, found *m/z* 231.13573 ($C_{13}H_{20}O_2Na$). $[\alpha]_D^{25} = -11.2$ (c=0.42, $CHCl_3$).

(1*S*,3*S*,4*R*,7*S*,9*S*)-4-Bromo-3-ethyl-9-((E)-pent-2-en-4-yn-1-yl)-2,8-dioxabicyclo [5.2.1]decan-6-one 42

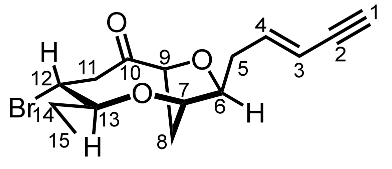

The compound *ent*-(*E*)-laurefucin **18** (10.0 mg, 30.3 μmol) was dissolved in CH_2Cl_2 (2.0 mL) at room temperature. Dess-Martin periodinane (15.0 mg, 35.3 μmol) was added and stirred at room temperature until completion. TLC analysis at this stage indicated complete consumption of starting materials. The reaction mixture was then quenched with sat. aq. $NaHCO_3$ and sat. aq. $Na_2S_2O_3$. The

aqueous layer was extracted with CH_2Cl_2 and the organic layers were combined and dried with $MgSO_4$. The crude was concentrated and underwent purification by flash column chromatography (30% EA/CH_2Cl_2) to give the compound **42** as a colourless oil (9.9 mg, 30.3 μmol, 100%). R_f = 0.71 (50% EA/CH_2Cl_2). ^{1}H NMR (500 MHz, $CDCl_3$) δ 6.18 (dt, *J* = 15.3, 7.4 Hz, 1H, **H4**), 5.57 (dd, *J* = 15.3, 1.2 Hz, 1H, **H3**), 4.26 (d, *J* = 7.1 Hz, 1H, **H9**), 4.19 (s, 1H, **H7**), 3.97 (td, *J* = 11.0, 5.3 Hz, 1H, **H12**), 3.92 (ddd, *J* = 8.7, 6.3, 2.4 Hz, 1H, **H6**), 3.59 (ddd, *J* = 10.7, 8.9, 2.5 Hz, 1H, **H13**), 3.01 (t, *J* = 11.6 Hz, 1H, **H11'**), 2.96 – 2.91 (m, 1H, **H8'**), 2.91 – 2.85 (m, 1H, **H11**), 2.80 (d, *J* = 2.2 Hz, 1H, **H1**), 2.54 (dt, *J* = 13.0, 6.3 Hz, 1H, **H5'**), 2.45 (dt, *J* = 14.3, 8.1 Hz, 1H, **H5**), 2.16 – 2.07 (m, 2H, **H8**, **H14'**), 1.53 – 1.49 (m, 1H, **H14**), 0.97 (t, *J* = 7.4 Hz, 3H, **H15**). ^{13}C NMR (126 MHz, $CDCl_3$) δ 206.3 (**C10**), 141.5 (**C4**), 111.7 (**C3**), 82.4 (**C6**), 82.2 (**C2**), 81.4 (**C13**), 80.0 (**C9**), 77.1 (**C7**), 76.6 (**C1**), 51.5 (**C12**), 46.8 (**C11**), 34.6 (**C8**), 33.2 (**C5**), 27.0 (**C14**), 9.6 (**C15**). IR (ν_{max} cm^{-1}): 3290 (acetylene C–H stretching, medium), 2920-2853 (aliphatic sp^3 C–H stretchings, broad), 2101 (C≡C stretching, weak), 1713 (ketone C=O stretching, strong), 1632 (C=C stretching, medium), 1061 (ether C–O–C antisymmetric stretching, strong). HRMS (APCI) [M+H]$^+$: calculated for *m/z* 327.05903 and 329.05699, found *m/z* 327.05922 and 329.05725 ($C_{15}H_{21}O_2$). $[\alpha]_D^{25} = +22.0$ (c=0.15, $CHCl_3$).

(*S*,*E*)-1-((2*S*,4*Z*,6*Z*,8*S*)-8-Ethyl-3,8-dihydro-2*H*-oxocin-2-yl)hex-3-en-5-yn-1-ol 43

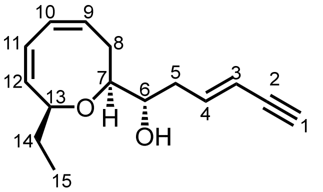

The compound **42** (9.9 mg, 30.3 μmol) was dissolved in EtOH (0.55 mL) at room temperature. A solution of $N_2H_4 \cdot H_2O$ in EtOH (1.0 M, 80.0 μL, 80.0 μmol) was added. The reaction mixture was stirred at room temperature till reaction completion as suggested by TLC analysis. All volatiles were removed under reduced pressure to yield the crude hydrazone. The crude hydrazone was dissolved in THF (1.0 mL) and the reaction mixture was cooled to -78 °C, a solution of NaHMDS in THF (0.1 M, 0.66 mL, 66.0 μmol) was added and gradually warmed to room temperature. Effervescence was observed during the warming of the reaction mixture. TLC analysis at this stage indicated the complete consumption of starting materials. The reaction mixture was quenched with sat. aq. NH_4Cl and extracted with ethyl acetate. The organic layers were combined and dried with $MgSO_4$. The crude was concentrated and underwent purification by flash column chromatography (1% EA/CH_2Cl_2 to 2% EA/CH_2Cl_2) to give a separable mixture of the compound **23** (3.0 mg, 9.6 μmol, 32%) and **43** (0.8 mg, 3.4 μmol, 11%) as a colourless oils. R_f for **43** = 0.54 (3% EA/CH_2Cl_2). ^{1}H NMR (500 MHz, $CDCl_3$) δ 6.34 (dt, *J* = 16.2, 7.2 Hz, 1H, **H4**), 6.10 (dd, *J* = 10.8, 3.8 Hz, 1H, **H10**), 5.99 (dd, *J* = 11.2, 3.5 Hz, 1H, **H11**), 5.79 (dt, *J* = 10.8, 8.3 Hz, 1H, **H9**), 5.57 (dq, *J* = 16.0, 1.8 Hz, 1H, **H3**), 5.51 (dd, *J* = 11.1, 6.9 Hz, 1H, **H12**), 3.57 (dq, *J* = 7.4, 5.0 Hz, 1H, **H6**), 3.54 – 3.46 (m, 1H, **H13**), 2.99 (ddd, *J* = 7.1, 5.5, 1.8 Hz, 1H, **H7**), 2.82 (d, *J* = 2.3 Hz, 1H, **H1**), 2.52 (d, *J* = 5.0 Hz, 1H, O**H**), 2.52 – 2.49 (m, 1H, **H8'**), 2.49 – 2.46 (m, 1H, **H5'**), 2.39 (dtd, *J* = 14.7, 7.4, 1.4 Hz, 1H, **H5**), 1.94 (dt, *J* = 14.4, 7.7 Hz, 1H, **H8**), 1.68 (dq, *J* = 15.1, 7.5 Hz, 1H, **H14'**), 1.63 – 1.58 (m, 1H, **H14**), 0.93 (t, *J* = 7.4 Hz, 3H, **H15**). ^{13}C NMR (126 MHz, $CDCl_3$) δ 142.7 (**C4**), 133.0 (**C12**), 129.3 (**C9**), 129.2

(**C10**), 127.6 (**C11**), 111.1 (**C3**), 82.4 (**C2**), 78.1 (**C13**), 76.6 (**C7**), 76.4 (**C1**), 74.0 (**C6**), 37.6 (**C5**), 32.7 (**C8**), 30.2 (**C14**), 10.3 (**C15**). IR (ν_{max} cm^{-1}): 3454 (O–H stretching , broad), 3310 (acetylene C–H stretching, medium), 2960-2853 (aliphatic sp^3 C–H stretchings, broad), 2103 (C≡C stretching, weak), 1667 (C=C stretching, medium), 1063 (ether C–O–C antisymmetric stretching, strong). LRMS (ESI) $[M+H]^+$: *m/z* 233.2. HRMS (ESI) $[M+H]^+$: calculated for *m/z* 233.15361, found *m/z* 233.15371 ($C_{15}H_{21}O_2$). $[\alpha]_D^{25} = +56.3$ (c=0.08, $CHCl_3$).

(*S*,*E*)-1-((2*S*,7*R*,8*S*,*Z*)-7-Bromo-8-ethyl-3,6,7,8-tetrahydro-2*H*-oxocin-2-yl)hex-3-en-5-yn-1-ol *ent*-deacetyllaurencin 23

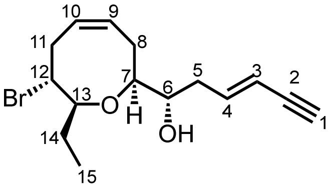

The compound **42** (9.9 mg, 30.3 μmol) was dissolved in EtOH (0.55 mL) at room temperature. A solution of $N_2H_4 \cdot H_2O$ in EtOH (1.0 M, 80.0 μL, 80.0 μmol) was added. The reaction mixture was stirred at room temperature till reaction completion as suggested by TLC analysis. All volatiles were removed under reduced pressure to yield the crude hydrazone. The crude hydrazone was dissolved in THF (1.0 mL) and the reaction mixture was cooled to -78 °C, a solution of NaHMDS in THF (0.1 M, 0.66 mL, 66.0 μmol) was added and gradually warmed to room temperature. Effervescence was observed during the warming of the reaction mixture. TLC analysis at this stage indicated the complete consumption of starting materials. The reaction mixture was quenched with sat. aq. NH_4Cl and extracted with ethyl acetate. The organic layers were combined and dried with $MgSO_4$. The crude was concentrated and underwent purification by flash column chromatography (1% EA/CH_2Cl_2 to 2% EA/CH_2Cl_2) to give a separable mixture of the compound **23** (3.0 mg, 9.6 μmol, 32%) and **43** (0.8 mg, 3.4 μmol, 11%) as a colourless oils. R_f for **23** = 0.71 (3% EA/CH_2Cl_2). ^{1}H NMR (500 MHz, $CDCl_3$) δ 6.31 (dt, *J* = 15.2, 7.2 Hz, 1H, **H4**), 5.96 (dt, *J* = 10.6, 7.7 Hz, 1H, **H9**), 5.93 – 5.84 (m, 1H, **H10**), 5.57 (dq, *J* = 16.0, 1.8 Hz, 1H, **H3**), 4.08 (dt, *J* = 10.0, 3.5 Hz, 1H, **H12**), 3.58 – 3.54 (m, 1H, **H6**), 3.54 – 3.50 (m, 1H, **H13**), 3.22 (ddd, *J* = 10.5, 5.8, 1.3 Hz, 1H, **H7**), 3.15 (ddd, *J* = 13.1, 9.0, 3.6 Hz, 1H, **H11'**), 2.83 (d, *J* = 2.3 Hz, 1H, **H1**), 2.48 (ddd, *J* = 14.0, 6.0, 3.2 Hz, 1H, **H11**), 2.43 (d, *J* = 4.5 Hz, 1H, O**H**), 2.45 – 2.38 (m, 2H, **H5'**, **H8'**), 2.35 – 2.26 (m, 1H, **H5**), 2.11 (ddd, *J* = 14.3, 8.2, 1.4 Hz, 1H, **H8**), 1.97 (dqd, *J* = 14.9, 7.5, 2.8 Hz, 1H, **H14'**), 1.64 (dp, *J* = 14.4, 7.2 Hz, 1H, **H14**), 0.97 (t, *J* = 7.4 Hz, 3H, **H15**). ^{13}C NMR (126 MHz, $CDCl_3$) δ 142.3 (**C4**), 129.6 (**C9**), 128.9 (**C10**), 111.4 (**C3**), 83.8 (**C13**), 83.7 (**C7**), 82.2 (**C2**), 76.6 (**C1**), 73.5 (**C6**), 55.6 (**C12**), 37.4 (**C5**), 32.5 (**C11**), 30.8 (**C8**), 25.8 (**C14**), 9.2 (**C15**). ^{1}H NMR (500 MHz, C_6D_6) δ 6.33 (dt, *J* = 16.1, 7.2 Hz, 1H, **H4**), 5.85 (ddd, *J* = 10.6, 9.4, 6.2 Hz, 1H, **H10**), 5.63 (dt, *J* = 10.6, 7.8 Hz, 1H, **H9**), 5.45 (dq, *J* = 16.0, 1.8 Hz, 1H, **H3**), 3.75 (dt, *J* = 10.0, 3.4 Hz, 1H, **H12**), 3.31 (ddd, *J* = 9.8, 6.9, 2.7 Hz, 1H, **H13**), 3.16 (dq, *J* = 9.2, 4.8 Hz, 1H, **H6**), 2.85 (ddd, *J* = 13.5, 9.4, 3.6 Hz, 1H, **H11'**), 2.74 (ddd, *J* = 10.6, 5.3, 1.4 Hz, 1H, **H7**), 2.57 (d, *J* = 2.3 Hz, 1H, **H1**), 2.24 (ddd, *J* = 14.0, 6.2, 3.2 Hz, 1H, **H11**), 2.12 – 2.04 (m, 1H, **H8'**), 2.01 (dddt, *J* = 14.8, 7.1, 4.0, 1.8 Hz, 1H, **H5'**), 1.92 (dtd, *J* = 14.7, 7.7, 1.6 Hz, 1H, **H5**), 1.86 (d, *J* = 5.0 Hz, 1H, O**H**), 1.81 (dqd, *J* = 14.9, 7.4, 2.7 Hz, 1H, **H14'**), 1.64 (ddd, *J*

= 14.3, 8.5, 1.3 Hz, 1H, **H8**), 1.42 (dqd, *J* = 14.9, 7.4, 6.9 Hz, 1H, **H14**), 0.77 (t, *J* = 7.4 Hz, 3H, **H15**). ^{13}C NMR (126 MHz, C_6D_6) δ 142.8 (**C4**), 129.9 (**C9**), 128.6 (**C10**), 111.5 (**C3**), 83.7 (**C13**), 83.6 (**C7**), 82.5 (**C2**), 77.1 (**C1**), 73.3 (**C6**), 56.1 (**C12**), 37.2 (**C5**), 32.5 (**C11**), 30.4 (**C8**), 26.0 (**C14**), 9.1 (**C15**). IR (ν_{max} cm^{-1}): 3441 (O–H stretching , broad), 3293 (acetylene C–H stretching, medium), 2922 (aliphatic sp^3 C–H stretchings, broad), 2103 (C≡C stretching, weak), 1668 (C=C stretching, medium), 1059 (ether C–O–C antisymmetric stretching, strong). HRMS (APCI) [M+H]$^+$: calculated for *m/z* 313.08087 and 315.07882, found *m/z* 313.07988 and 315.07777 ($C_{15}H_{22}O_2{}^{79}Br$ and $C_{15}H_{22}O_2{}^{81}Br$). $[\alpha]_D^{25} = -34.7$ (c=0.05, $CHCl_3$). Literature value of enantiomer: $[\alpha]_D^{17} = +46.1$ ($c = 1.15$, $CHCl_3$).[10] Spectroscopic data are in accordance with literature data.[10,11]

(*S*,*E*)-1-((2*S*,7*R*,8*S*,*Z*)-7-Bromo-8-ethyl-3,6,7,8-tetrahydro-2*H*-oxocin-2-yl)hex-3-en-5-yn-1-yl acetate *ent*-laurencin 24

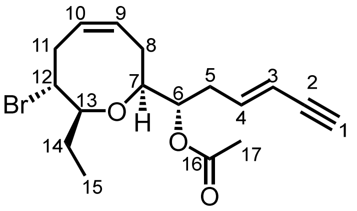

The compound **23** (2.5 mg, 8.0 μmol) was dissolved in CH_2Cl_2 (0.8 mL) at room temperature and then DMAP (3.0 mg, 25.0 μmol) was added followed by a solution of Ac_2O in CH_2Cl_2 (0.1 M, 0.25 mL, 25.0 μmol). The reaction mixture was stirred at room temperature for 1 hour, in which TLC analysis at this stage indicated the completion consumption of starting materials. The reaction mixture was quenched with sat. aq. $NaHCO_3$ and extracted with CH_2Cl_2. The organic layers were combined and dried with $MgSO_4$. The crude was concentrated and underwent purification by flash column chromatography (5% EA/Pet. Ether 40-60 to 10% EA/Pet. Ether 40-60) to give the compound **24** as a colourless oil (2.6 mg, 7.3 μmol, 91%). R_f = 0.42 (10% EA/Pet. Ether 40-60). ^{1}H NMR (500 MHz, $CDCl_3$) δ 6.15 (dt, *J* = 15.2, 7.3 Hz, 1H, **H4**), 5.97 – 5.91 (m, 1H, **H9**), 5.91 – 5.85 (m, 1H, **H10**), 5.53 (dq, *J* = 15.9, 1.7 Hz, 1H, **H3**), 5.00 (dt, *J* = 8.7, 4.3 Hz, 1H, **H6**), 4.07 (dt, *J* = 10.0, 3.4 Hz, 1H, **H12**), 3.43 (ddd, *J* = 9.8, 7.2, 2.6 Hz, 1H, **H13**), 3.39 (dd, *J* = 10.6, 4.6 Hz, 1H, **H7**), 3.15 (ddd, *J* = 13.8, 8.5, 3.6 Hz, 1H, **H11'**), 2.82 (d, *J* = 2.2 Hz, 1H, **H1**), 2.55 – 2.49 (m, 1H, **H5'**), 2.49 – 2.44 (m, 1H, **H11**), 2.44 – 2.39 (m, 1H, **H5**), 2.39 – 2.30 (m, 1H, **H8'**), 2.12 – 2.03 (m, 4H, **H8**, **H17**), 1.95 (dqd, *J* = 14.8, 7.5, 2.6 Hz, 1H, **H14'**), 1.59 (m, 1H, **H14**), 0.98 (t, *J* = 7.4 Hz, 3H, **H15**). ^{13}C NMR (126 MHz, $CDCl_3$) δ 170.5 (**C16**), 141.3 (**C4**), 129.4 (**C9**), 129.1 (**C10**), 111.8 (**C3**), 84.7 (**C13**), 82.0 (**C2**), 81.5 (**C7**), 77.0 (**C1**), 74.2 (**C6**), 56.1 (**C12**), 34.0 (**C5**), 32.4 (**C11**), 29.8 (**C8**), 25.9 (**C14**), 21.2 (**C17**), 9.5 (**C15**). IR (ν_{max} cm^{-1}): 3291 (acetylene C–H stretching, medium), 2961-2922 (aliphatic sp^3 C–H stretchings, broad), 1739 (ester C=O stretching, strong), 1632 (C=C stretching, medium), 1071 (ether C–O–C antisymmetric stretching, strong). LRMS (ESI) [M+H]$^+$: *m/z* 355.1 and 357.1. HRMS (ESI) [M+H]$^+$: calculated for *m/z* 355.09033 and 357.08829, found *m/z* 355.09027 and 357.08815 ($C_{17}H_{24}O_3{}^{79}Br$ and $C_{17}H_{24}O_3{}^{81}Br$). $[\alpha]_D^{25} = -61.0$ (c=0.10, $CHCl_3$). Literature values of enantiomer (i.e the natural enantiomer of laurencin):

$[\alpha]_D^{20} = +70.0$ ($c = 0.05$, $CHCl_3$) ,[12] $[\alpha]_D^{25} = +69.0$ (c=1.00, $CHCl_3$) ,[13] $[\alpha]_D^{24} = +68.2$ (c=0.35,

CHCl$_3$) ,[14] $[\alpha]_D^{25}$ = +72.5 (c=0.17, CHCl$_3$) ,[15] $[\alpha]_D^{27}$ = +70.2 (c=1.00, CHCl$_3$) ,[10] $[\alpha]_D^{24}$ = +69.6 (c=0.26, CHCl$_3$),[16] $[\alpha]_D^{24}$ = +51.7 (CHCl$_3$) in this case, the low value of the specific rotation was attributed to minor contamination by the *cis* enyne diastereomer of laurencin.[17] Spectroscopic data are in accordance with literature data.[10,12–22]

***N'*-((1*S*,3*S*,4*R*,6*S*,7*S*,9*S*)-9-allyl-4-bromo-3-ethyl-2,8-dioxabicyclo[5.2.1]decan-6-yl)-4-methylbenzenesulfonohydrazide S6**

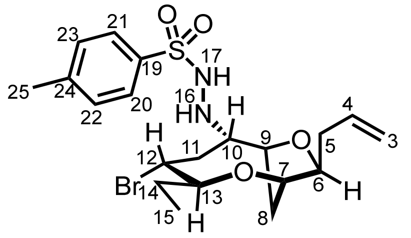

The compound **14** (25.0 mg, 65.0 µmol) was dissolved in CH_2Cl_2 (6.5 mL) and the reaction mixture was cooled to -40 °C. A solution of $TiCl_4$ in CH_2Cl_2 (0.5 M, 0.26 mL, 0.13 mmol) was then added, quickly followed by AgAl(pftb)$_4$·CH_2Cl_2 (226.0 mg, 0.20 mmol) in CH_2Cl_2 (1.0 mL) and stirred at -40 °C for 2 hours. The reaction mixture was then cooled to -78 °C, TsNHNH$_2$ (121.0 mg, 0.65 mmol) was added with AgBF$_4$ (128.0 mg, 0.65 mmol) and stirred at this temperature for 1 hour. TLC analysis at this stage indicated complete consumption of starting material. The reaction mixture was quenched with sat. aq. NaHCO$_3$ and excess TBAI. The aqueous layer was extracted with CH_2Cl_2, and all the organic layers were combined and dried with MgSO$_4$. The crude was concentrated and underwent purification by flash column chromatography (30% EA/Pet. Ether 40-60 to 50% EA/Pet. Ether 40-60) to give the compound **S6** as a white opaque oil (17.0 mg, 36.0 µmol, 55%). R_f = 0.34 (50% EA/Pet. Ether 40-60). ^{1}H NMR (500 MHz, CDCl$_3$) δ 7.83 – 7.73 (m, 2H, **H20**, **H21**), 7.40 – 7.29 (m, 2H, **H22**, **H23**), 5.97 (s, 1H, **NH17**), 5.79 (ddt, *J* = 17.2, 10.2, 7.0 Hz, 1H, **H4**), 5.14 (dq, *J* = 17.2, 1.6 Hz, 1H, **H3'**), 5.08 (ddt, *J* = 10.2, 2.2, 1.1 Hz, 1H, **H3**), 3.97 (td, *J* = 2.4, 1.2 Hz, 1H, **H7**), 3.88 – 3.82 (m, 1H, **H9**), 3.79 (td, *J* = 7.3, 2.5 Hz, 1H, **H6**), 3.41 (ddd, *J* = 10.8, 8.5, 2.5 Hz, 1H, **H13**), 3.32 (s, 1H, **H12**), 2.90 (dd, *J* = 9.5, 4.7 Hz, 1H, **H10**), 2.44 (s, 3H, **H25**), 2.41 (d, *J* = 14.5 Hz, 1H, **H8'**), 2.34 (tt, *J* = 7.1, 1.3 Hz, 2H, **H5**), 2.22 (dd, *J* = 15.0, 4.7 Hz, 1H, **H11'**), 2.05 (dqd, *J* = 14.7, 7.4, 2.3 Hz, 1H, **H14'**), 1.85 – 1.76 (m, 2H, **H8**, **H11**), 1.38 (ddq, *J* = 14.7, 8.5, 7.3 Hz, 1H, **H14**), 0.89 (t, *J* = 7.3 Hz, 3H, **H15**). ^{13}C NMR (126 MHz, CDCl$_3$) δ 144.3 (**C24**), 135.5 (**C19**), 134.6 (**C4**), 129.8 (**C22**, **C23**), 128.5 (**C20**, **C21**), 117.4 (**C3**), 83.5 (**C6**), 80.7 (**C9**), 80.5 (**C13**), 76.9 (**C7**), 67.3 (**C10**), 53.1 (**C12**), 39.2 (**C11**), 34.5 (**C5**), 33.9 (**C8**), 28.3 (**C14**), 21.7 (**C25**), 9.6 (**C15**). IR (ν_{max} cm^{-1}): 3252 (N–H stretching, broad), 2961-2855 (aliphatic sp^3 C–H stretchings, broad), 1663 (N–H distortion, medium), 1642 (C=C stretching, medium), 1328 (SO$_2$ antisymmetric stretching, strong), 1160 (SO$_2$ symmetric stretching, strong), 1092 (ether C–O–C antisymmetric stretching, strong). LRMS (ESI) [M+Na]$^+$: *m/z* 495.1 and 497.1. HRMS (ESI) [M+Na]$^+$: calculated for *m/z* 495.09236 and 497.09032, found *m/z* 495.09231 and 497.09014 ($C_{20}H_{29}O_4N_2{}^{79}BrNa^{32}S$ and $C_{20}H_{29}O_4N_2{}^{81}BrNa^{32}S$). $[\alpha]_D^{25}$ = +37.2 (c=0.10, CHCl$_3$).

4) Structure determination – Mosher ester analysis and assignment of relative configurations

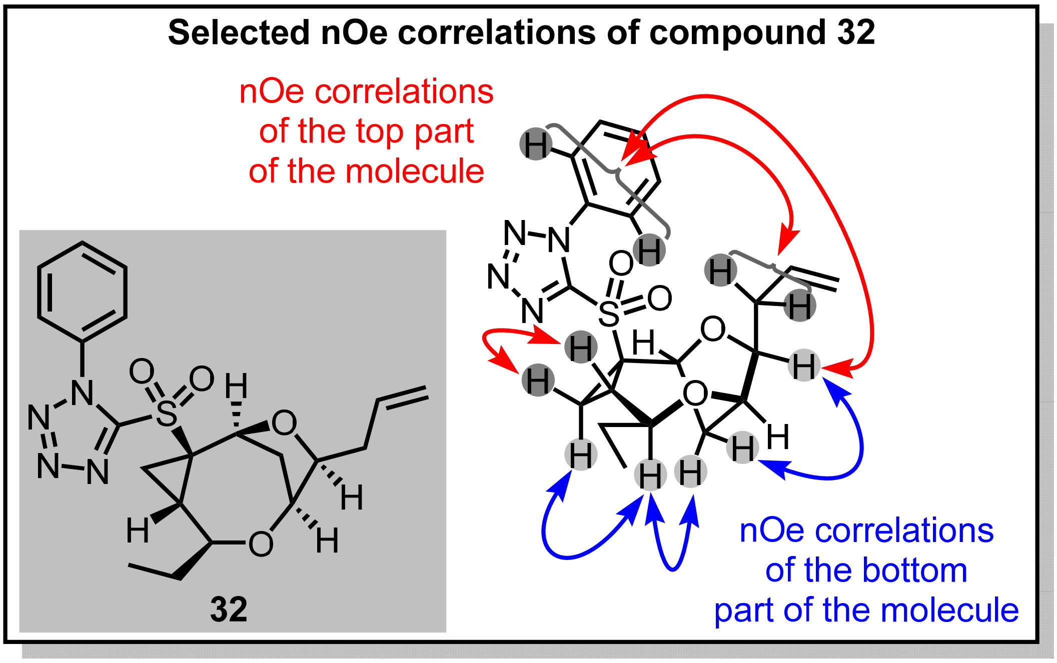

Figure S1. Assignment of relative configurations of compound **32**.

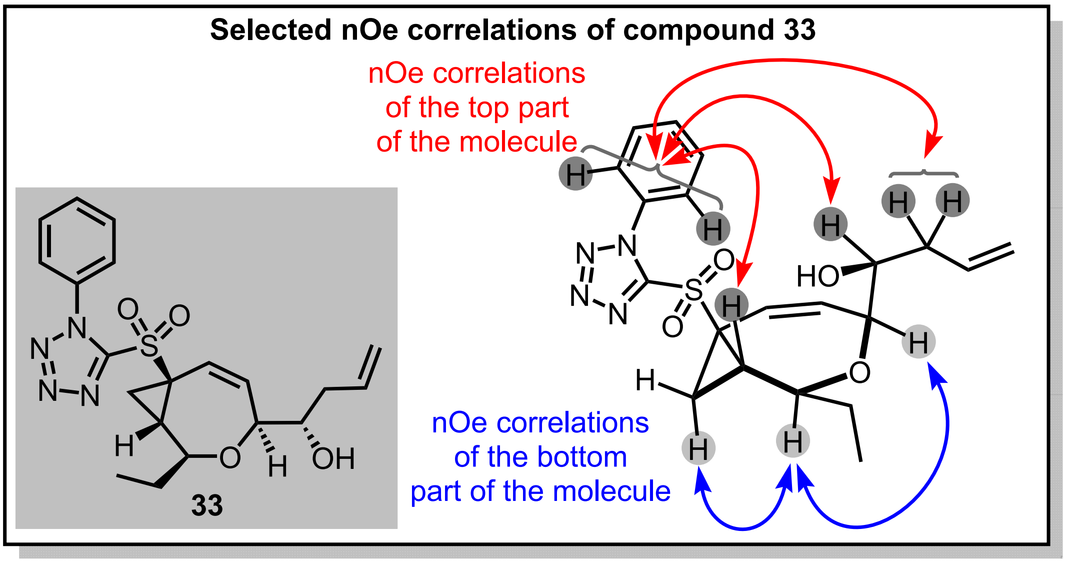

Figure S2. Assignment of relative configurations of compound **33**.

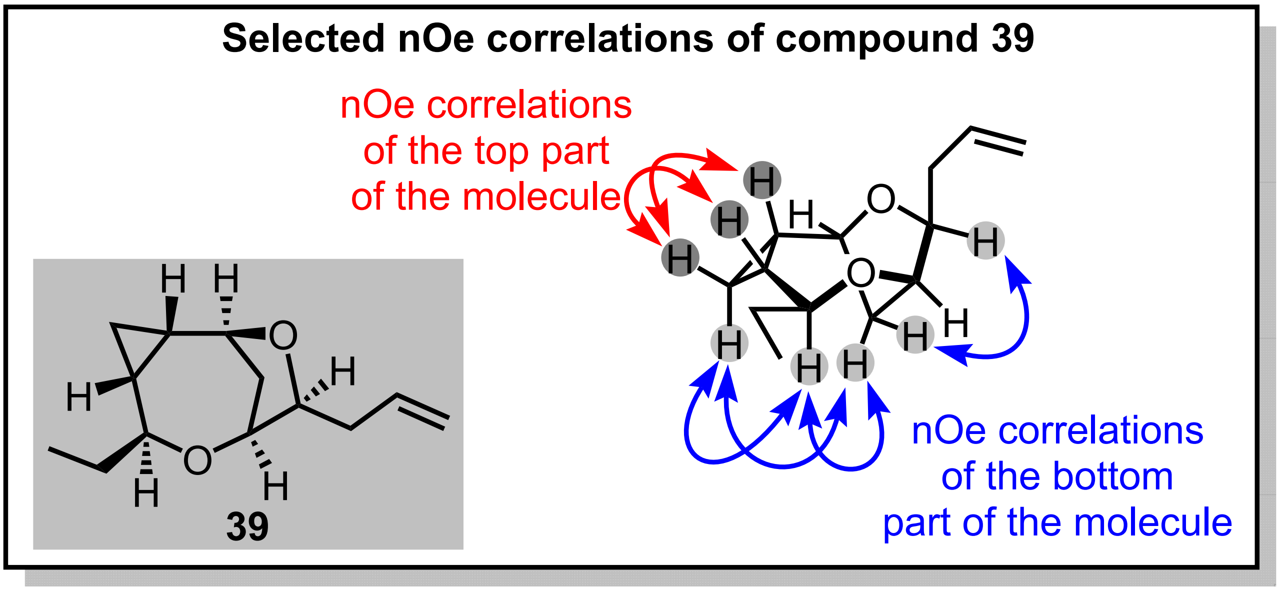

Figure 3. Assignment of relative configurations of compound **39**.

Atom No.	δ *S*-ester/ppm	δ *R*-ester/ppm	$\Delta\delta^{SR}$/ppm	$\Delta\delta^{SR}$/Hz
**15**	1.03	1.03	0.00	0
**14’**	1.89	1.90	-0.01	-5
**14**	1.61	1.61	0.00	0
**13**	3.86	3.87	-0.01	-5
**12**	4.15	4.27	-0.12	-60
**11’**	3.07	3.13	-0.06	-30
**11**	2.52	2.50	+0.02	+10
**10**	5.24	5.30	-0.06	-30
**9**	4.18	4.07	+0.11	+55
**8’**	2.15	2.07	+0.08	+40
**8**	2.01	1.90	+0.11	+55
**7**	3.99	3.96	+0.03	+15
**6**	3.73	3.70	+0.03	+15
**5’**	2.58	2.57	+0.01	+5
**5**	2.52	2.50	+0.02	+10
**4**	6.21	6.20	+0.01	+5
**3**	5.60	5.59	+0.01	+5
**2**			0.00	0
**1**	2.81	2.81	0.00	0

Table S2. Chemical shift data for Mosher ester analysis of laurefurenyne F (*E*)-**9**.

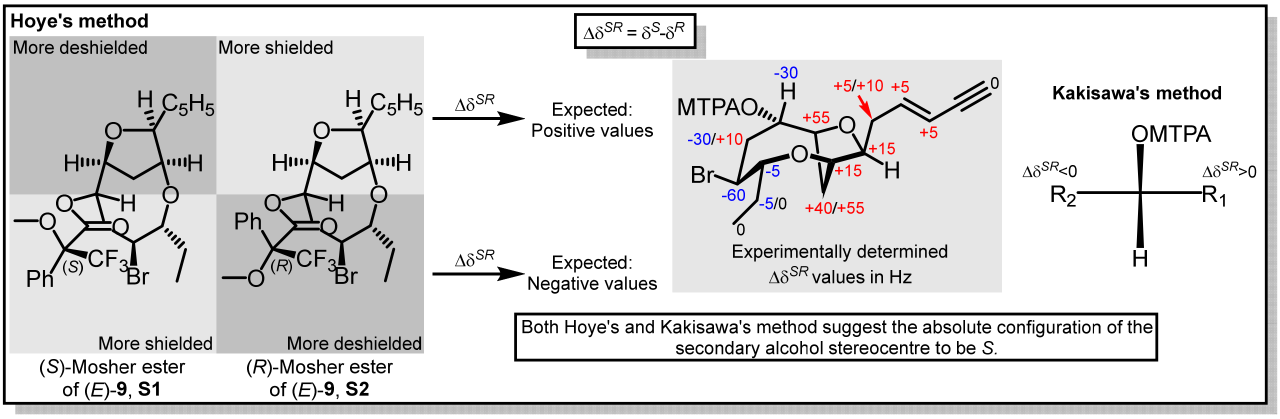

Figure S4. Mosher ester analysis of laurefurenyne F (*E*)-**9**.

Atom No.	δ *S*-ester/ppm	δ *R*-ester/ppm	$\Delta\delta^{SR}$/ppm	$\Delta\delta^{SR}$/Hz
**15**	1.03	1.04	-0.01	-5
**14'**	1.90	1.91	-0.01	-5
**14**	1.61	1.62	-0.01	-5
**13**	3.87	3.89	-0.02	-10
**12**	4.15	4.28	-0.13	-65
**11'**	3.10	3.17	-0.07	-35
**11**	2.52	2.50	+0.02	+10
**10**	5.26	5.32	-0.06	-30
**9**	4.19	4.07	+0.12	+60
**8'**	2.15	2.08	+0.07	+35
**8**	2.01	1.91	+0.10	+50
**7**	3.99	3.97	+0.02	+10
**6**	3.78	3.75	+0.03	+15
**5'**	2.83	2.82	+0.01	+5
**5**	2.71	2.71	0.00	0
**4**	6.04	6.04	0.00	0
**3**	5.56	5.56	0.00	0
**2**			0.00	0
**1**	3.10	3.11	-0.01	-5

Table S3. Chemical shift data for Mosher ester analysis of laurefurenyne E (*Z*)-**9**.

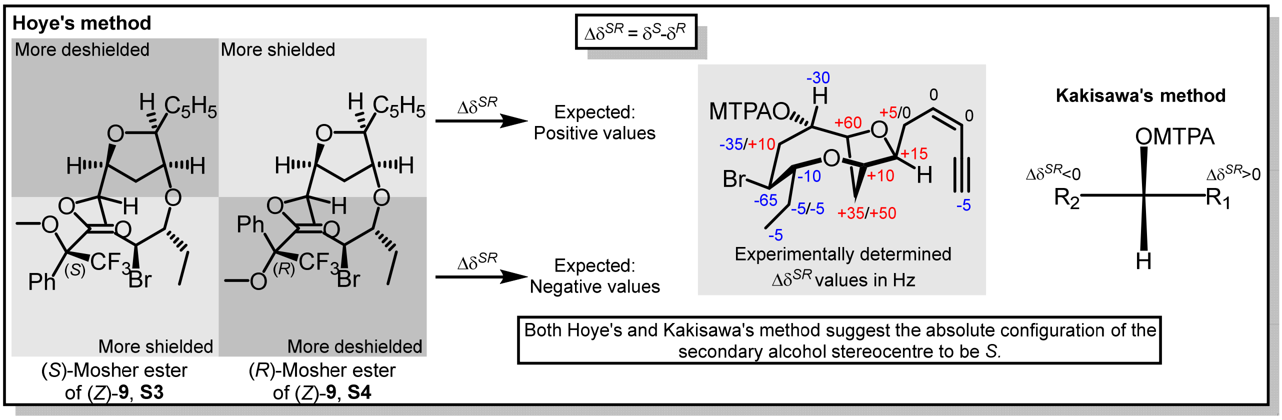

Figure S5. Mosher ester analysis of laurefurenyne E (*Z*)-**9**.

5) Comparative NMR data for natural products

Comparative ^{13}C NMR spectroscopic data for laurefurenyne F (*E*)-**9**

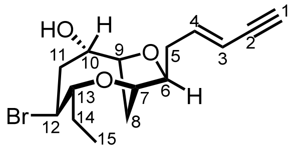

Spectrometer Frequency/MHz	101	-	125	-	101
**NMR solvent**	$CDCl_3$	-	$CDCl_3$	-	$CDCl_3$
**Atom No.**	**Ref. [6]/ppm (Main text)**	**Δδ ([6]-synthetic)**	**Synthetic/ppm**	**Δδ ([6]-synthetic)**	**Ref. [6]/ppm (FID)**
**1**	76.6	0.2	76.4	0.0	76.4
**2**	81.8	-0.6	82.4	-	Not found
**3**	111.3	0.0	111.3	0.0	111.3
**4**	142.1	-0.2	142.3	0.0	142.3
**5**	32.8	0.0	32.8	0.0	32.8
**6**	83.4	0.0	83.4	0.0	83.4
**7**	70.1	0.1	70.0	0.1	70.1
**8**	33.2	0.1	33.1	0.0	33.1
**9**	78.4	-0.5	78.9	0.0	78.9
**10**	70.5	0.0	70.5	0.0	70.5
**11**	38.9	-0.1	39.0	-0.1	38.9
**12**	51.8	0.0	51.8	0.0	51.8
**13**	83.5	0.0	83.5	0.0	83.5
**14**	23.2	0.0	23.2	0.0	23.2
**15**	11.9	0.0	11.9	0.0	11.9
**Systematic Shift/ppm**		0.0		0.0	

Table S4. Comparison of ^{13}C NMR chemical shifts of synthetic laurefurenyne F (*E*)-**9** with natural laurefurenyne F (*E*)-**9**. The ^{13}C NMR chemical shifts of natural laurefurenyne F (*E*)-**9** were extracted from the main text in its isolation paper,[6] and extracted from the ^{13}C NMR FID provided by Prof. Marcel Jaspars. The ^{13}C NMR chemical shift of C-9 of natural laurefurenyne F (*E*)-**9** was found to be 78.4 ppm in the main text of the isolation paper,[6] and 78.9 ppm from the ^{13}C NMR FID provided by Prof. Marcel Jaspars. The ^{13}C NMR chemical shift of the quaternary carbon C-2 of natural laurefurenyne F (*E*)-**9** was found to be 81.8 ppm in the main text of the isolation paper,[6] and not found from the ^{13}C NMR FID provided by Prof. Marcel Jaspars.

Comparative ^{13}C NMR spectroscopic data for laurefurenyne E (*Z*)-**9**					
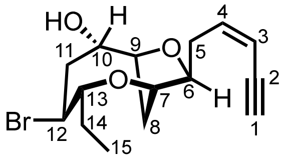					
**Spectrometer Frequency/MHz**	101	-	125	-	101
**NMR solvent**	$CDCl_3$	-	$CDCl_3$	-	$CDCl_3$
**Atom No.**	**Ref. [6]/ppm (Main text)**	**Δδ ([6]-synthetic)**	**Synthetic/ppm**	**Δδ ([6]-synthetic)**	**Ref. [6]/ppm (FID)**
**1**	81.7	-0.4	82.1	0.0	82.1
**2**	80.7	0.4	80.3	0.0	80.3
**3**	110.2	-0.2	110.4	0.0	110.4
**4**	141.5	-0.2	141.7	0.0	141.7
**5**	30.1	-0.2	30.3	0.0	30.3
**6**	83.2	-0.2	83.4	0.0	83.4
**7**	70.2	-0.1	70.3	0.1	70.4
**8**	33.1	-0.1	33.2	0.0	33.2
**9**	78.6	-0.2	78.8	0.0	78.8
**10**	70.5	-0.1	70.6	0.0	70.6
**11**	38.8	-0.1	38.9	0.0	38.9
**12**	51.8	-0.2	52.0	0.0	52.0
**13**	83.2	-0.2	83.4	0.0	83.4
**14**	23.1	-0.2	23.3	-0.1	23.2
**15**	11.8	-0.2	12.0	0.0	12.0
**Systematic Shift/ppm**		-0.2		0.0	

Table S5. Comparison of ^{13}C NMR chemical shifts of synthetic laurefurenyne E (*Z*)-**9** with natural laurefurenyne E (*Z*)-**9**. The ^{13}C NMR chemical shifts of natural laurefurenyne E (*Z*)-**9** were extracted from the main text in its isolation paper,[6] and extracted from the ^{13}C NMR FID provided by Prof. Marcel Jaspars.

Comparative ^{13}C NMR spectroscopic data for laurefurenyne D (*E*)-**10** 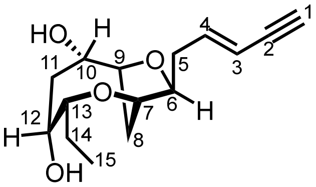					
**Spectrometer Frequency/MHz**	101	-	125	-	101
**NMR solvent**	$CDCl_3$	-	$CDCl_3$	-	$CDCl_3$
**Atom No.**	**Ref. [6]/ppm (Main text)**	**Δδ ([6]-synthetic)**	**Synthetic/ppm**	**Δδ ([6]-synthetic)**	**Ref. [6]/ppm (FID)**
**1**	76.2	-0.2	76.4	0.0	76.4
**2**	82.2	-0.2	82.4	0.0	82.4
**3**	110.8	-0.3	111.1	0.0	111.1
**4**	142.5	-0.2	142.7	0.0	142.7
**5**	32.8	-0.2	33.0	0.0	33.1
**6**	82.9	-0.3	83.2	-0.1	83.1
**7**	70.2	-0.3	70.5	-0.1	70.4
**8**	31.7	-0.2	31.9	0.0	31.9
**9**	79.7	-0.2	79.9	0.0	79.9
**10**	73.4	-0.2	73.6	0.0	73.6
**11**	29.7	-0.5	30.2	-0.1	30.1
**12**	73.1	-0.1	73.2	0.0	73.2
**13**	79.3	-0.2	79.5	0.0	79.5
**14**	22.3	-0.3	22.6	0.0	22.6
**15**	11.4	-0.1	11.5	0.0	11.5
**Systematic Shift/ppm**		-0.2		0.0	

Table S6. Comparison of ^{13}C NMR chemical shifts of synthetic laurefurenyne D (*E*)-**10** with natural laurefurenyne D (*E*)-**10**. The ^{13}C NMR chemical shifts of natural laurefurenyne D (*E*)-**10** were extracted from the main text in its isolation paper,[6] and extracted from the ^{13}C NMR FID provided by Prof. Marcel Jaspars. The ^{13}C NMR chemical shift of C-11 of natural laurefurenyne D (*E*)-**10** was found to be 29.7 ppm in the main text of the isolation paper,[6] and 30.1 ppm from the ^{13}C NMR FID provided by Prof. Marcel Jaspars.

Comparative ^{13}C NMR spectroscopic data for laurefurenyne C (*Z*)-**10** 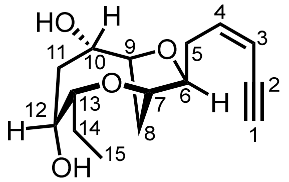					
**Spectrometer Frequency/MHz**	101	-	125	-	101
**NMR solvent**	$CDCl_3$	-	$CDCl_3$	-	$CDCl_3$
**Atom No.**	**Ref. [6]/ppm (Main text)**	**Δδ ([6]-synthetic)**	**Synthetic/ppm**	**Δδ ([6]-synthetic)**	**Ref. [6]/ppm (FID)**
**1**	81.9	-0.1	82.0	0.1	82.1
**2**	Not found	-	80.3	-	Not found
**3**	110.0	-0.2	110.2	0.0	110.2
**4**	141.8	-0.2	142.0	0.0	142.0
**5**	30.4	-0.1	30.5	0.0	30.5
**6**	83.0	-0.2	83.2	0.0	83.2
**7**	71.0	-0.2	71.2	0.0	71.2
**8**	32.0	-0.1	32.1	0.0	32.1
**9**	79.5	-0.2	79.7	0.0	79.7
**10**	73.3	-0.2	73.5	0.0	73.5
**11**	30.8	-0.2	31.0	-0.1	30.9
**12**	72.8	-0.2	73.0	0.0	73.0
**13**	78.9	-0.3	79.2	0.0	79.2
**14**	23.1	-0.2	23.3	0.0	23.3
**15**	11.3	-0.1	11.4	0.0	11.4
**Systematic Shift/ppm**		-0.2		0.0	

Table S7. Comparison of ^{1}H and ^{13}C NMR chemical shifts of laurefurenyne C (*Z*)-**10** with natural laurefurenyne C (*Z*)-**10**.[6] The ^{13}C NMR chemical shift of the quaternary carbon C-2 of natural laurefurenyne C (*Z*)-**10** was not found in the main text of the isolation paper,[6] nor from the ^{13}C NMR FID provided by Prof. Marcel Jaspars.

Comparative ^{13}C NMR spectroscopic data: Synthetic *ent*-deacetyllaurencin **23** versus natural deacetyllaurencin *ent*-**23**.

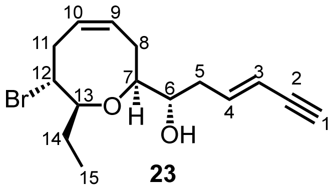

23

**Spectrometer Frequency/MHz**	101	-	125
**NMR solvent**	$CDCl_3$	-	$CDCl_3$
**Atom No.**	**Ref. [11]/ppm**	**Δδ ([11]-synthetic)**	**Synthetic/ppm**
**1**	77.0	-0.1	77.1
**2**	82.4	-0.1	82.5
**3**	111.4	-0.1	111.5
**4**	142.7	-0.1	142.8
**5**	37.1	-0.1	37.2
**6**	73.3	0.0	73.3
**7**	83.6	-0.1	83.7
**8**	30.4	0.0	30.4
**9**	129.9	0.0	129.9
**10**	128.6	0.0	128.6
**11**	32.5	0.0	32.5
**12**	56.0	-0.1	56.1
**13**	83.6	-0.1	83.7
**14**	25.9	-0.1	26.0
**15**	9.1	0.0	9.1
**Systematic Shift/ppm**		-0.1	

Table S8. Comparison of ^{1}H and ^{13}C NMR chemical shifts of *ent*-deacetyllaurencin **23** and natural deacetyllaurencin *ent*-**23**.[11]

Synthetic *ent*-laurencin **24** versus natural deacetyllaurencin *ent*-**24** and literature synthetic laurencin **24.**

Comparison of ¹H and ¹³C chemical shifts															
	Synthetic *ent*- laurencin 500/126 MHz, $CDCl_3$						Literature synthetic laurencin 500/62.5 MHz, $CDCl_3$						Natural laurencin 300/76 MHz, $CDCl_3$		
Atom No.	¹H/ppm	Multiplicity/Hz	¹³C/ppm	Δδ¹H	Δδ¹³C	Atom No.	¹H/ppm	Multiplicity/Hz	¹³C/ppm	Δδ¹H	Δδ¹³C	Atom No.	¹H/ppm	Multiplicity/Hz	¹³C/ppm
**1**	2.82	d, 2.2	77.0	0.00	0.3	**1**	2.82	d, 1.5	76.7	0.01	-	**1**	2.83	d, 2.0	-
**2**			82.0	0.00	0.2	**2**			81.8	0.00	-	**2**			-
**3**	5.53	dq, 15.9, 1.7	111.8	0.00	0.2	**3**	5.53	dd, 16.0, 1.5	111.6	-0.01	-	**3**	5.52	d, 15.0	-
**4**	6.15	dt, 15.2, 7.3	141.3	-0.01	0.2	**4**	6.16	dt, 16.0, 7.2	141.1	-0.01	-	**4**	6.15	dt, 15.0, 7.0	-
**5**	2.41	m	34.0	-	0.2	**5**	2.43	m	33.8	-	-	**5**	2.40	-	-
**5'**	2.50	m				**5'**						**5'**			
**6**	5.00	dt, 8.7, 4.3	74.2	0.00	0.1	**6**	5.00	dt, 8.7, 4.4	74.1	-0.02	-	**6**	4.98	dt, 8.0, 5.0	-
**7**	3.39	dd, 10.6, 4.6	81.5	0.00	0.1	**7**	3.39	dd, 10.5, 4.4	81.4	0.01	-	**7**	3.40	d, 5.0	-
**8**	2.08	m	29.8	0.00	0.1	**8**	2.08	m	29.7	0.12	-	**8**	2.20	-	-
**8'**	2.35	m		-0.08		**8'**	2.43	m		-0.03		**8'**	2.40	-	
**9**	5.93	m	129.4	-	0.2	**9**	5.91	m	129.2	-	-	**9**	5.90	m	-
**10**	5.89	m	129.1		0.1	**10**			129.0		-	**10**	5.90	m	-
**11**	2.46	m	32.5	0.03	0.2	**11**	2.43	m	32.3	-0.03	-	**11**	2.40	-	-
**11'**	3.15	ddd, 13.8, 8.5, 3.6		-0.01		**11'**	3.16	ddd, 14.0, 8.5, 3.4		0.04		**11'**	3.20	-	
**12**	4.07	dt, 10.0, 3.4	56.2	0.00	0.2	**12**	4.07	dt, 9.9, 3.4	56.0	0.00	-	**12**	4.07	dt, 9.0, 3.0	-
**13**	3.43	ddd, 9.8, 7.2, 2.6	84.7	0.00	0.1	**13**	3.43	ddd, 9.9, 7.4, 2.6	84.6	-0.03	-	**13**	3.40	ddd, 9.0, 7.0, 3.0	-
**14**	1.59	dp, 14.8, 7.4	25.9	0.02	0.1	**14**	1.57	dq, 14.4, 7.4	25.8	-0.01	-	**14**	1.56	-	-
**14'**	1.95	dqd, 14.8, 7.4, 2.6		0.00		**14'**	1.95	ddq, 14.4, 7.4, 2.6		0.02		**14'**	1.97	-	
**15**	0.98	t, 7.4	9.5	0.00	0.2	**15**	0.98	t, 7.4	9.3	0.00	-	**15**	0.98	t, 7.0	-
**Ac**	2.08	s	21.2	0.00	0.2	**Ac**	2.08	s	21.0	-0.05	-	**Ac**	2.03	s	-
**Ac CO**			170.5	0.00	0.2	**Ac CO**			170.3	0.00	-	**Ac CO**			-
					0.2		¹³C NMR systematic shift/ppm								

Table S9. Comparison of ¹H and ¹³C NMR chemical shifts of *ent*-laurencin **24** with natural laurencin *ent*-**24**[10,19] and literature synthetic laurencin *ent*-**24**.[12]

NMR spectroscopic data of other synthetic laurencin *ent*-**24** (not listed here) are available in their respective publications.[13–19,22]

6) Comparative specific rotation data for natural products

Synthetic laurefurenyne F (*E*)-**9** versus natural laurefurenyne F (*E*)-**9**.

Comparison of specific rotations					
**Natural Product**	**T/°C**	**λ/nm**	**c (g/100 mL)**	**Solvent**	**Measured specific rotation**
Synthetic laurefurenyne F (*E*)-**9**	25	589	0.12	MeOH	+17.5
Natural laurefurenyne F[6] (*E*)-**9**	25	589	0.10	MeOH	+17.0

Table S10. Comparison of specific rotations of laurefurenyne F (*E*)-**9** and natural laurefurenyne F (*E*)-**9**.

Synthetic laurefurenyne E (*Z*)-**9** versus natural laurefurenyne E (*Z*)-**9**.

Comparison of specific rotations					
**Natural Product**	**T/°C**	**λ/nm**	**c (g/100 mL)**	**Solvent**	**Measured specific rotation**
Synthetic laurefurenyne E (*Z*)-**9**	25	589	0.17	MeOH	-4.7 (*Z*:*E* = 7.8:1)
Synthetic laurefurenyne E (*Z*)-**9**	25	589	0.10	MeOH	-5.9 (*Z*:*E* > 15:1)
Natural laurefurenyne E[6] (*Z*)-**9**	25	589	0.10	MeOH	+11.0

Table S11. Comparison of specific rotations of laurefurenyne E (*Z*)-**9** and natural laurefurenyne E (*Z*)-**9**.

Synthetic laurefurenyne D (*E*)-**10** versus natural laurefurenyne D (*E*)-**10**.

**Comparison of specific rotations**					
**Natural Product**	**T/°C**	**λ/nm**	**c (g/100 mL)**	**Solvent**	**Measured specific rotation**
Synthetic laurefurenyne D (*E*)-**10**	25	589	0.10	MeOH	+14.0
Natural laurefurenyne D[6] (*E*)-**10**	17	589	0.10	MeOH	+32.0

Table S12. Comparison of specific rotations of laurefurenyne D (*E*)-**10** with natural laurefurenyne D (*E*)-**10**.

Synthetic laurefurenyne D (*Z*)-**10** versus natural laurefurenyne D (*Z*)-**10**.

**Comparison of specific rotations**					
**Natural Product**	**T/°C**	**λ/nm**	**c (g/100 mL)**	**Solvent**	**Measured specific rotation**
Synthetic laurefurenyne C (*Z*)-**10**	25	589	0.13	MeOH	+10.8
Natural laurefurenyne C[6] (*Z*)-**10**	17	589	0.10	MeOH	+20.0

Table S13. Comparison of specific rotations of laurefurenyne C (*Z*)-**10** with natural laurefurenyne C (*Z*)-**10**.

Synthetic *ent*-deacetyllaurencin **23** versus natural deacetyllaurencin *ent*-**23**.

**Comparison of specific rotations**					
**Natural Product**	**T/°C**	**λ/nm**	**c (g/100 mL)**	**Solvent**	**Measured specific rotation**
Synthetic *ent*-deacetyllaurencin **23**	25	589	0.05	$CHCl_3$	-34.7
From hydrolysis of natural laurencin[10] *ent*-**23**	17	589	1.15	$CHCl_3$	+46.1
Natural deacetyllaurencin[11] *ent*-**23**	25	589	0.4	$CHCl_3$	+35.5

Table S14. Comparison of specific rotations of *ent*-deacetyllaurencin **23** with deacetyllaurencin obtained from hydrolysis of natural deacetyllaurencin *ent*-**23** and natural deacetyllaurencin *ent*-**23**.

Synthetic *ent*-laurencin **24** versus natural laurencin *ent*-**24** and literature synthetic laurencin *ent*-**24**.

**Comparison of specific rotations**					
**Natural Product**	**T/°C**	**λ/nm**	**c (g/100 mL)**	**Solvent**	**Measured specific rotation**
Synthetic *ent*-laurencin **24**	25	589	0.10	$CHCl_3$	-61.0
Literature synthetic laurencin[12]* *ent*-**24**	20	589	0.05	$CHCl_3$	+70.0
Natural laurencin[10,21] *ent*-**24**	17	589	1.00	$CHCl_3$	+70.2

Table S15. Comparison of specific rotations of *ent*-laurencin **24**, natural laurencin *ent*-**24** and literature synthetic laurencin *ent*-**24**.

* Specific rotation data of other synthetic laurencin *ent*-**24** (not listed here) are available in their respective publications.[13–19,22]

7) NMR Spectra

(1*S*,3*S*,4*R*,6*S*,7*S*,9*S*)-9-Allyl-4-bromo-3-ethyl-2,8-dioxabicyclo[5.2.1]decan-6-ol 17 (500MHz, $CDCl_3$)

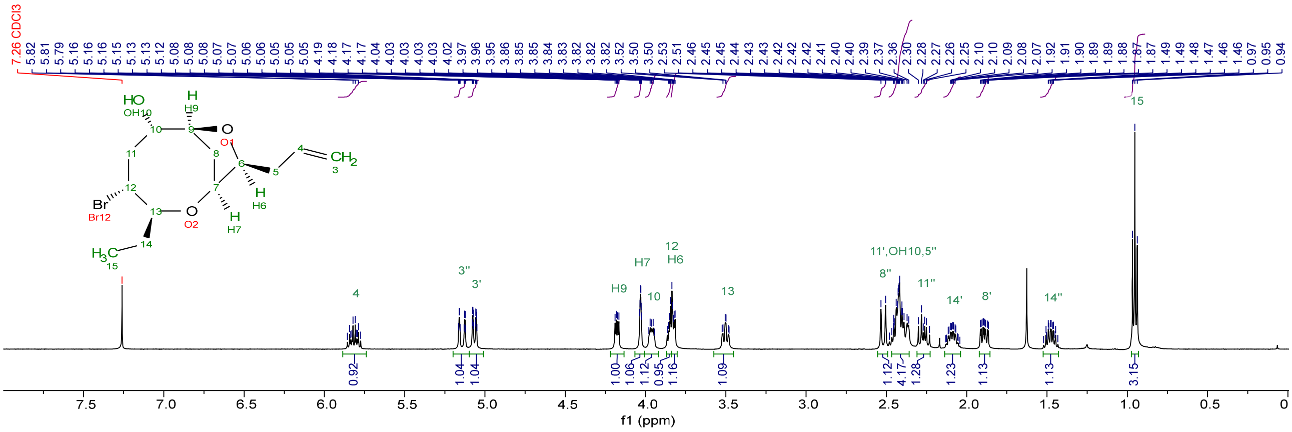

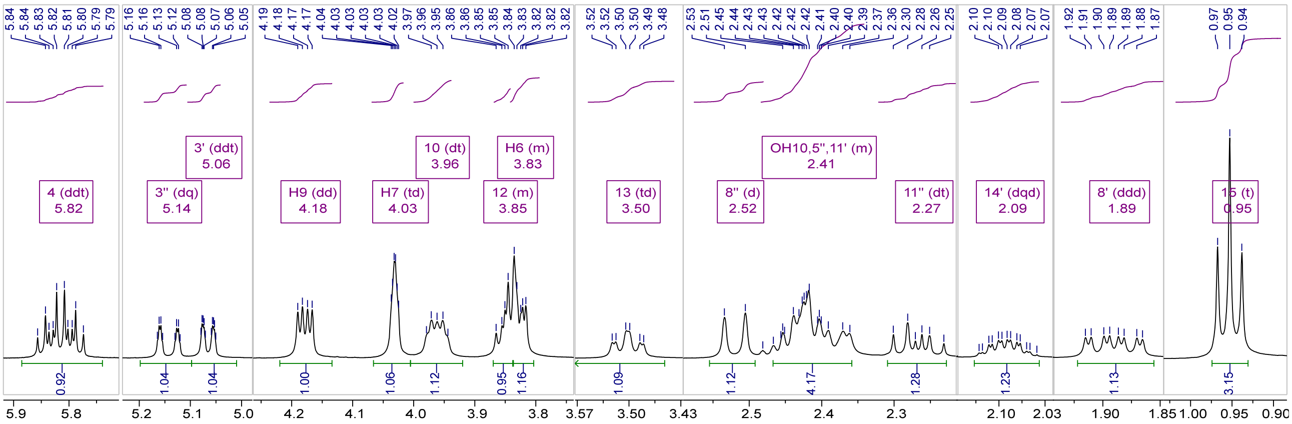

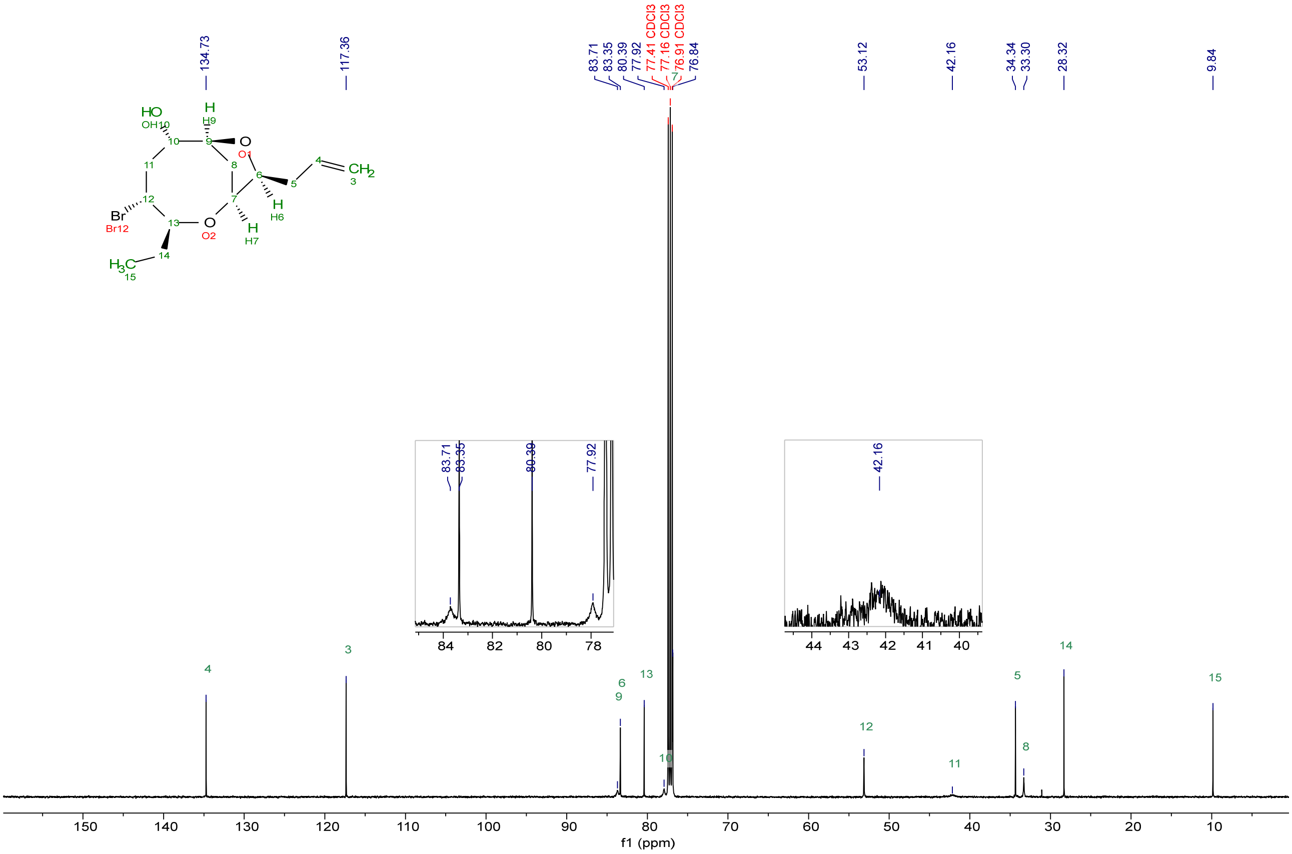

(1*S*,3*S*,4*R*,6*S*,7*S*,9*S*)-4-Bromo-3-ethyl-9-((*E*)-pent-2-en-4-yn-1-yl)-2,8-dioxabicyclo[5.2.1]decan-6-ol, *ent*-(*E*)-laurefucin 18 (Upper spectrum: 500MHz, C_6D_6. Lower spectrum: 400MHz, C_6D_6.)

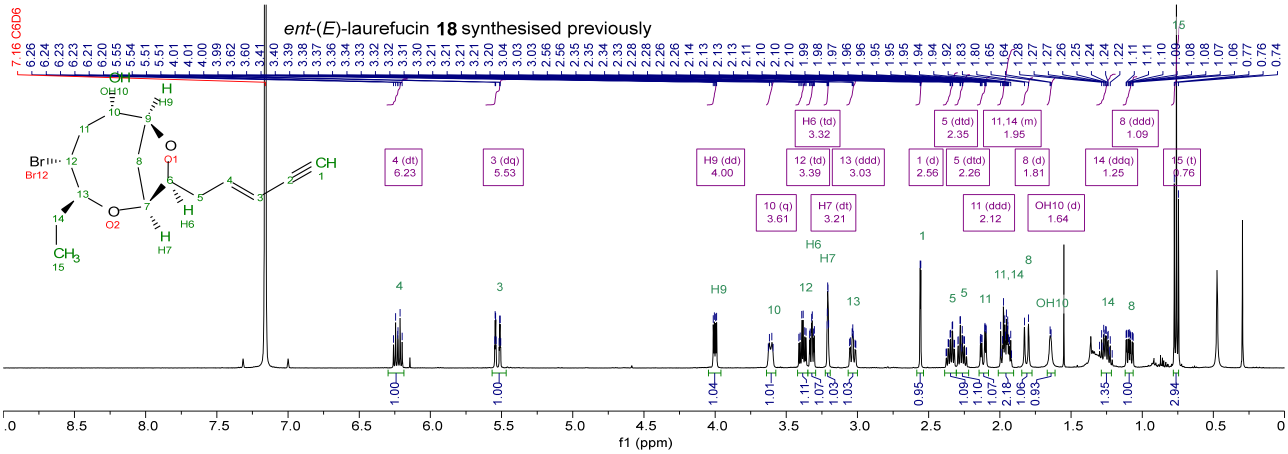

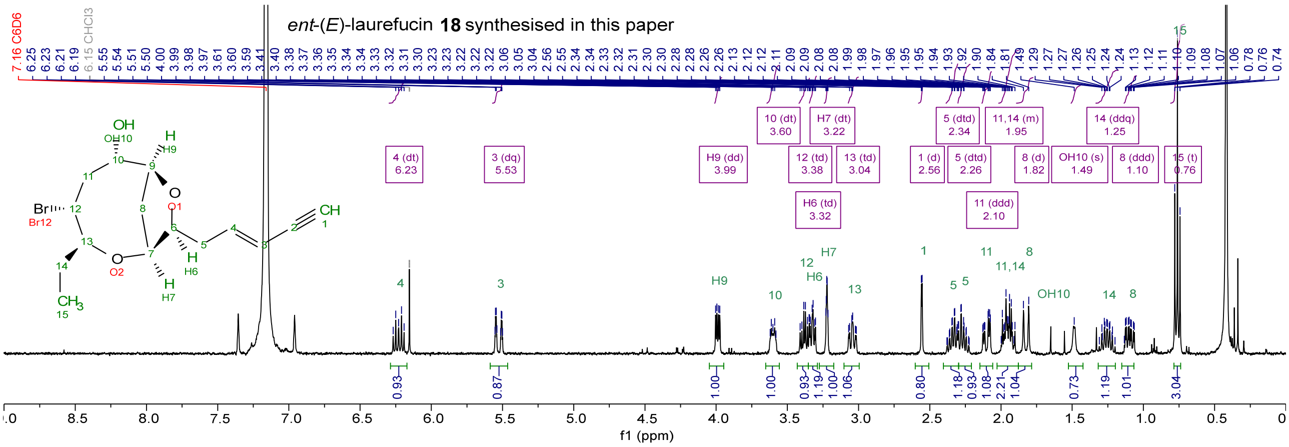

(1*S*,3*R*,4*S*,6*S*,7*S*,9*S*)-9-Allyl-4-bromo-3-ethyl-2,8-dioxabicyclo[5.2.1]decan-6-ol 12 (500MHz, $CDCl_3$)

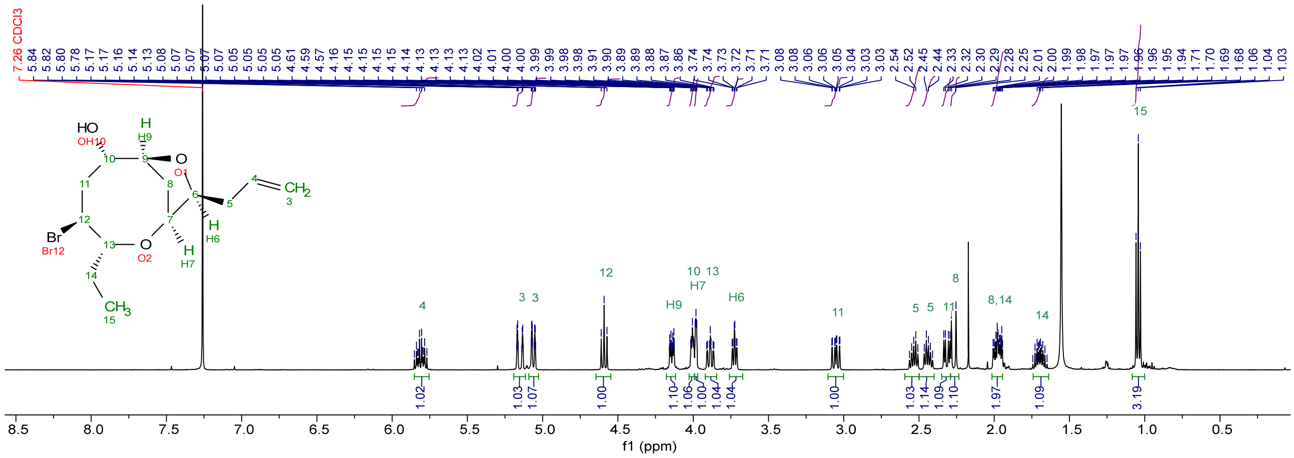

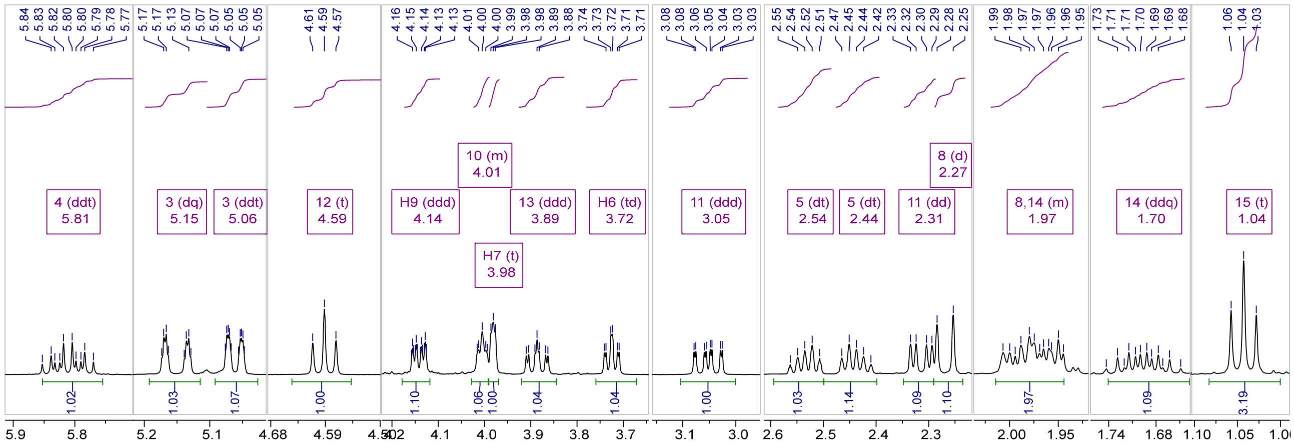

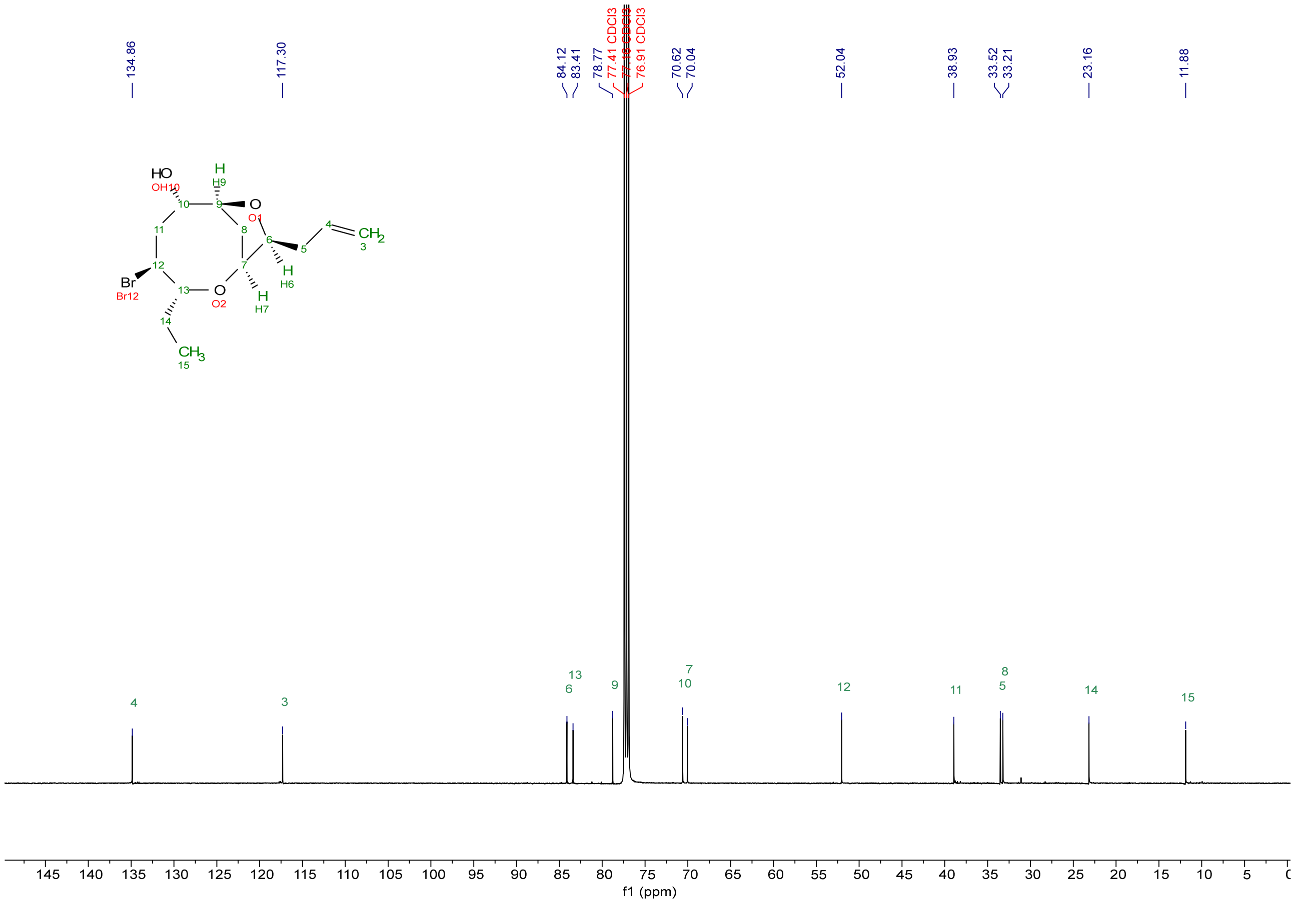

(1*S*,3*R*,4*S*,6*S*,7*S*,9*S*)-4-Bromo-3-ethyl-9-((*E*)-pent-2-en-4-yn-1-yl)-2,8-dioxabicyclo [5.2.1]decan-6-ol, laurefurenyne F (*E*)-9 (500MHz, $CDCl_3$)

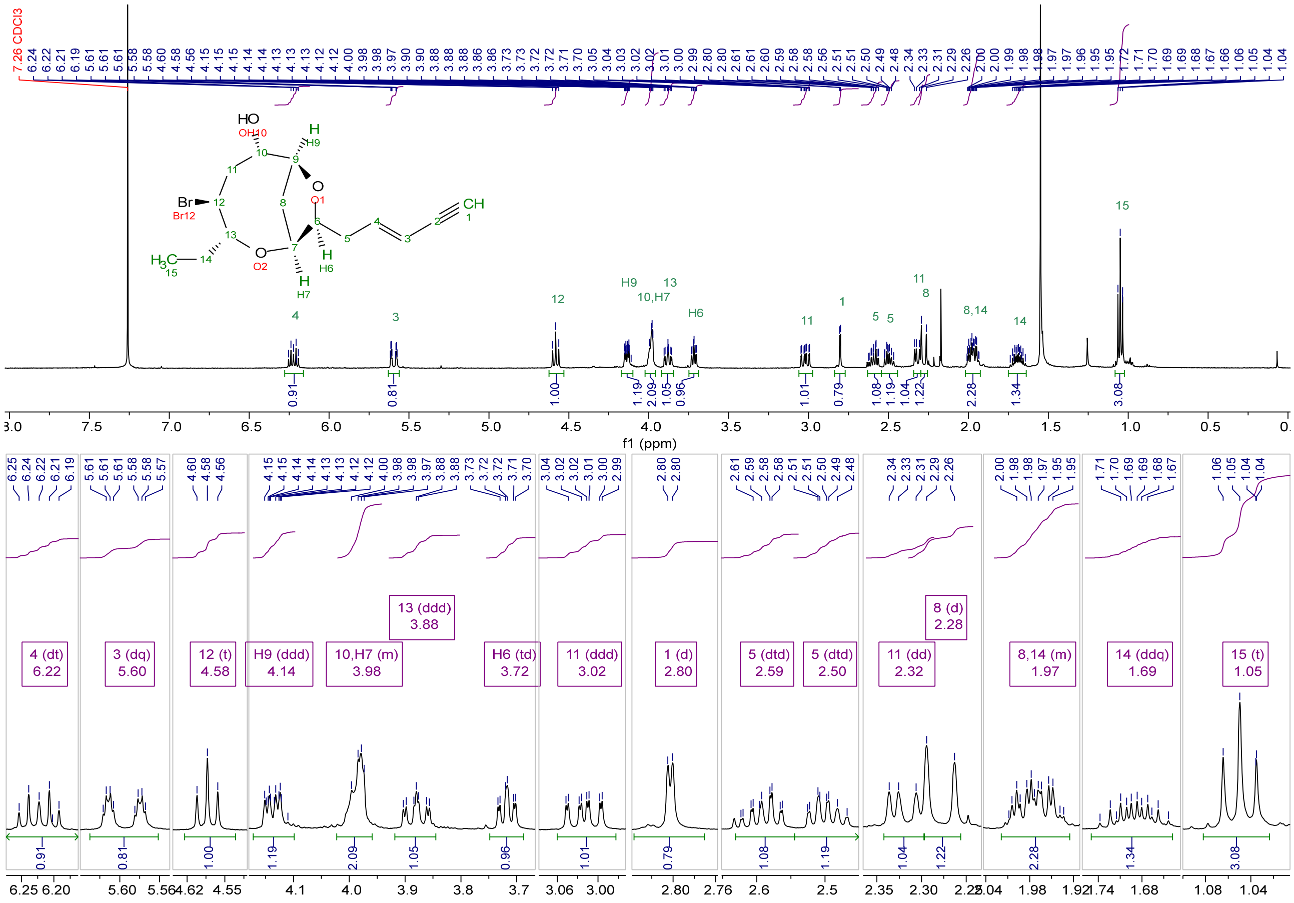

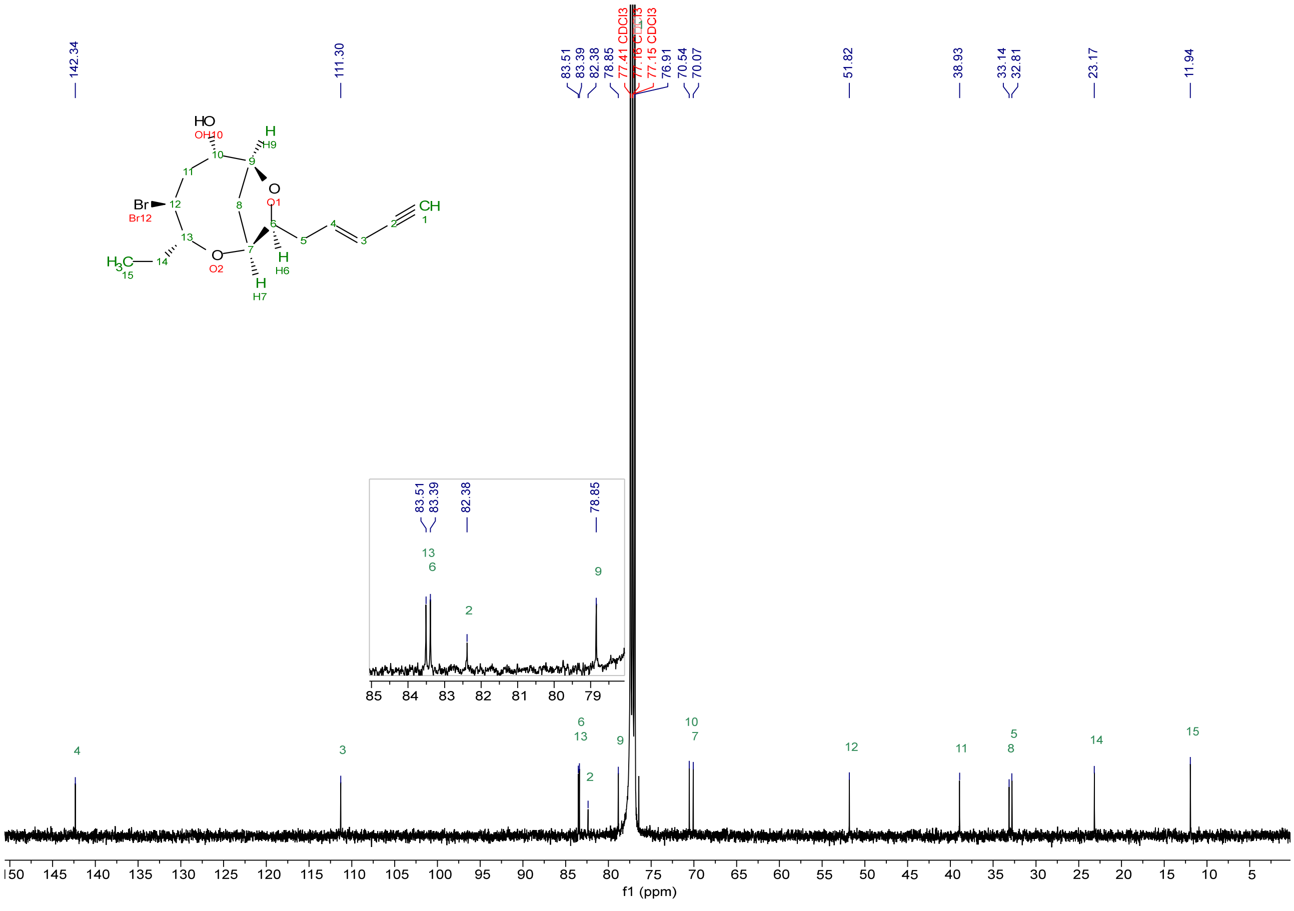

(1*S*,3*R*,4*S*,6*S*,7*S*,9*S*)-4-Bromo-3-ethyl-9-((*E*)-pent-2-en-4-yn-1-yl)-2,8-dioxabicyclo [5.2.1]decan-6-yl (*S*)-3,3,3-trifluoro-2-methoxy-2-phenylpropanoate S1 (500MHz, $CDCl_3$)

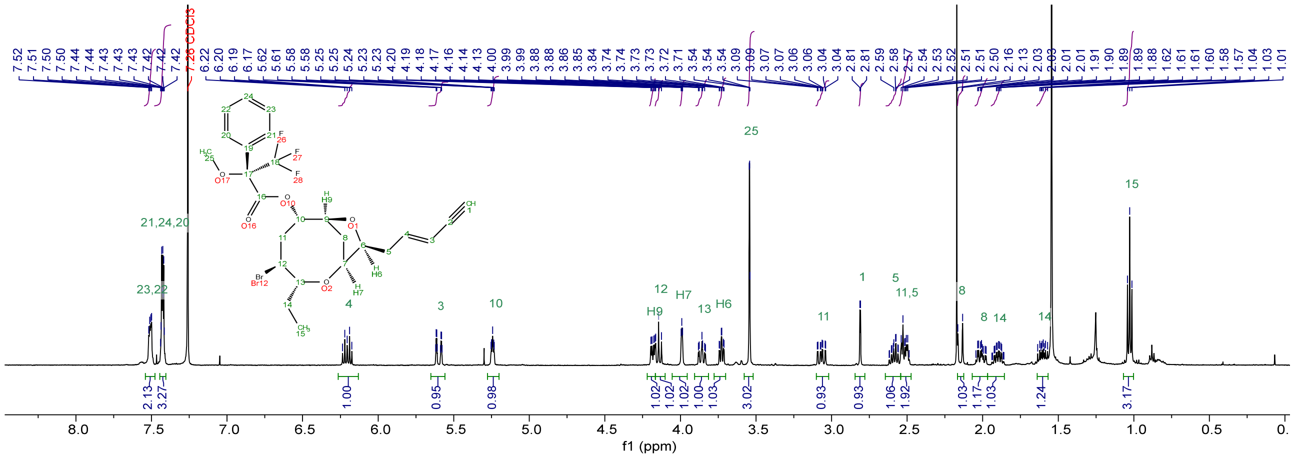

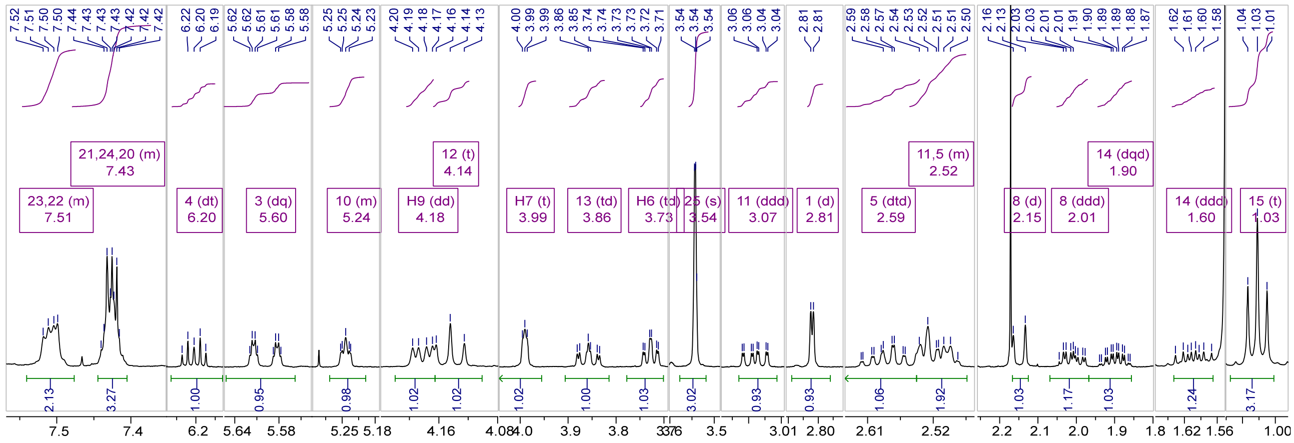

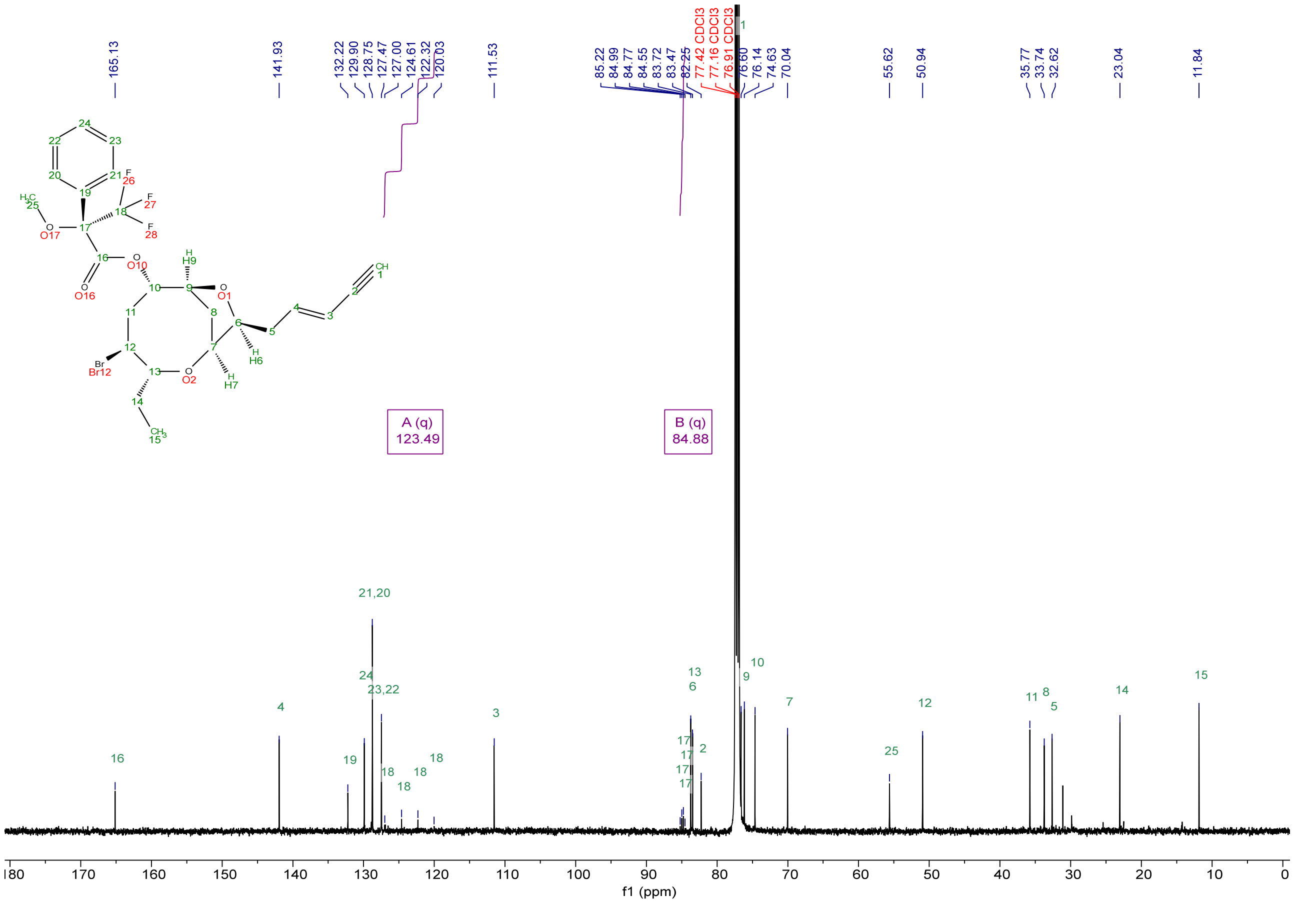

(1*S*,3*R*,4*S*,6*S*,7*S*,9*S*)-4-Bromo-3-ethyl-9-((*E*)-pent-2-en-4-yn-1-yl)-2,8-dioxabicyclo [5.2.1]decan-6-yl (*R*)-3,3,3-trifluoro-2-methoxy-2-phenylpropanoate S2 (500MHz, $CDCl_3$)

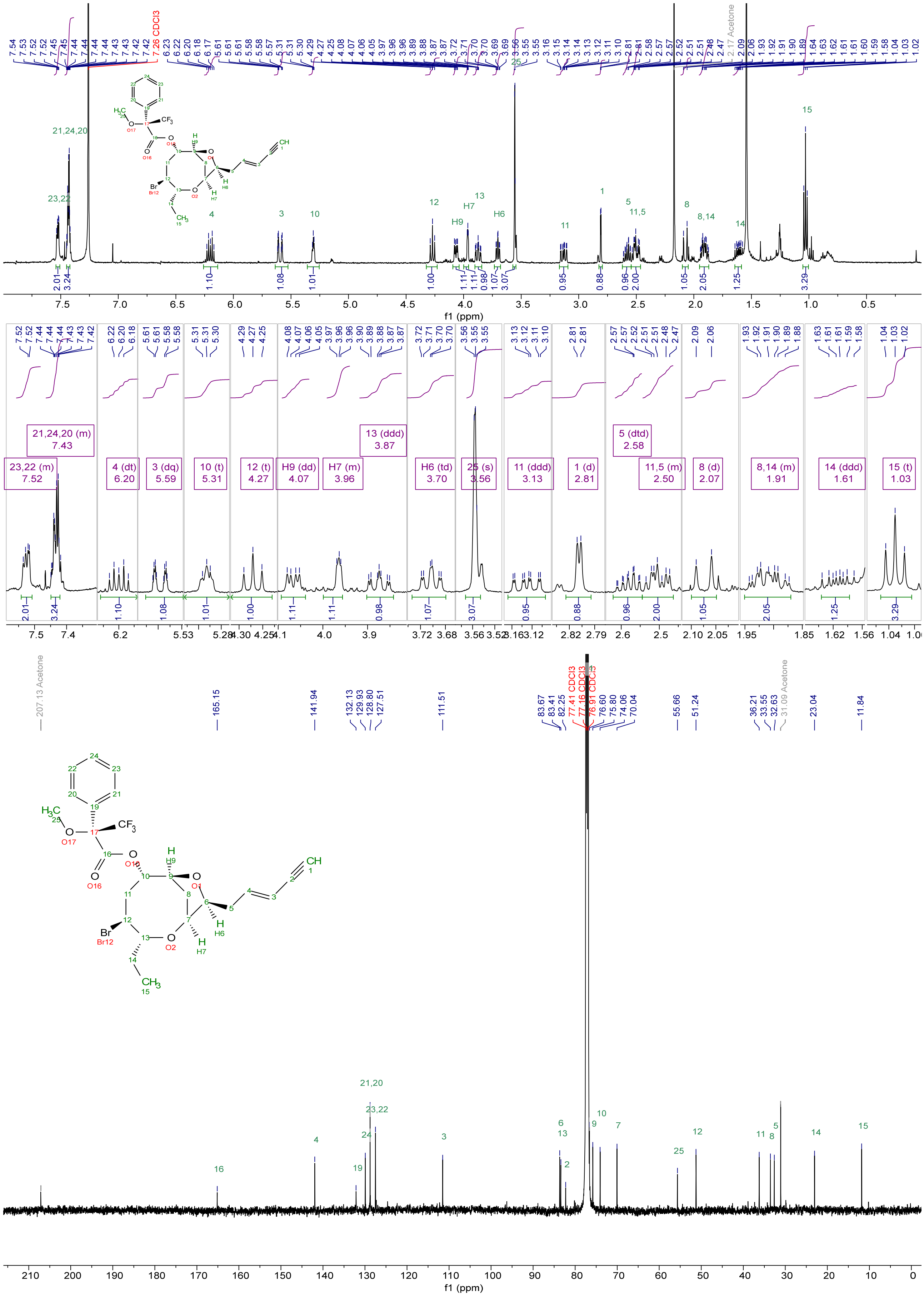

(1*S*,3*R*,4*S*,6*S*,7*S*,9*S*)-4-Bromo-3-ethyl-9-((*Z*)-p*ent*-2-en-4-yn-1-yl)-2,8-dioxabicyclo [5.2.1]decan-6-ol, laurefurenyne E (*Z*)-9 (500MHz, $CDCl_3$)

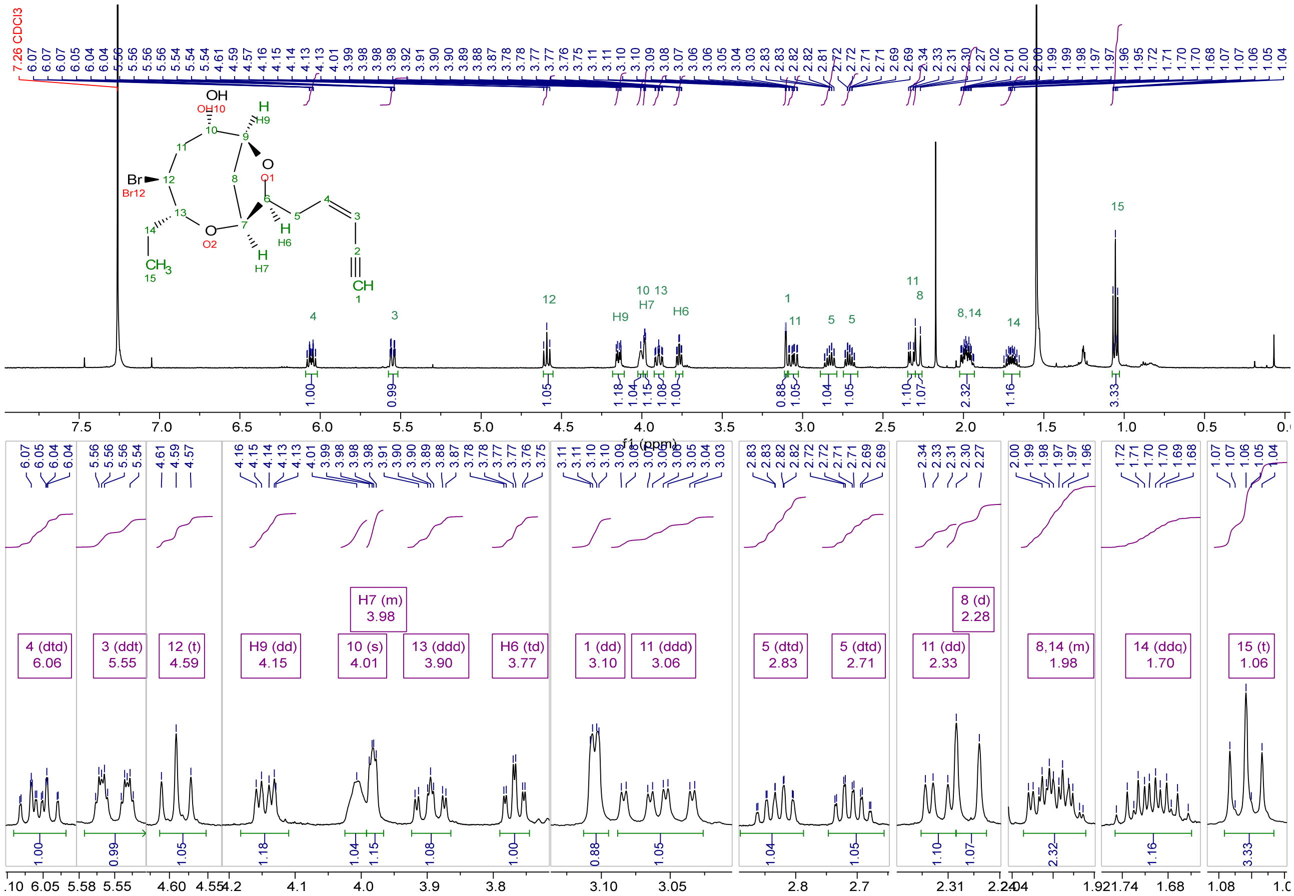

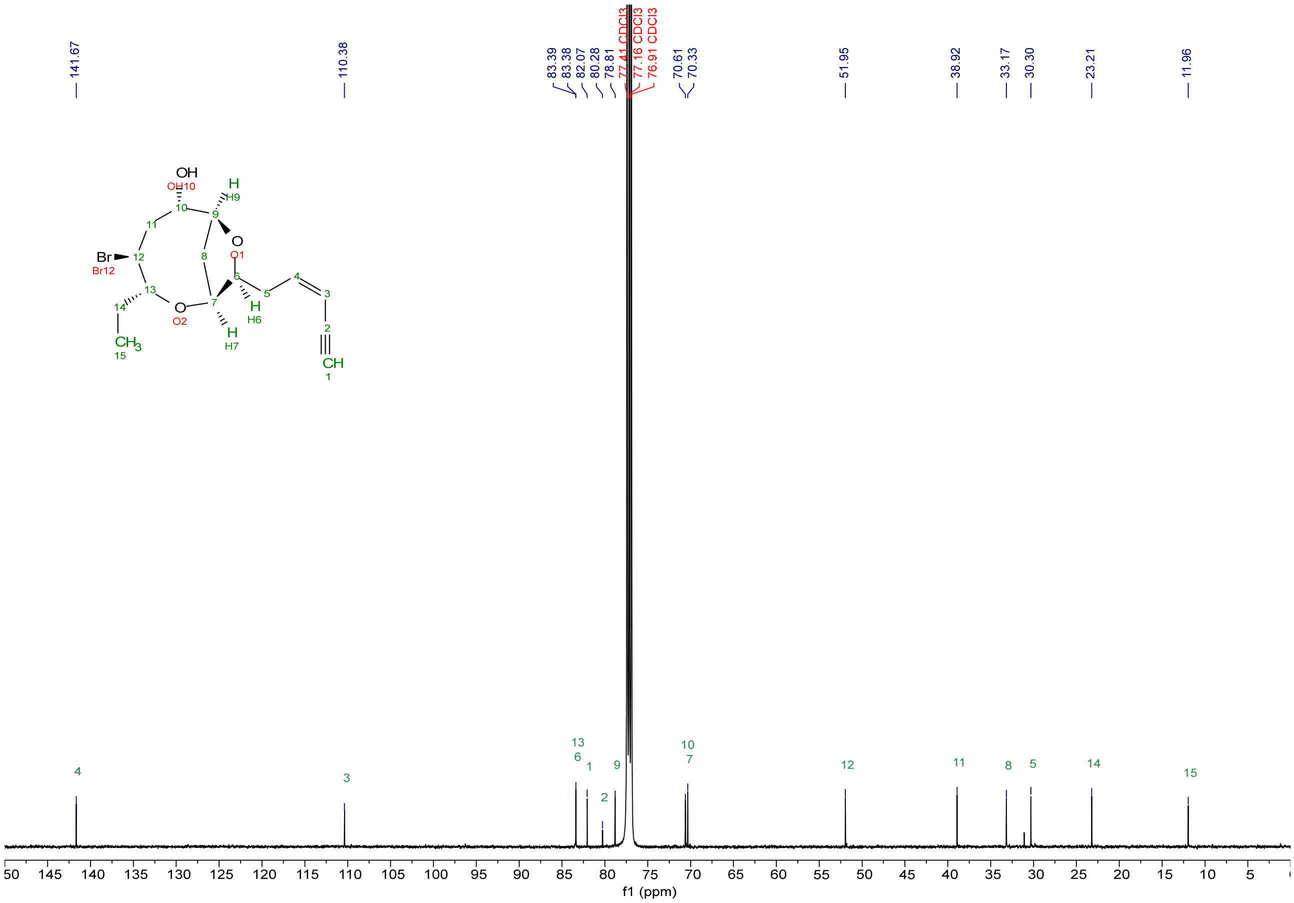

(1*S*,3*R*,4*S*,6*S*,7*S*,9*S*)-4-Bromo-3-ethyl-9-((*Z*)-pent-2-en-4-yn-1-yl)-2,8-dioxabicyclo [5.2.1]decan-6-yl (*S*)-3,3,3-trifluoro-2-methoxy-2-phenylpropanoate S3 (500MHz, $CDCl_3$)

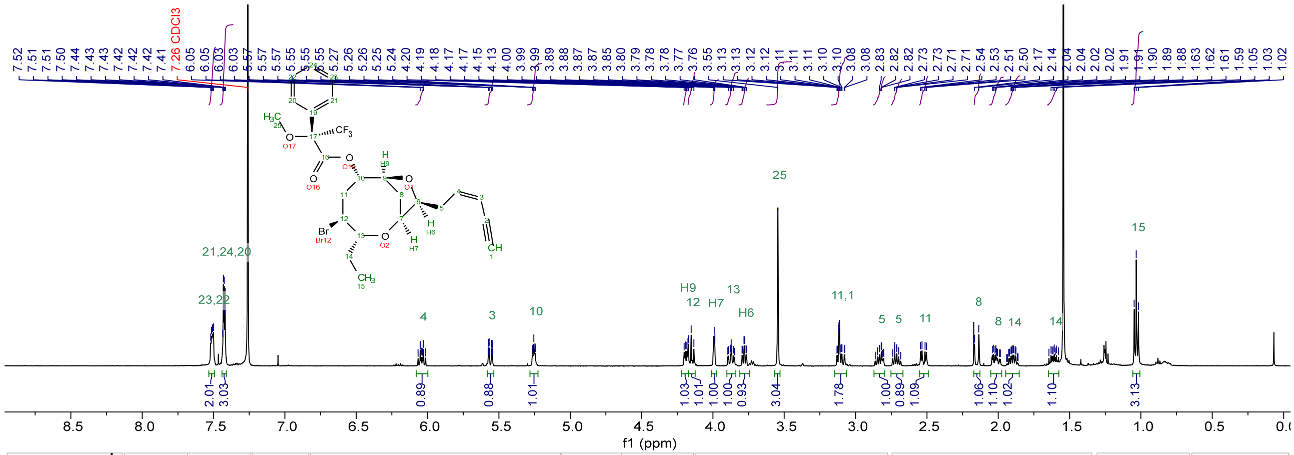

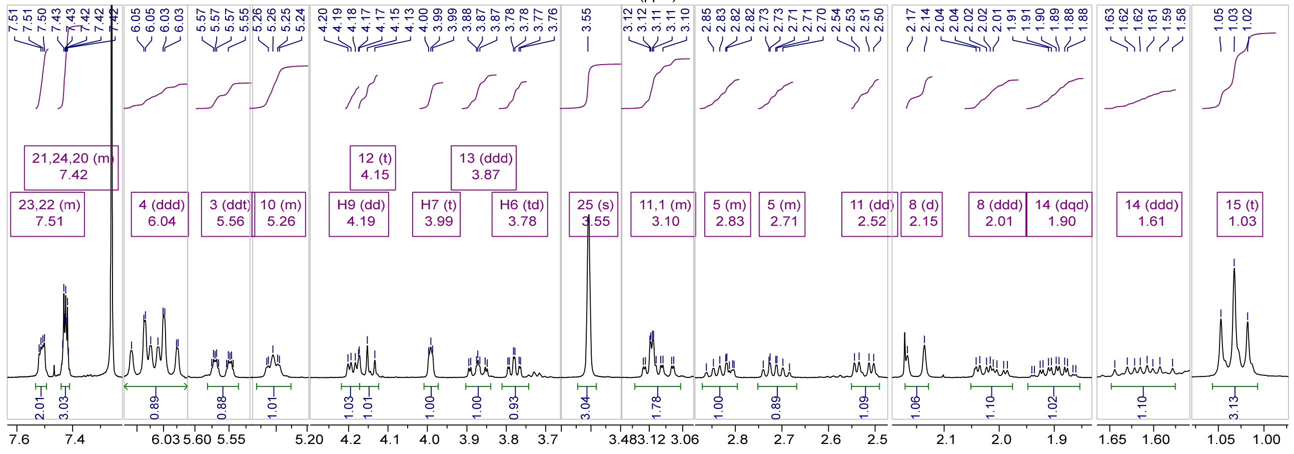

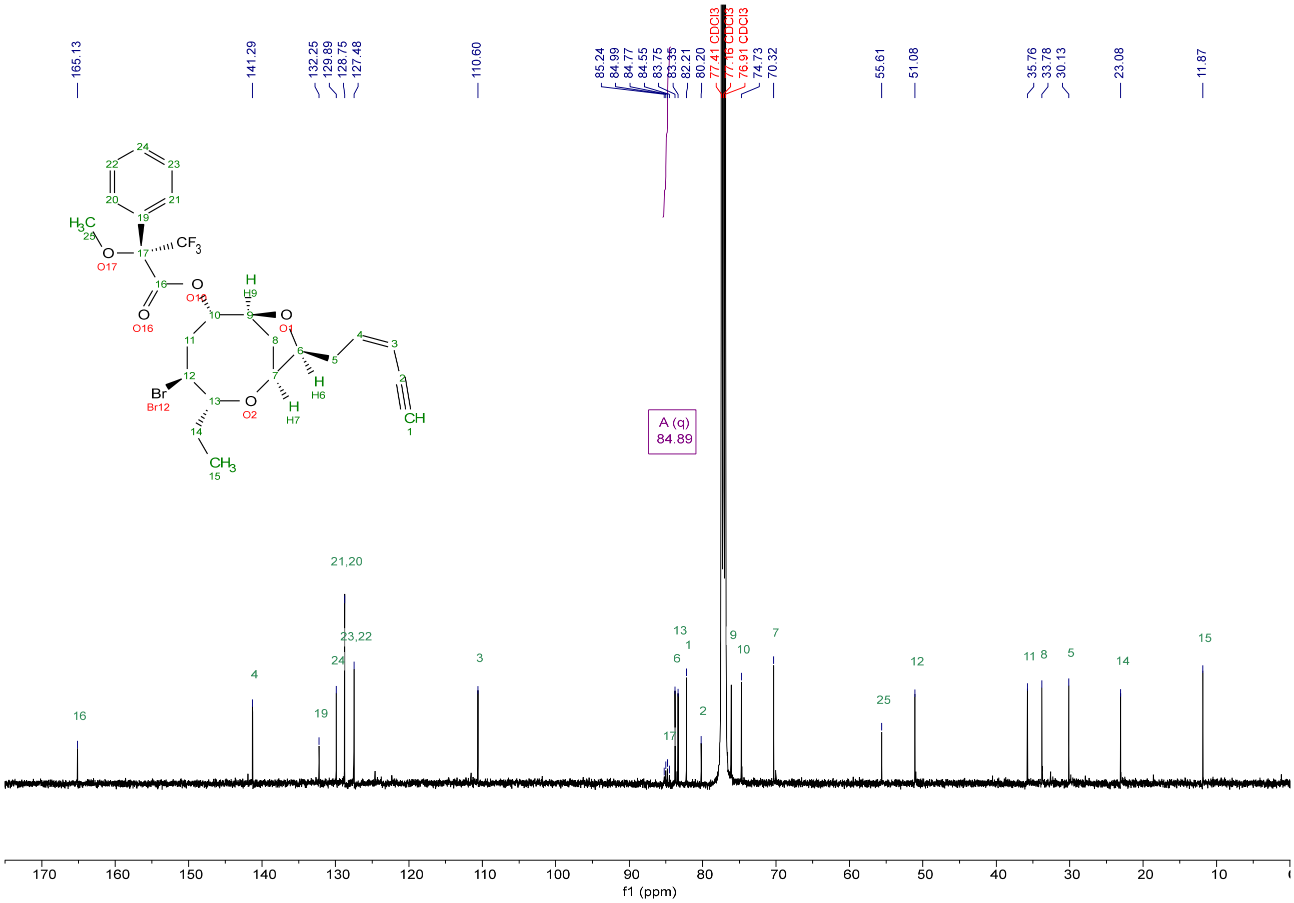

(1*S*,3*R*,4*S*,6*S*,7*S*,9*S*)-4-Bromo-3-ethyl-9-((*Z*)-pent-2-en-4-yn-1-yl)-2,8-dioxabicyclo [5.2.1]decan-6-yl (*R*)-3,3,3-trifluoro-2-methoxy-2-phenylpropanoate S4 (500MHz, $CDCl_3$)

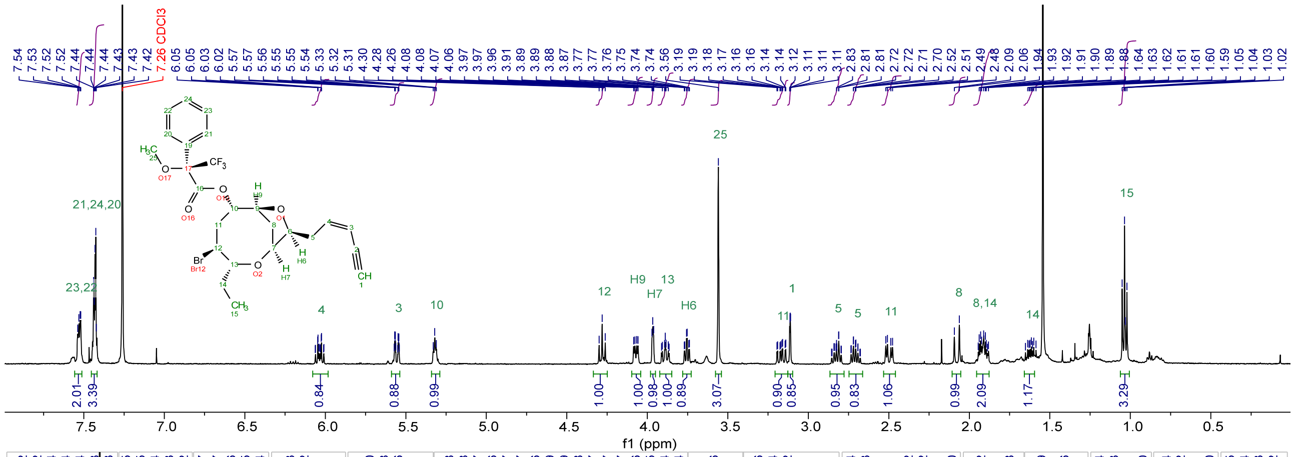

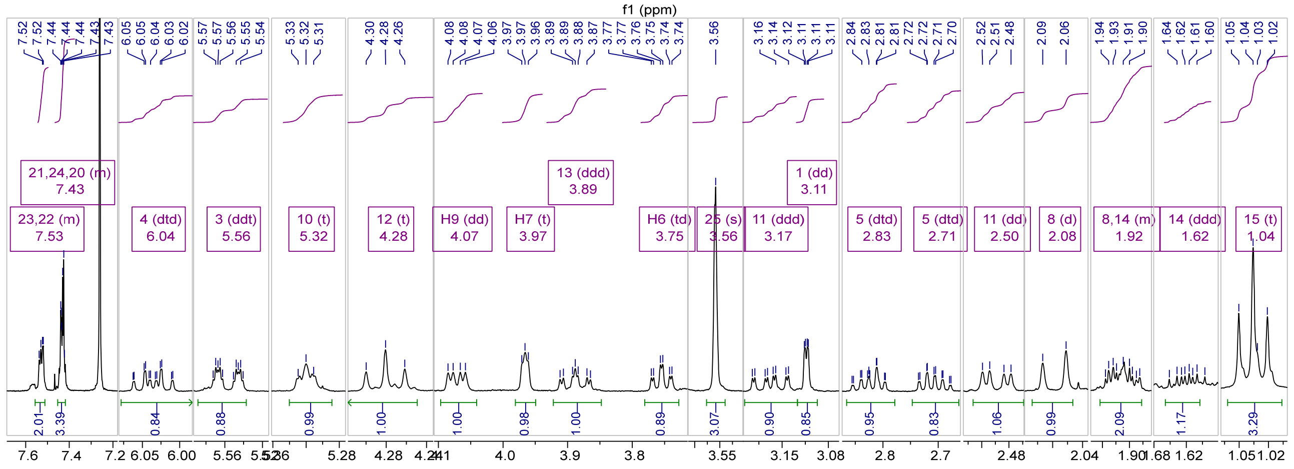

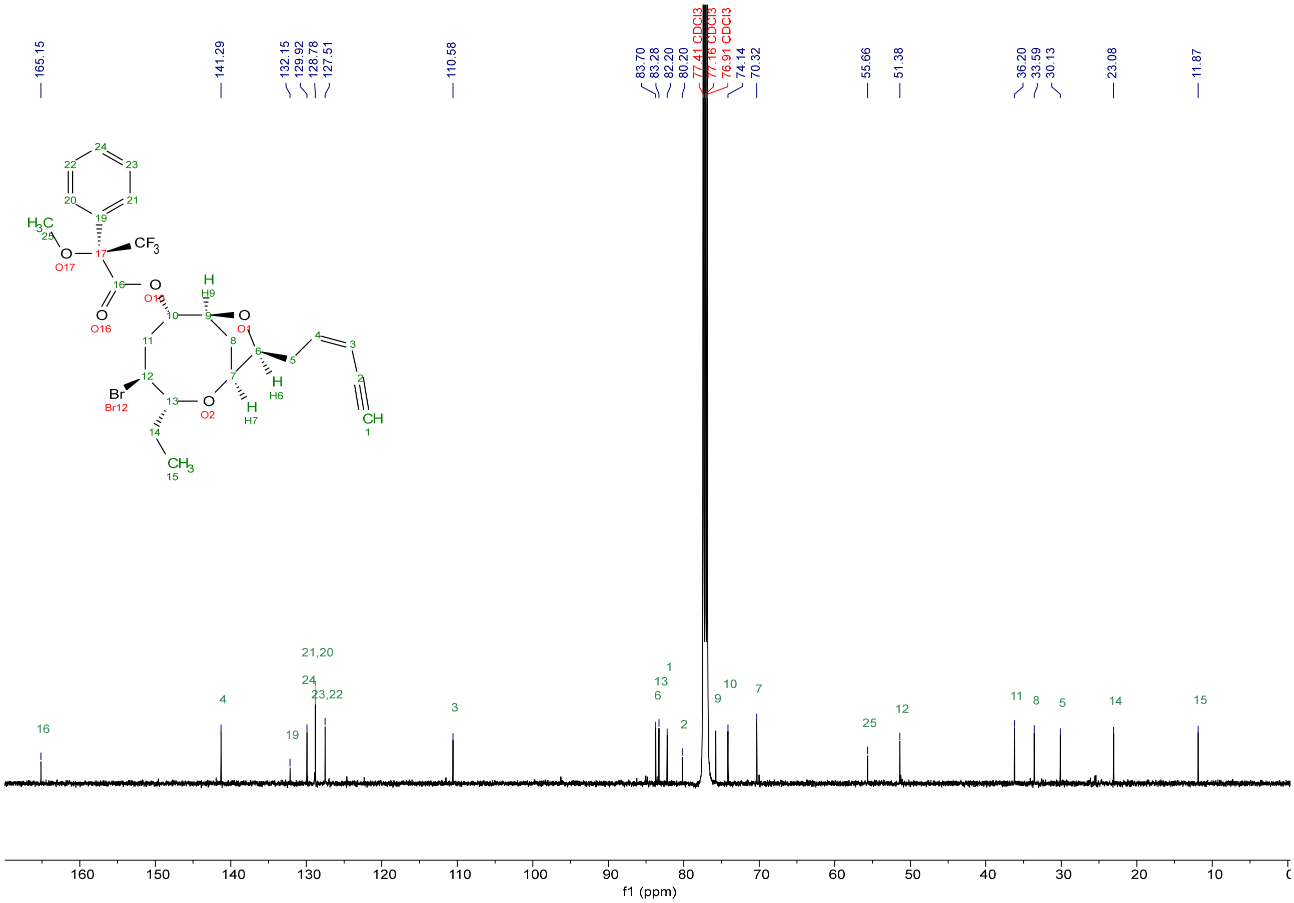

(1*S*,3*R*,6*S*,7*S*,9*S*,*Z*)-9-Allyl-3-ethyl-2,8-dioxabicyclo[5.2.1]dec-4-en-6-ol 21 (500MHz, $CDCl_3$)

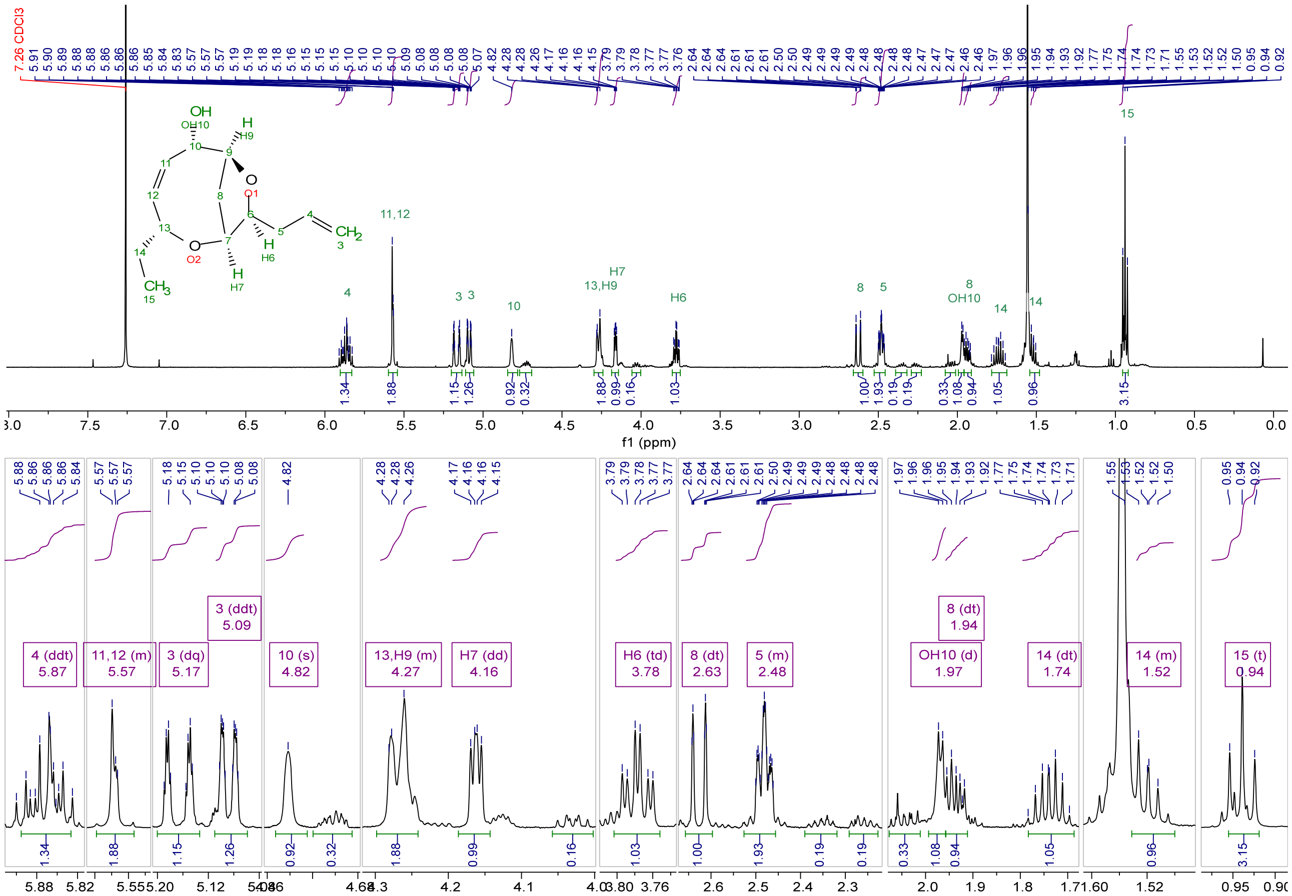

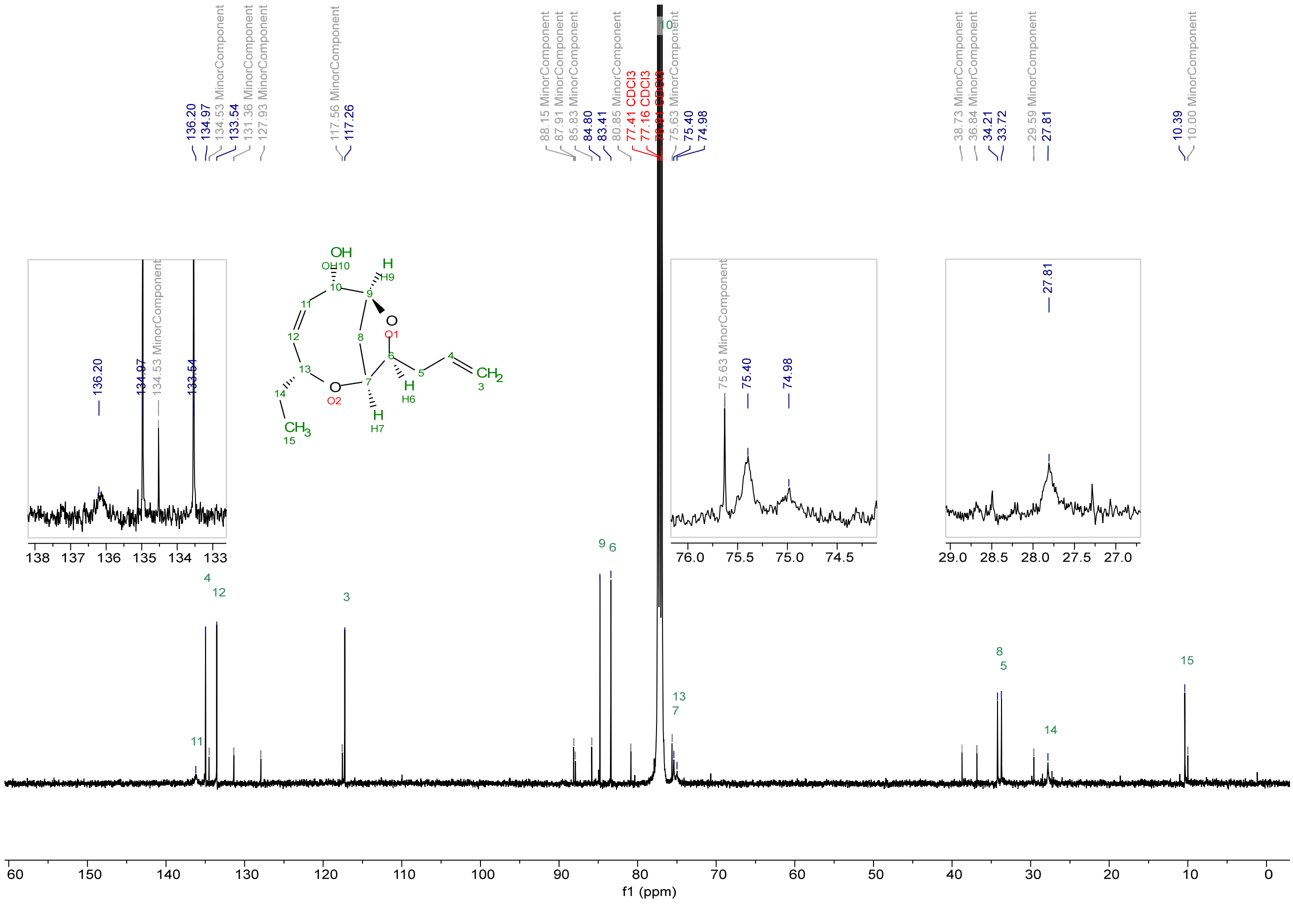

(1*S*,3*R*,4*R*,6*S*,7*S*,9*S*)-9-Allyl-3-ethyl-2,8-dioxabicyclo[5.2.1]decane-4,6-diol 22 (500MHz, $CDCl_3$)

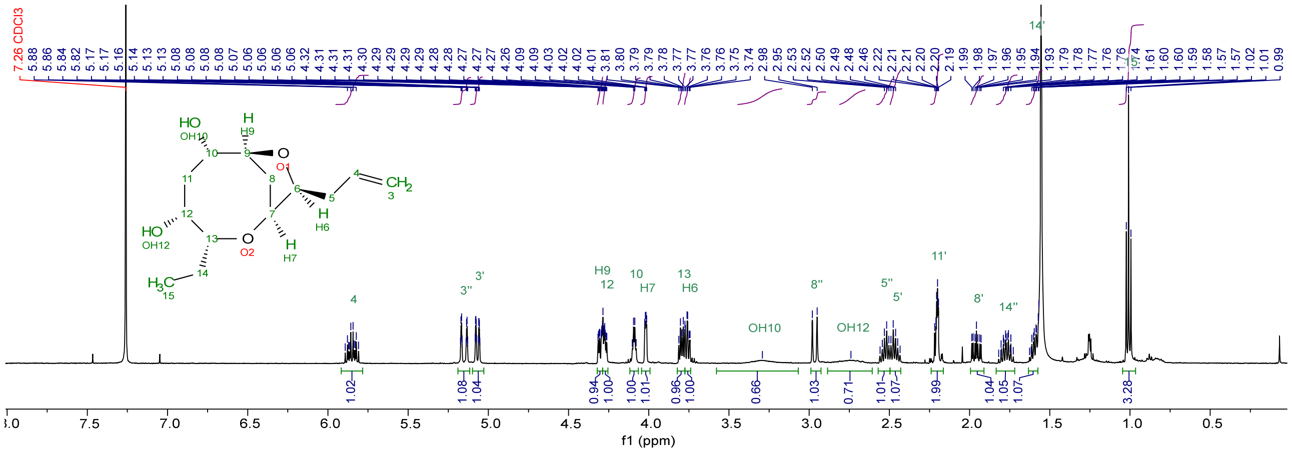

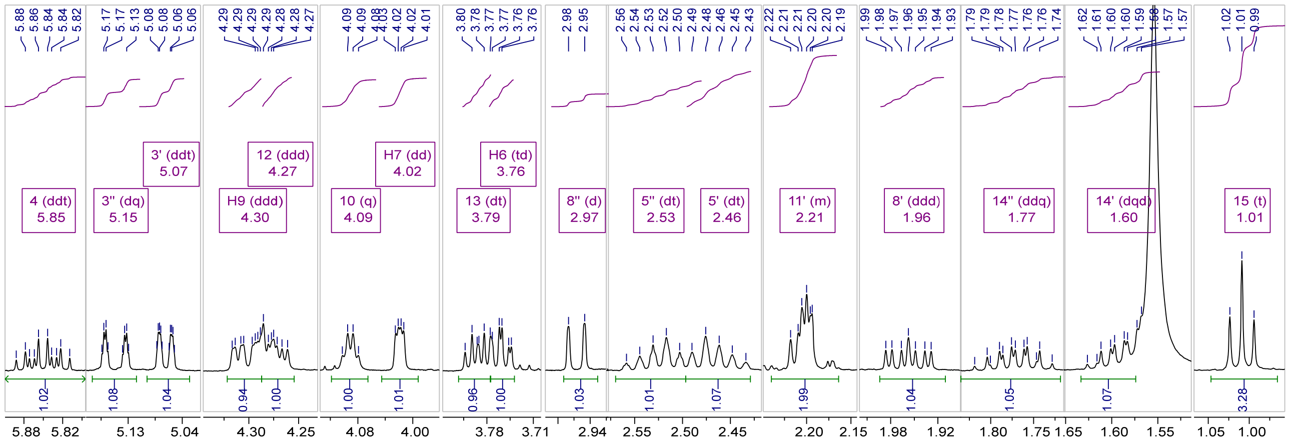

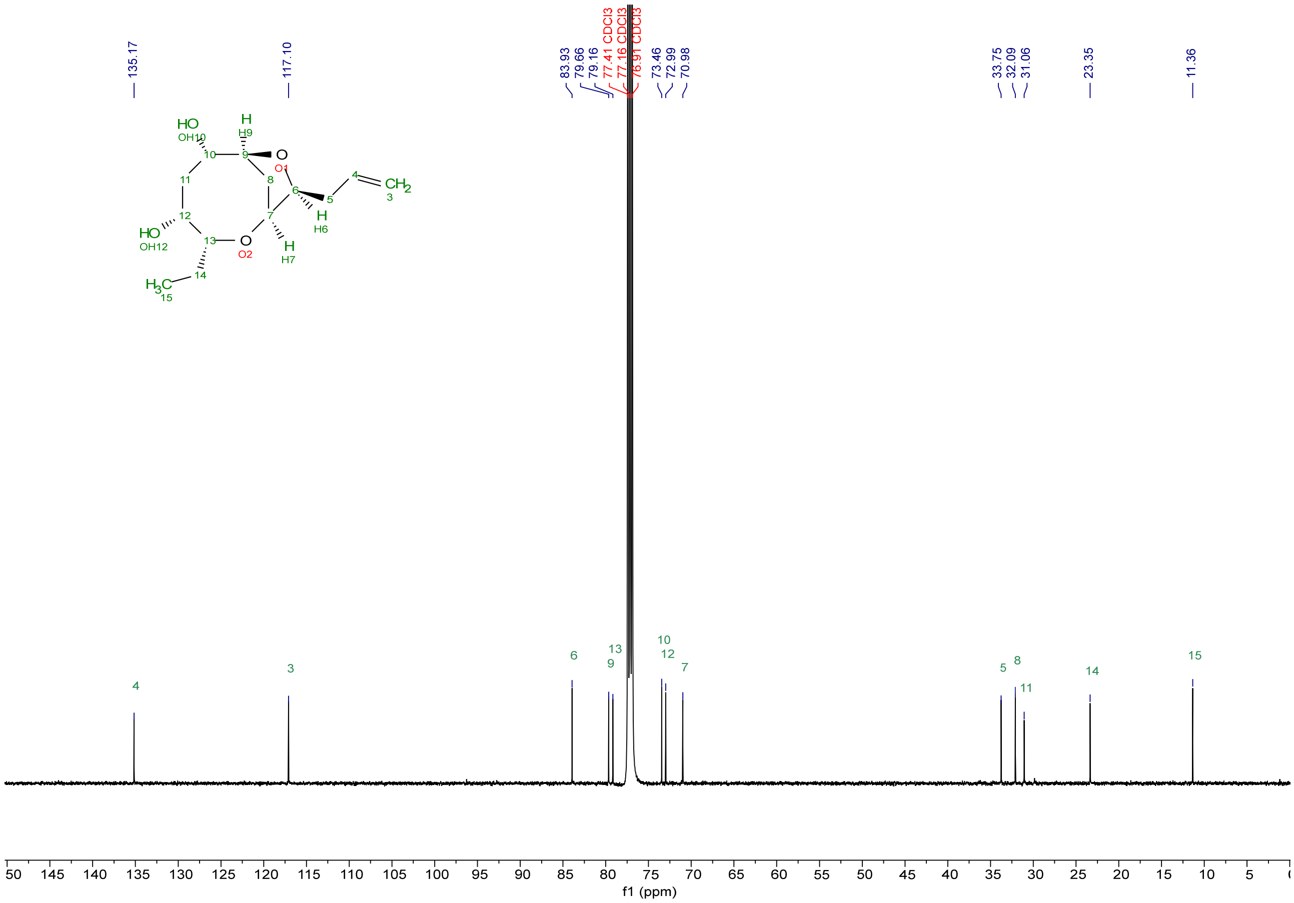

(1*S*,3*R*,4*R*,6*S*,7*S*,9*S*)-3-Ethyl-9-((*E*)-pent-2-en-4-yn-1-yl)-2,8-dioxabicyclo[5.2.1] decane-4,6-diol laurefurenyne D (*E*)-10 (500MHz, $CDCl_3$)

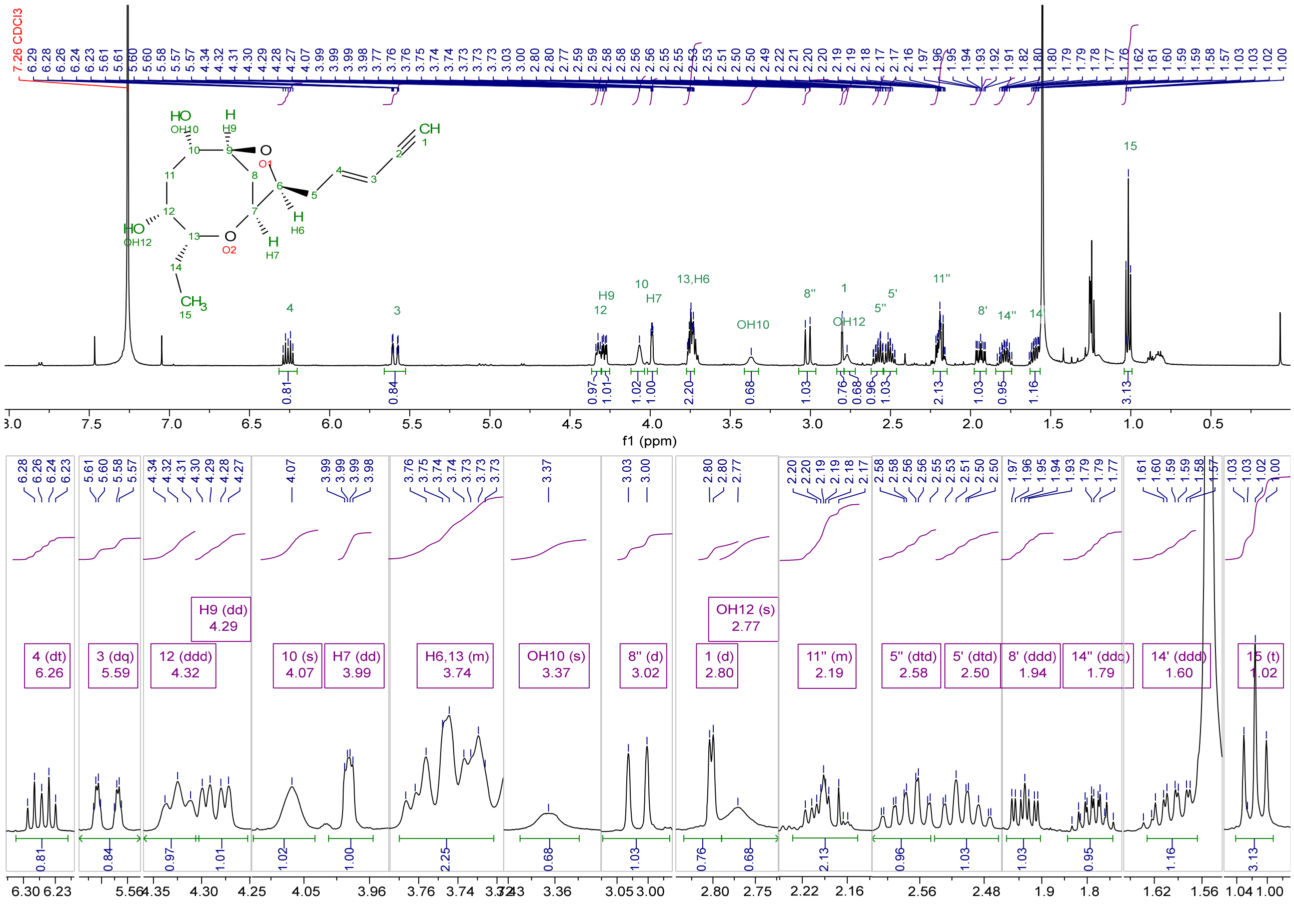

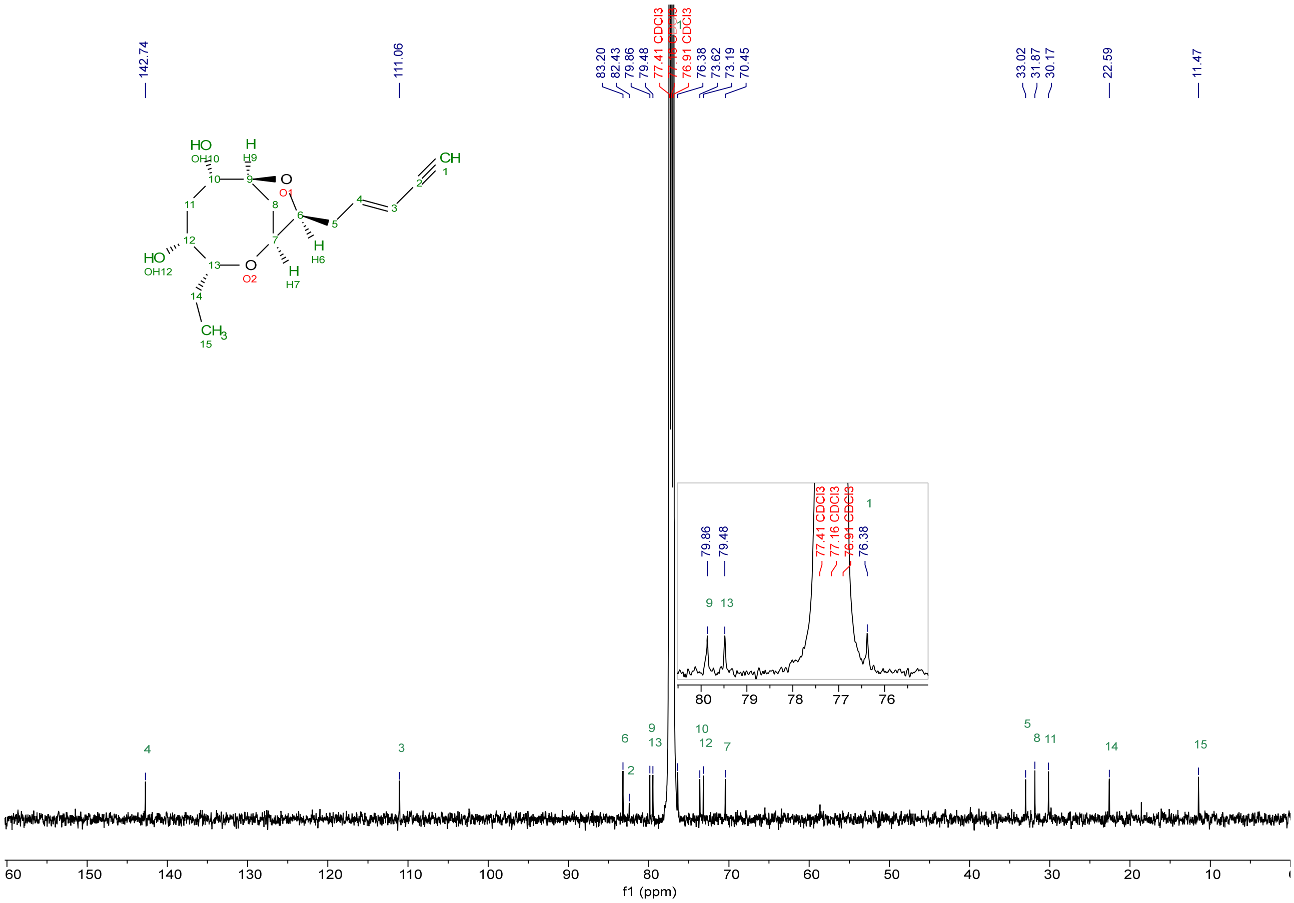

(1*S*,3*R*,4*R*,6*S*,7*S*,9*S*)-3-Ethyl-9-((*Z*)-pent-2-en-4-yn-1-yl)-2,8-dioxabicyclo[5.2.1] decane-4,6-diol laurefurenyne C (*Z*)-10 (500MHz, $CDCl_3$)

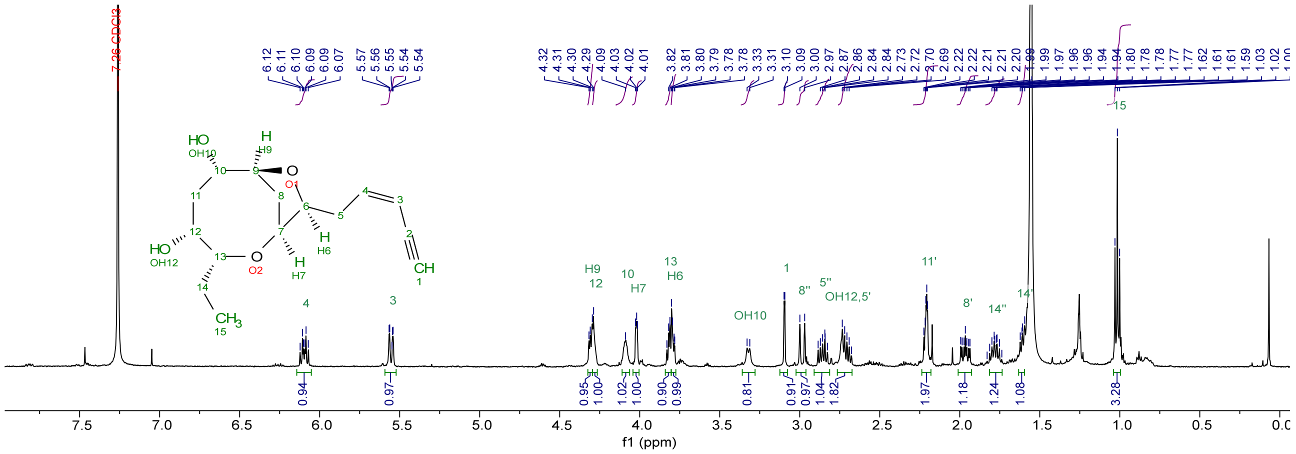

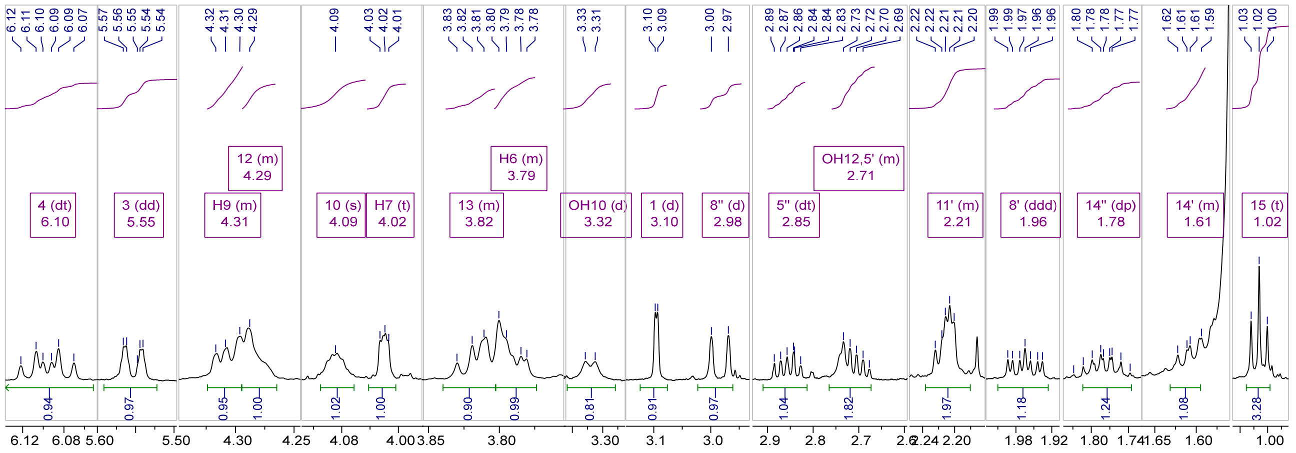

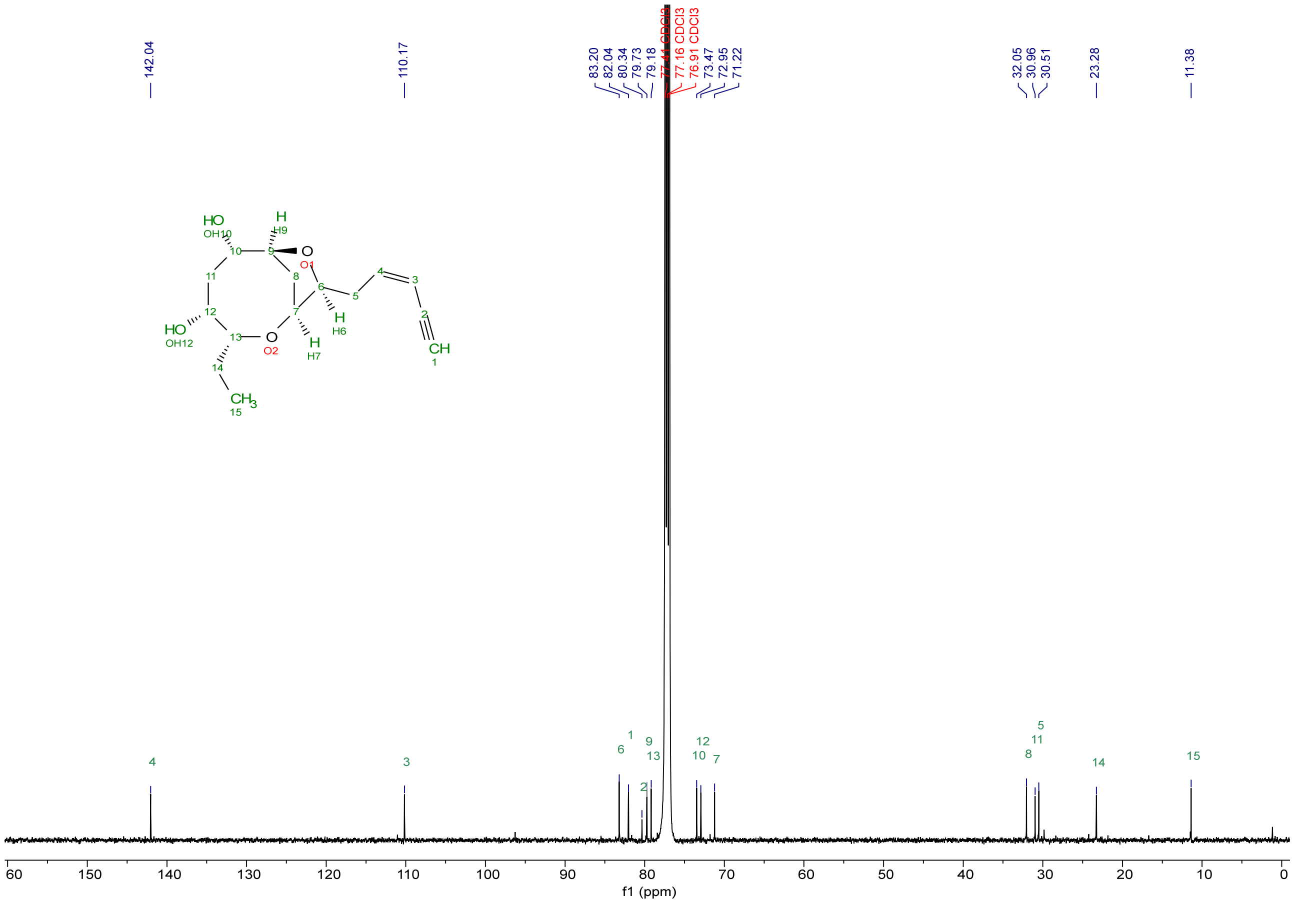

5-(((1*S*,3*S*,4*R*,6*S*,7*S*,9*S*)-9-Allyl-4-bromo-3-ethyl-2,8-dioxabicyclo[5.2.1]decan-6-yl)thio)-1-phenyl-1*H*-tetrazole S5 (500MHz, $CDCl_3$)

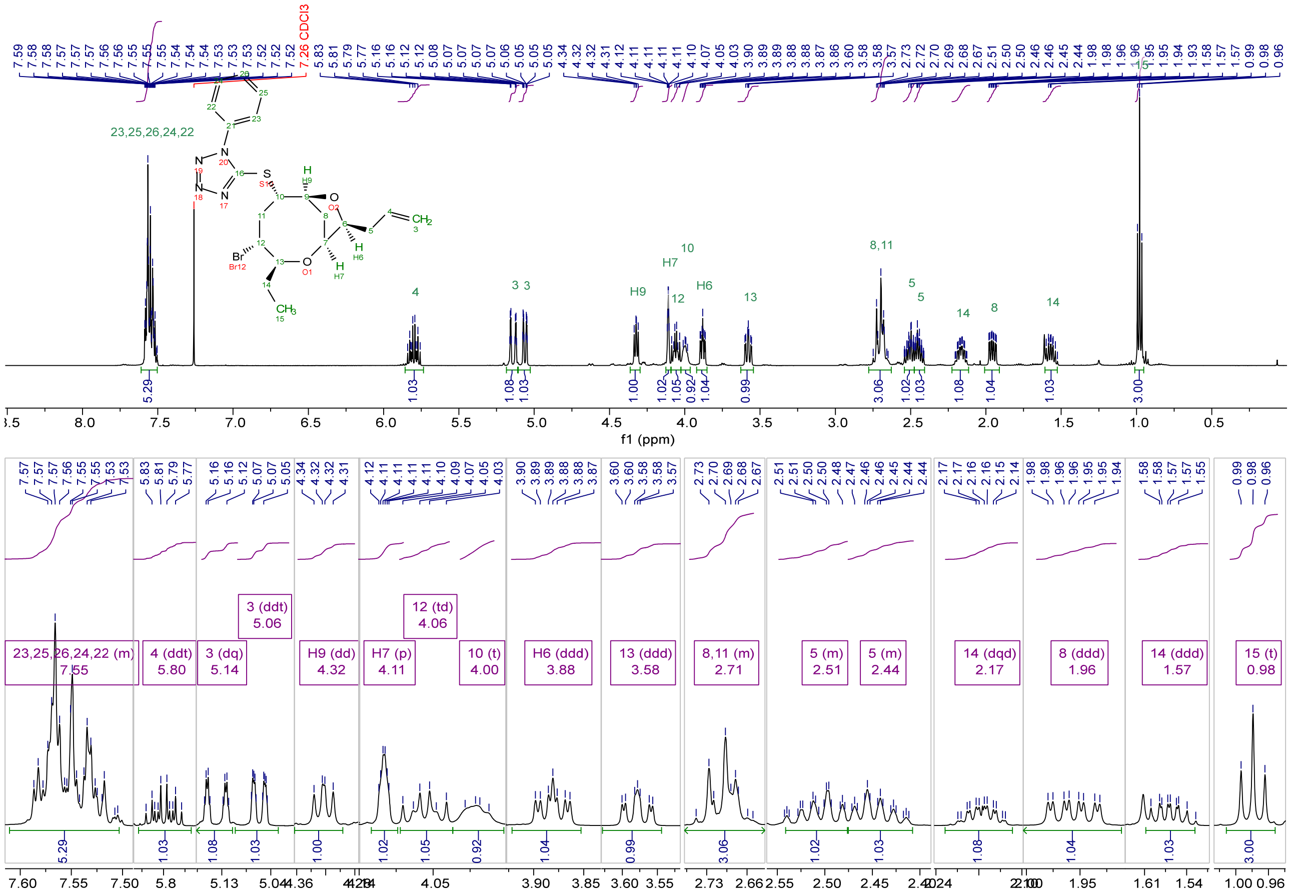

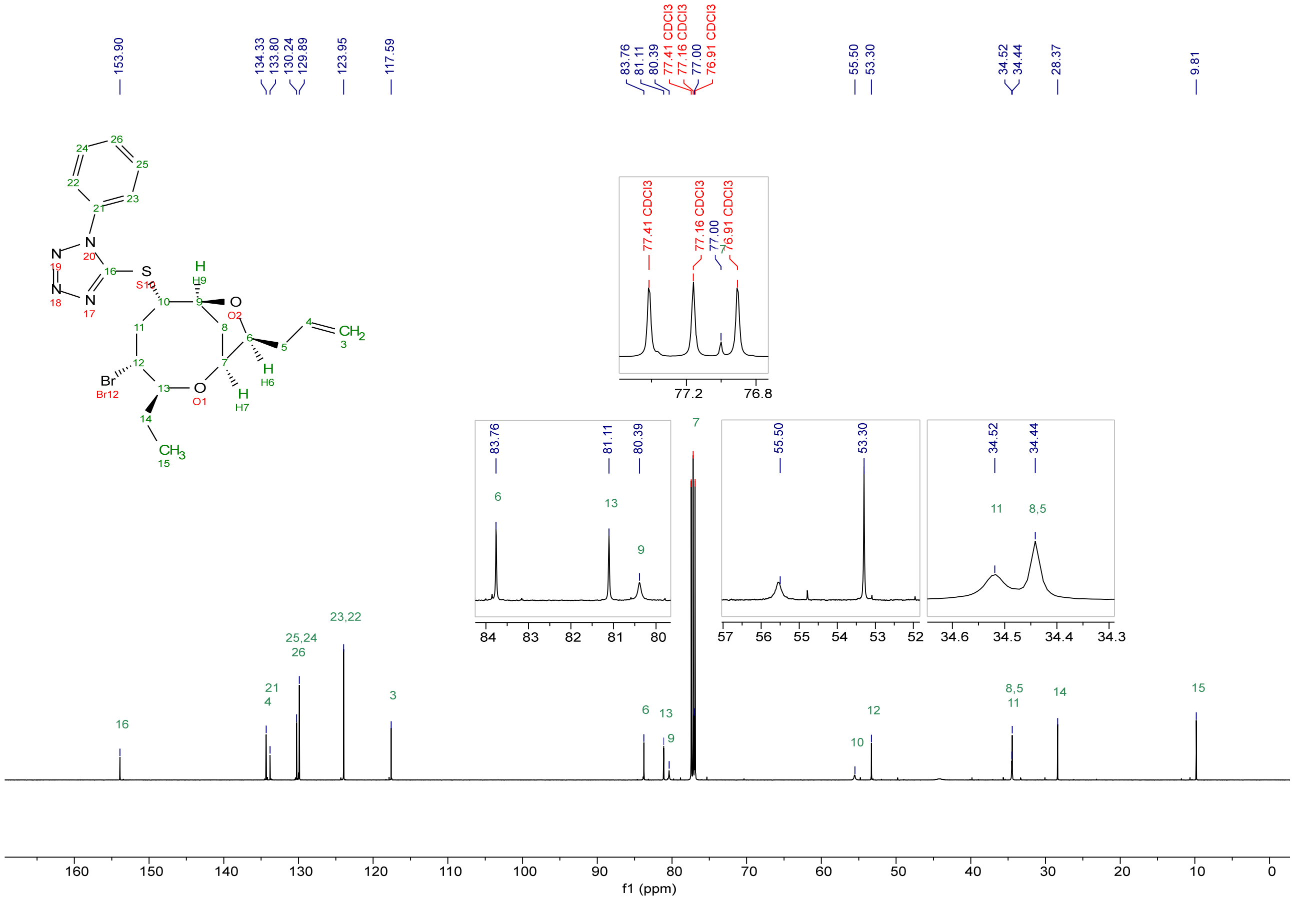

5-(((1*S*,3*S*,4*R*,6*S*,7*S*,9*S*)-9-Allyl-4-bromo-3-ethyl-2,8-dioxabicyclo[5.2.1]decan-6-yl)sulfonyl)-1-phenyl-1*H*-tetrazole 30 (500MHz, $CDCl_3$)

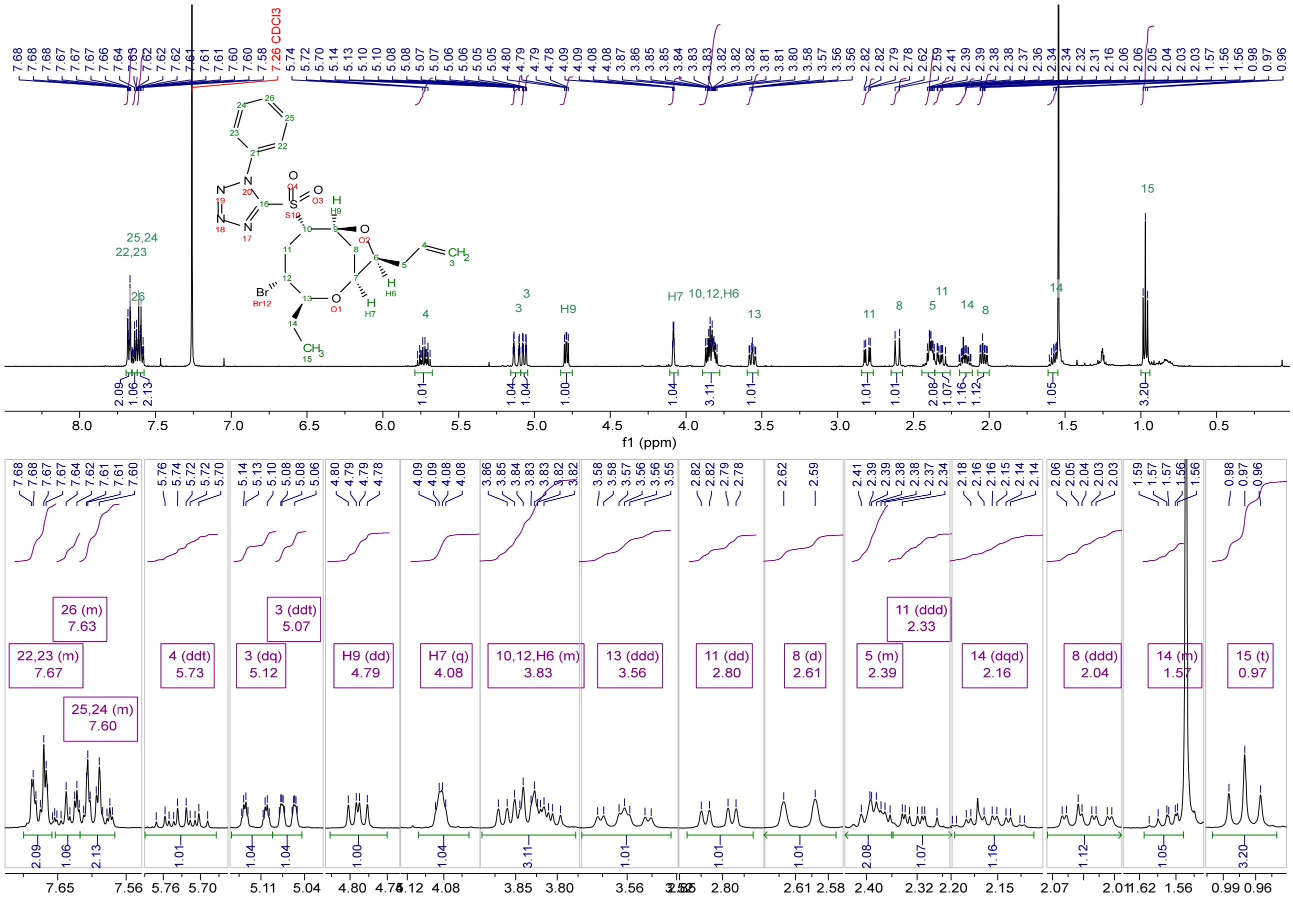

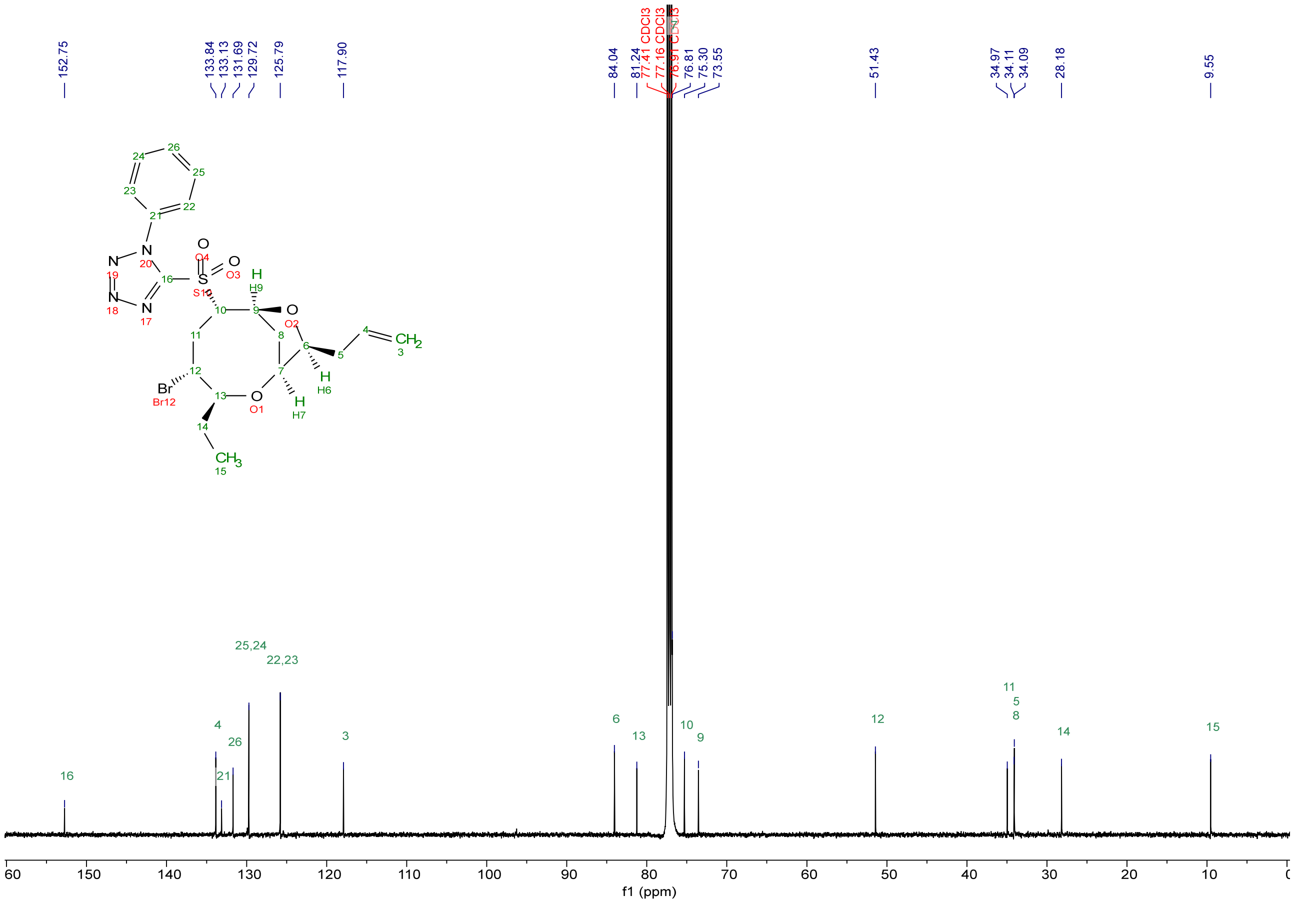

5-(((1*S*,2*R*,4*S*,5*R*,7*S*,8*S*)-8-Allyl-5-ethyl-6,9-dioxatricyclo[5.2.1.0^{2,4}]decan-2-yl)sulfonyl)-1-phenyl-1*H*-tetrazole 32 (500MHz, CDCl$_3$)

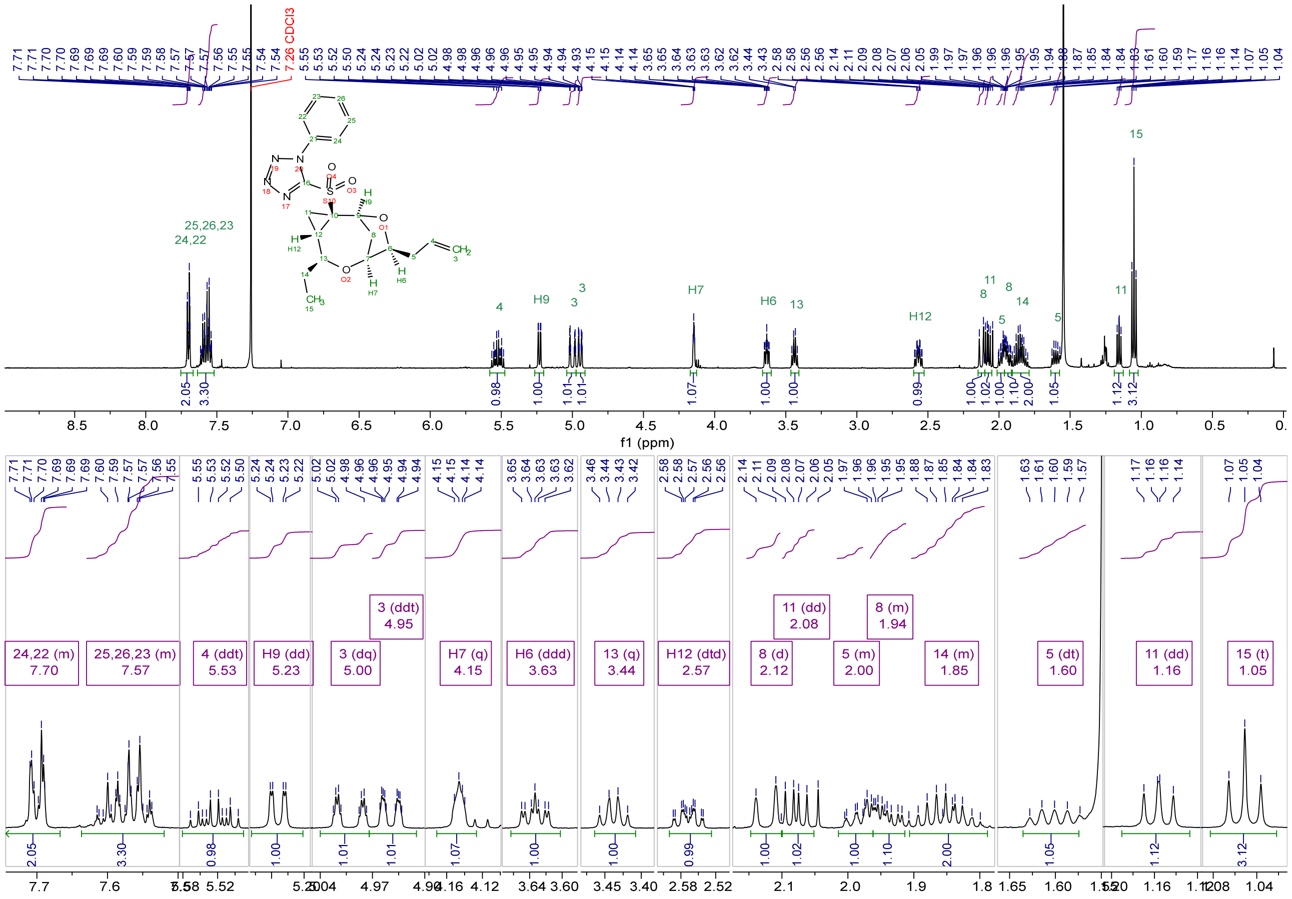

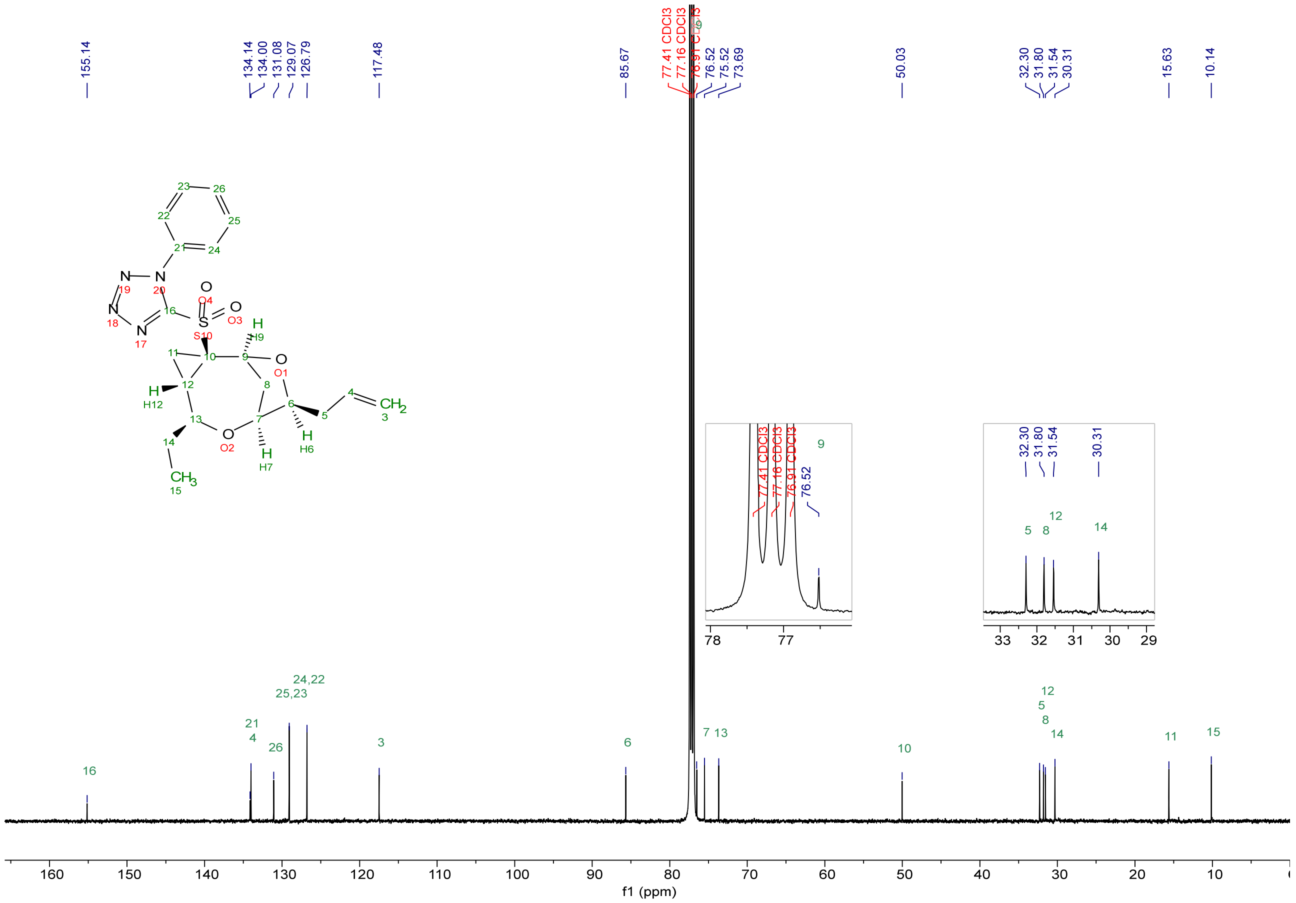

(*S*)-1-((1*S*,2*S*,4*S*,7*S*)-2-Ethyl-7-((1-phenyl-1*H*-tetrazol-5-yl)sulfonyl)-3-oxabicyclo[5.1.0] oct-5-en-4-yl)but-3-en-1-ol 33 (500MHz, $CDCl_3$)

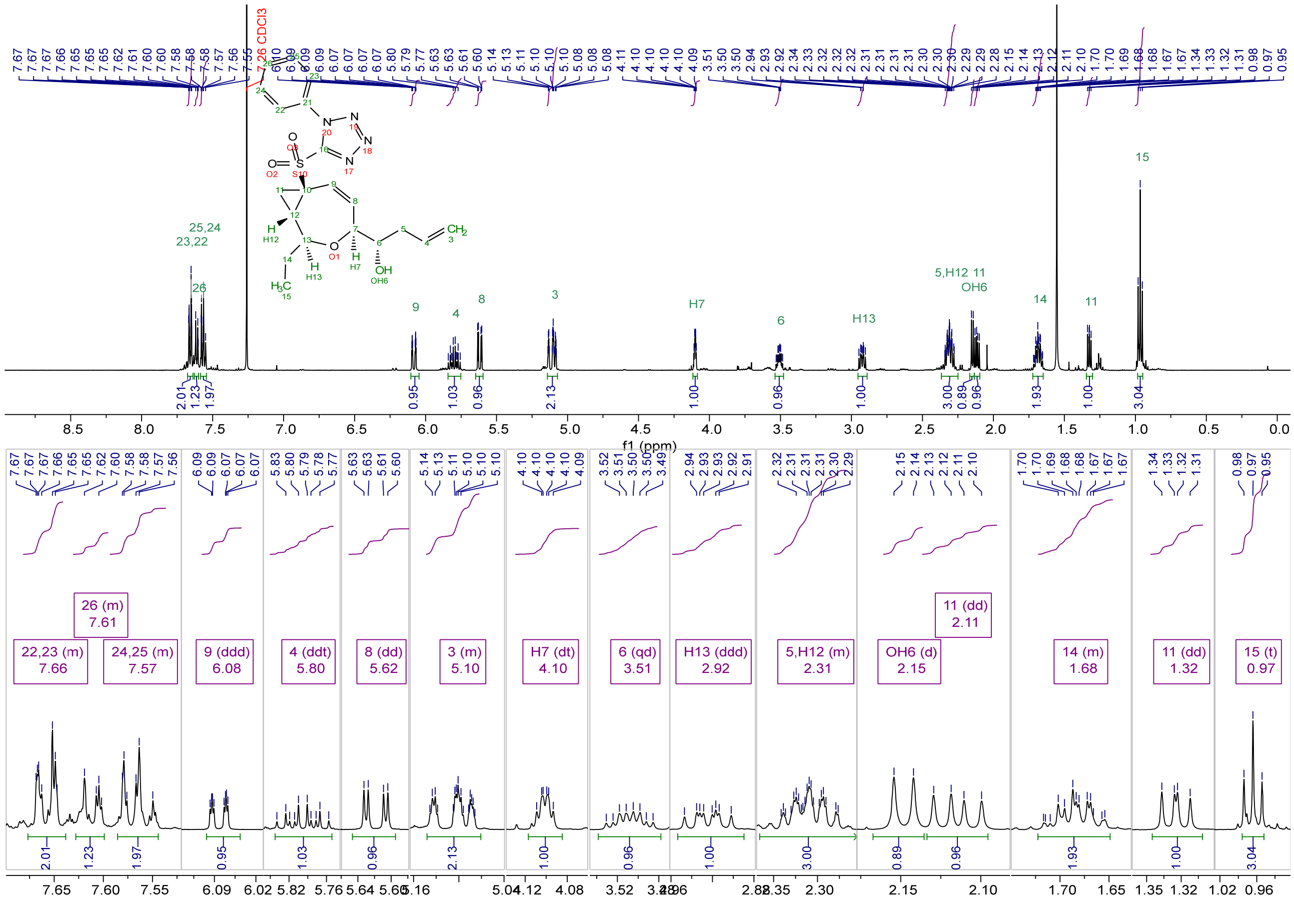

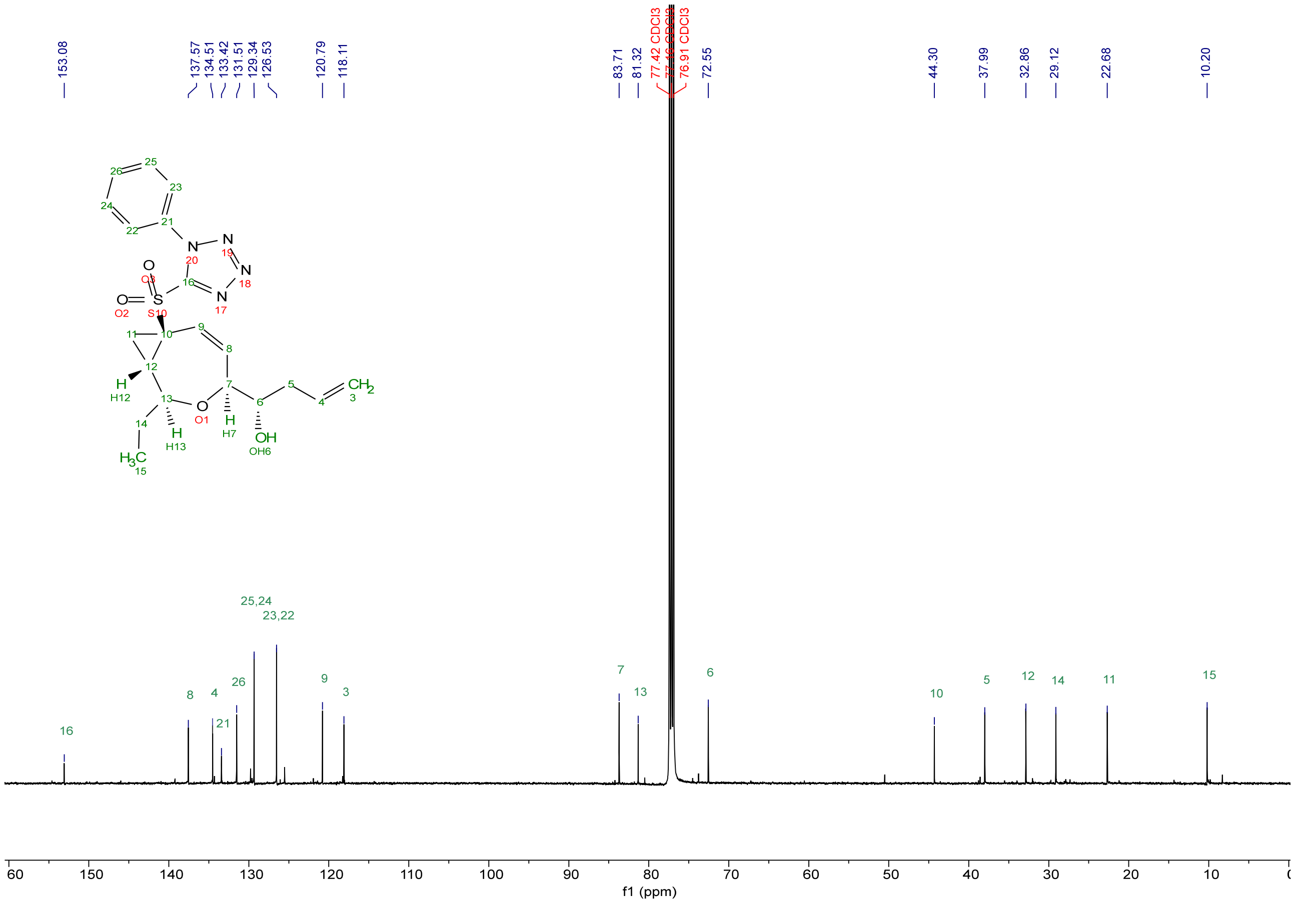

(1*S*,3*S*,4*R*,7*S*,9*S*)-9-Allyl-4-bromo-3-ethyl-2,8-dioxabicyclo[5.2.1]decan-6-one 37 (500MHz, $CDCl_3$)

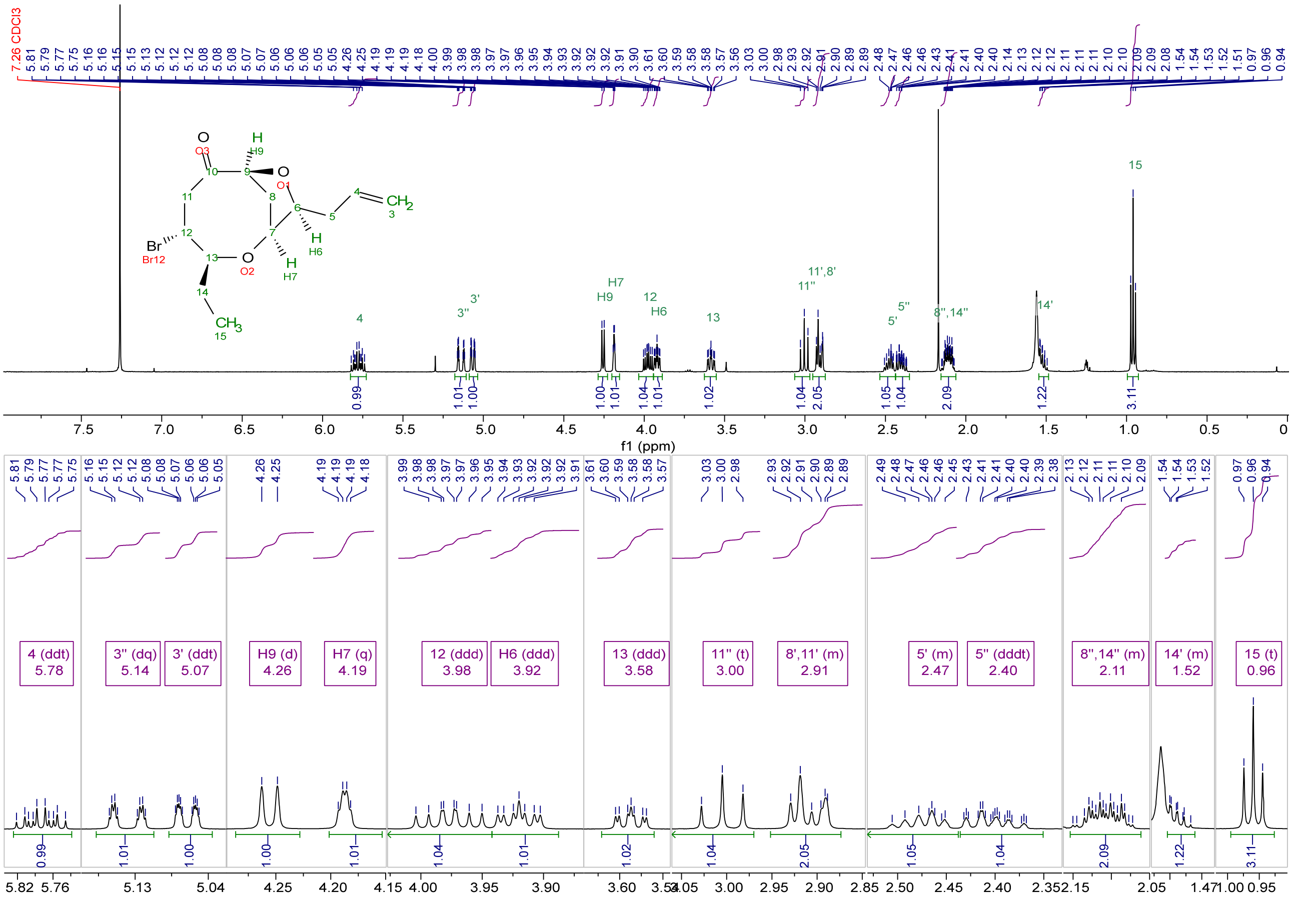

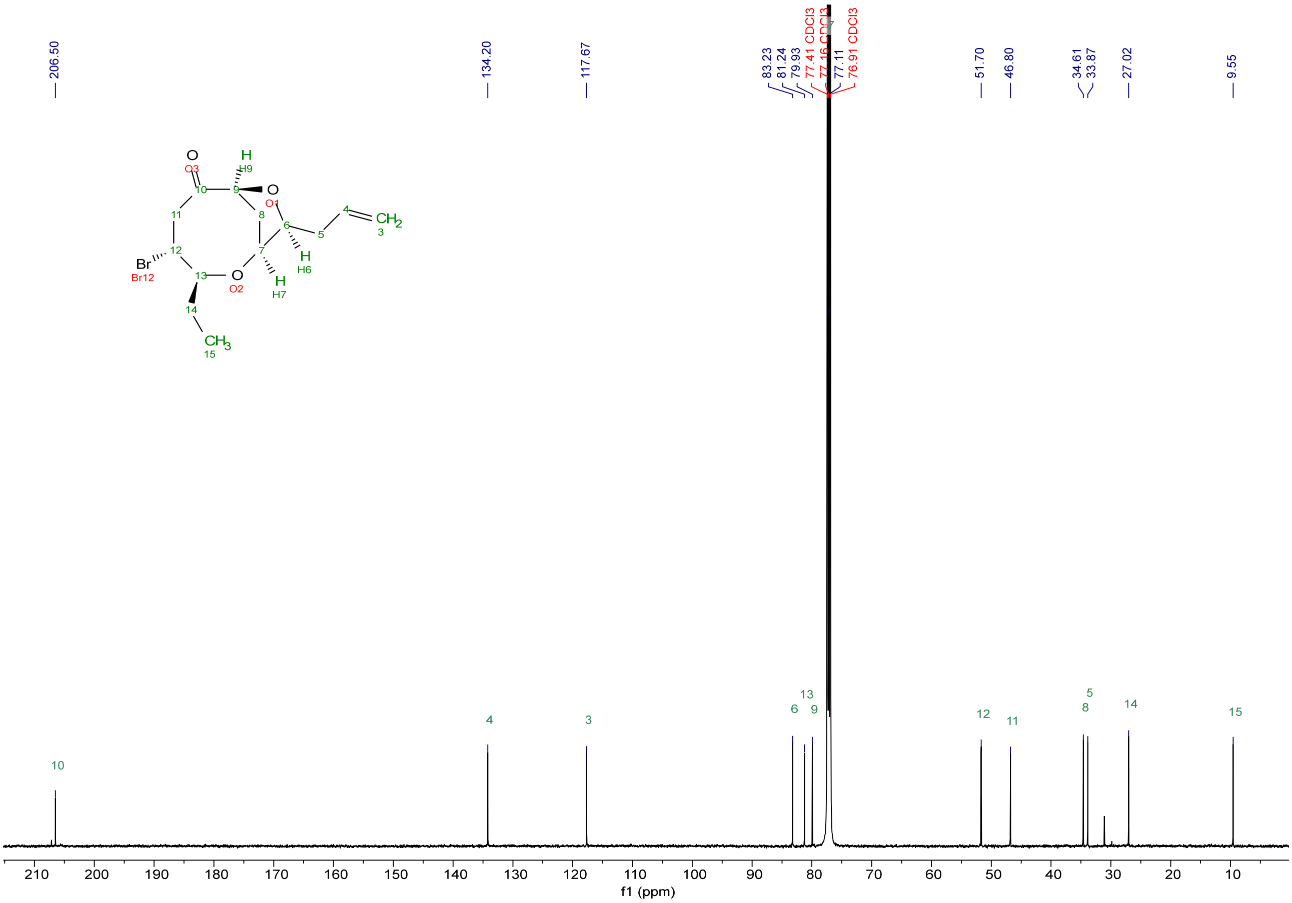

(1*S*,2*S*,4*R*,5*S*,7*S*,8*S*)-8-Allyl-5-ethyl-6,9-dioxatricyclo[5.2.1.0^{2,4}]decane 39 and (*S*)-1-((2*S*,4*Z*,6*Z*,8*S*)-8-ethyl-3,8-dihydro-2*H*-oxocin-2-yl)but-3-en-1-ol 40 (500MHz, CDCl$_3$)

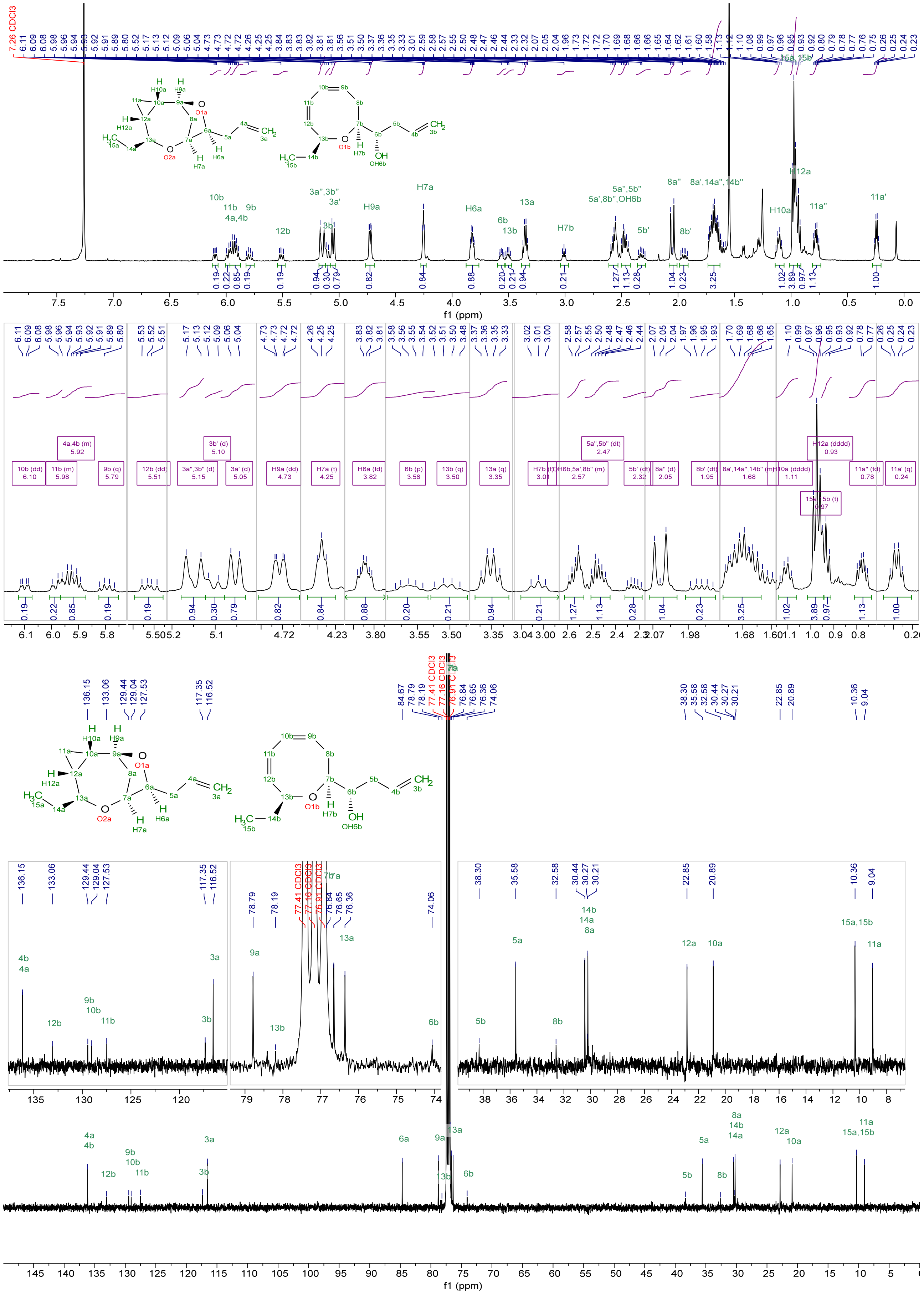

(*S*)-1-((2*S*,7*R*,8*S*,*Z*)-7-Bromo-8-ethyl-3,6,7,8-tetrahydro-2*H*-oxocin-2-yl)but-3-en-1-ol 41 (700MHz, $CDCl_3$)

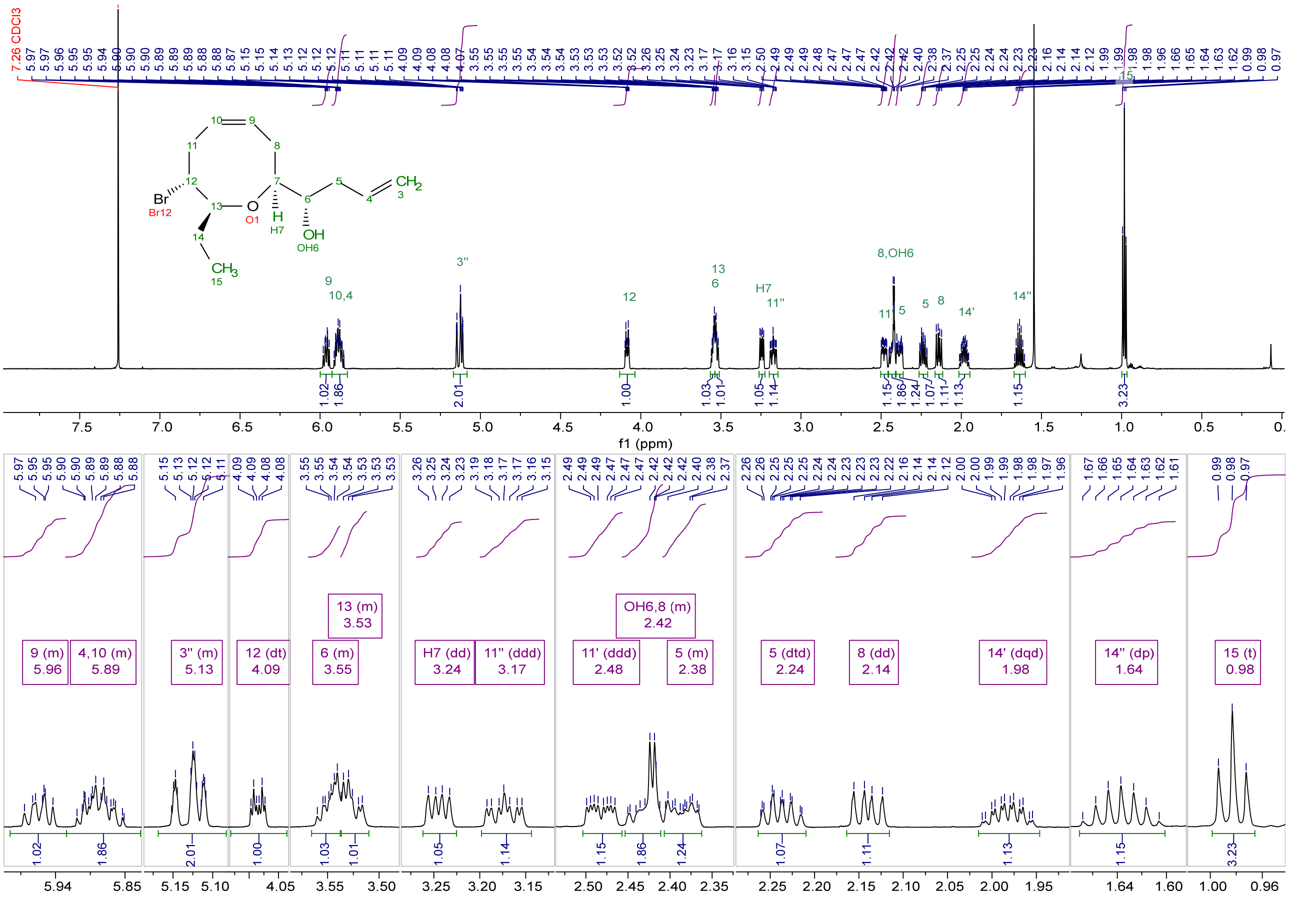

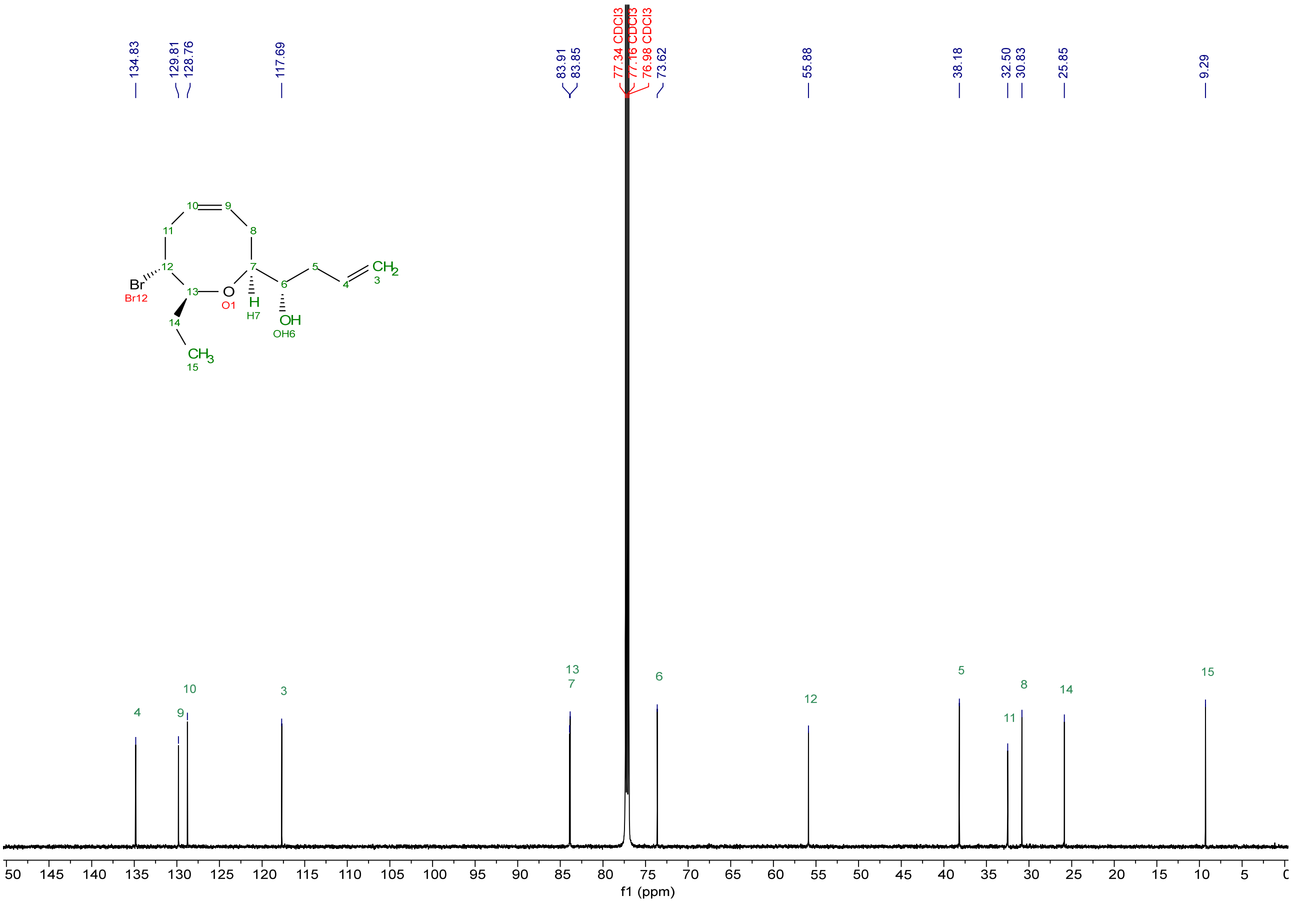

(1*S*,3*S*,4*R*,7*S*,9*S*)-4-Bromo-3-ethyl-9-((E)-pent-2-en-4-yn-1-yl)-2,8-dioxabicyclo [5.2.1]decan-6-one 42 (500MHz, $CDCl_3$)

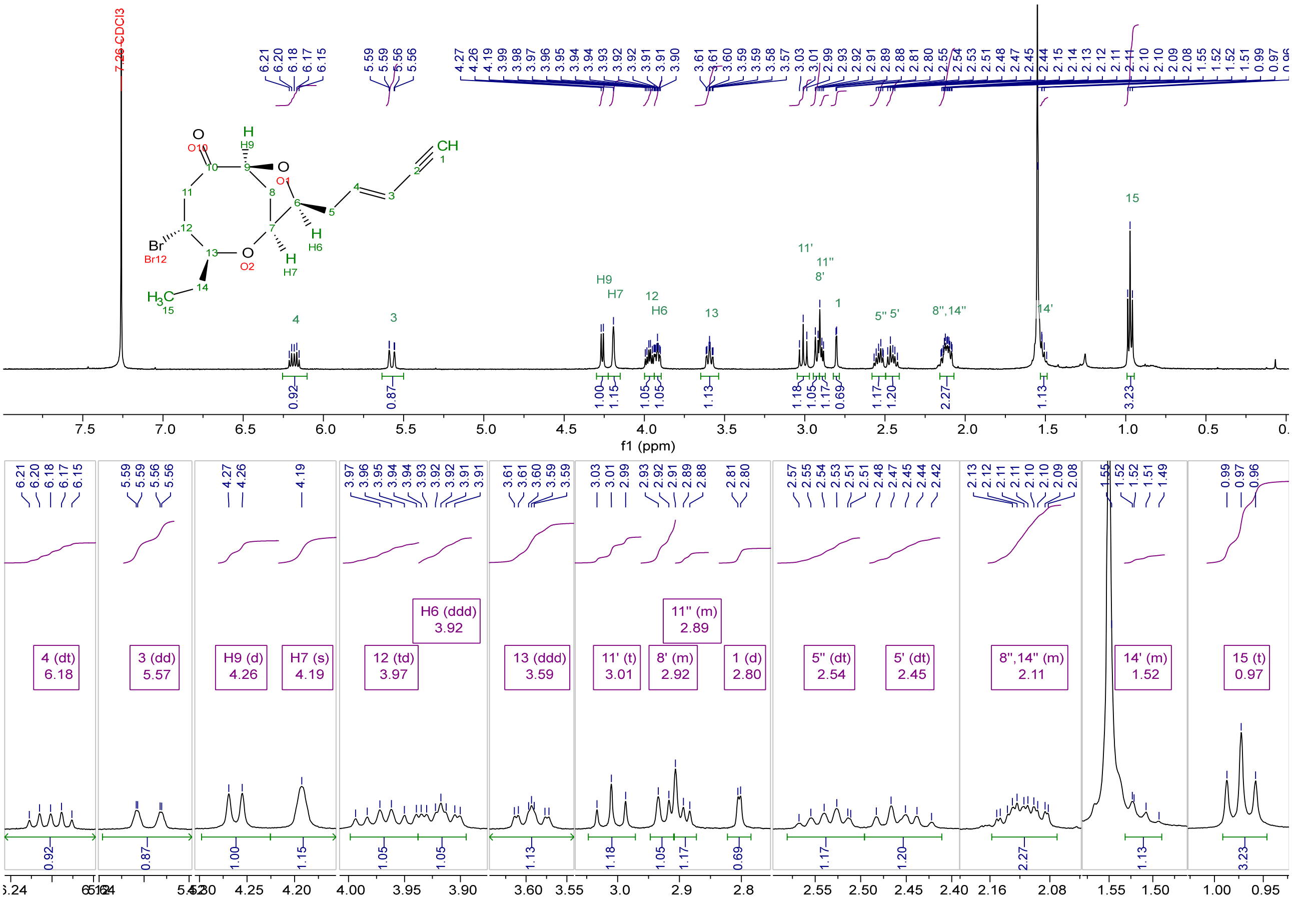

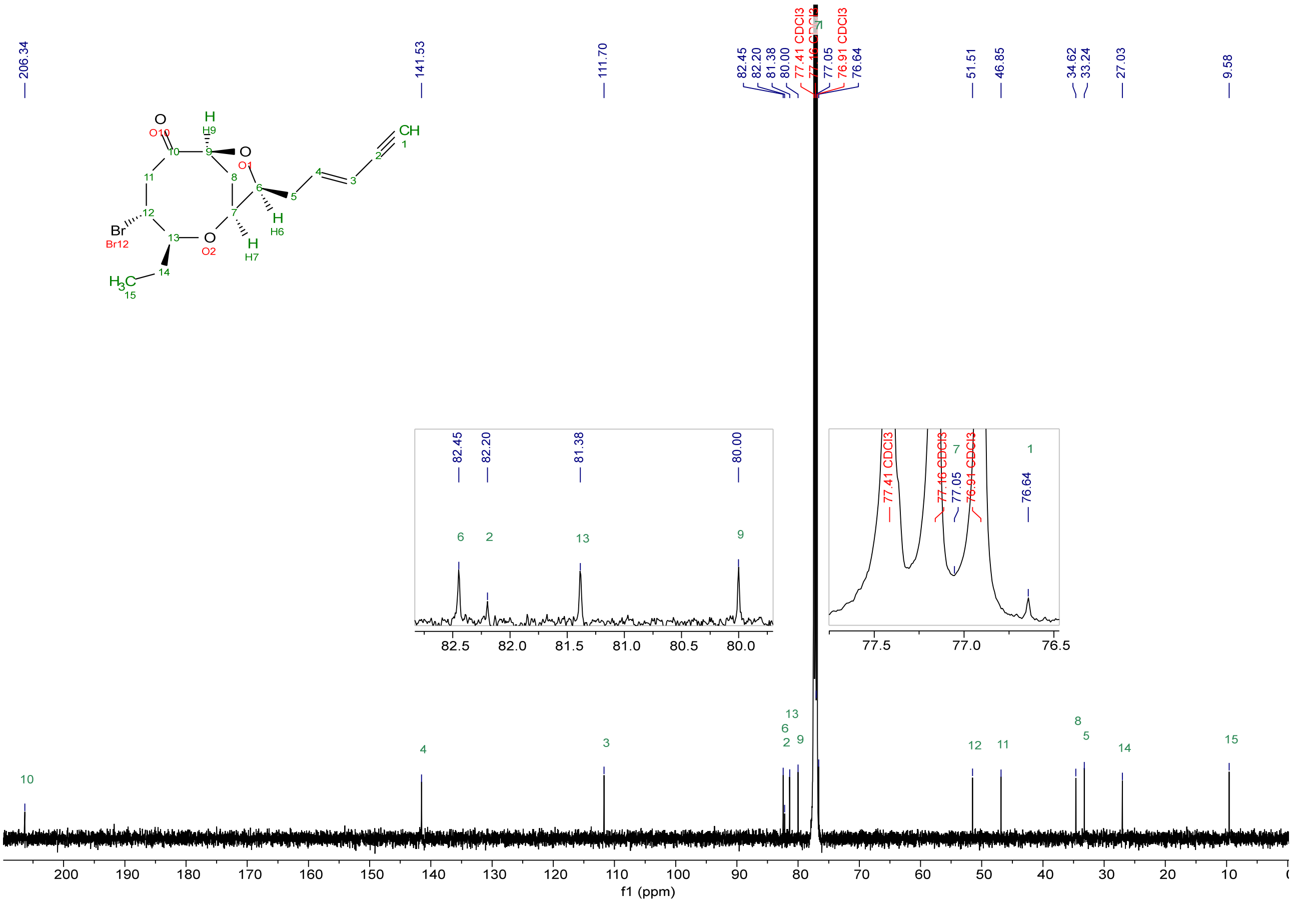

(*S*,*E*)-1-((2*S*,4*Z*,6*Z*,8*S*)-8-Ethyl-3,8-dihydro-2*H*-oxocin-2-yl)hex-3-en-5-yn-1-ol 43 (500MHz, $CDCl_3$)

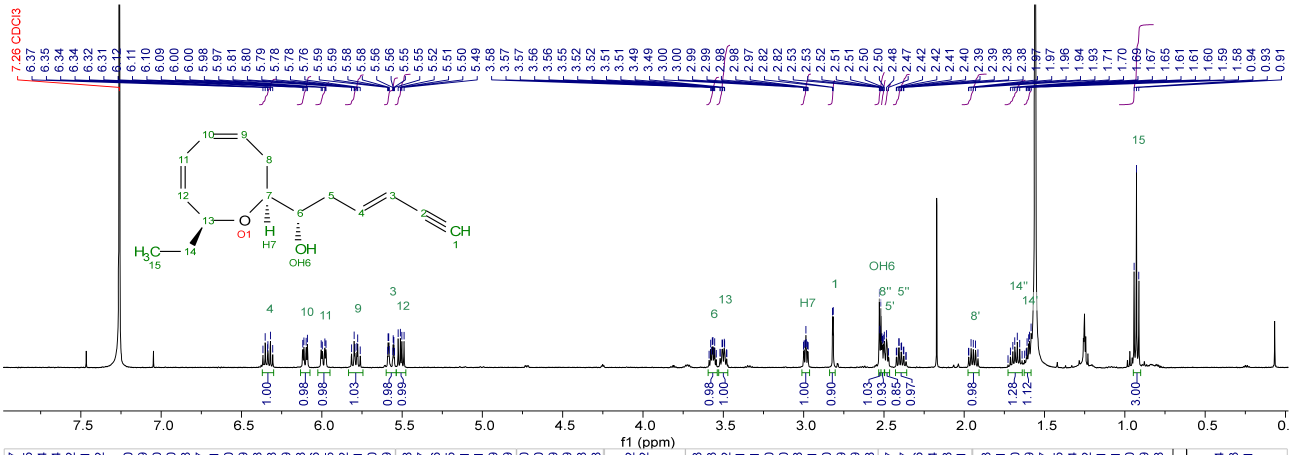

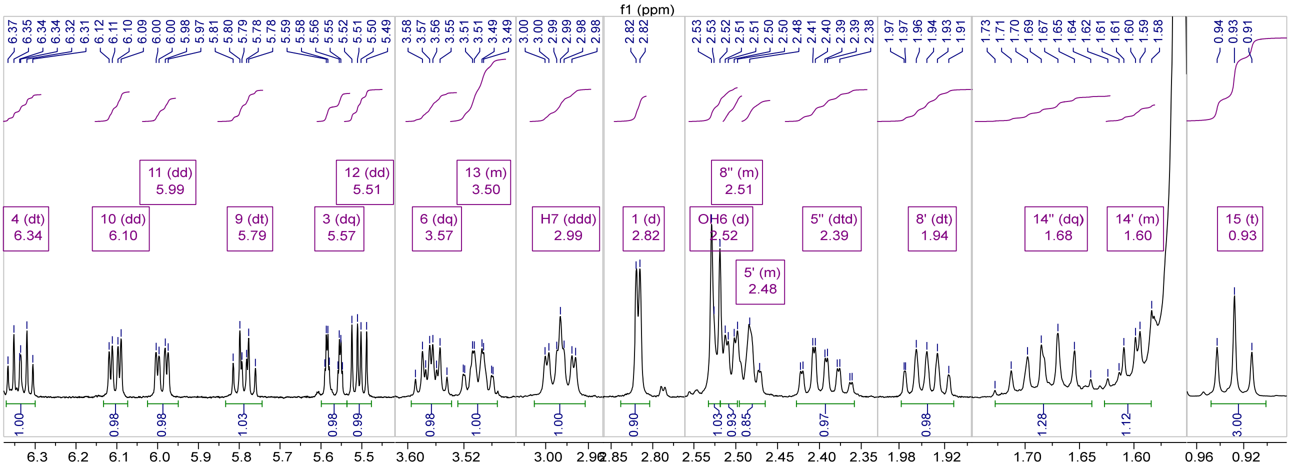

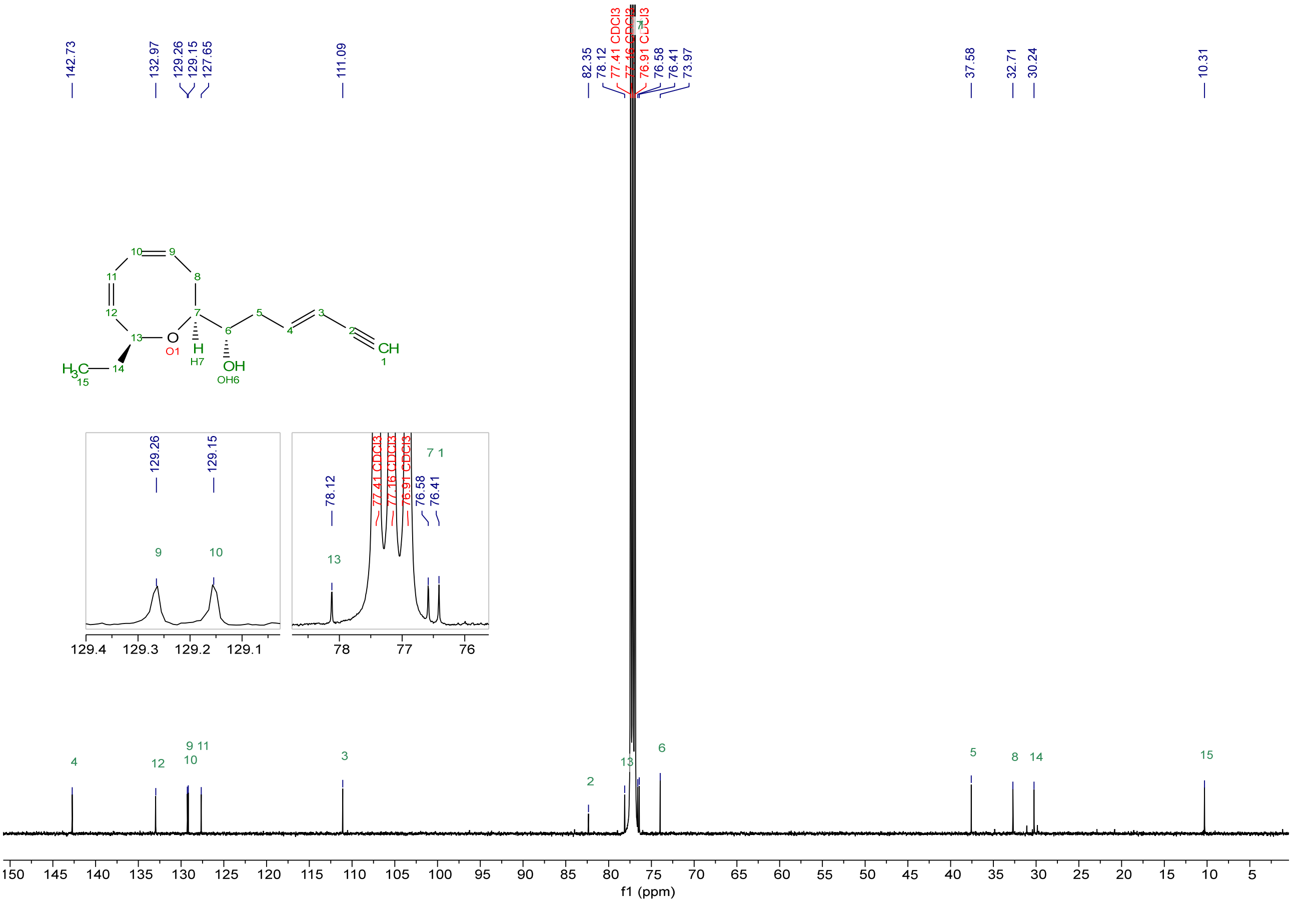

(*S*,*E*)-1-((2*S*,7*R*,8*S*,*Z*)-7-Bromo-8-ethyl-3,6,7,8-tetrahydro-2*H*-oxocin-2-yl)hex-3-en-5-yn-1-ol *ent*-deacetyllaurencin 23 (500MHz, $CDCl_3$)

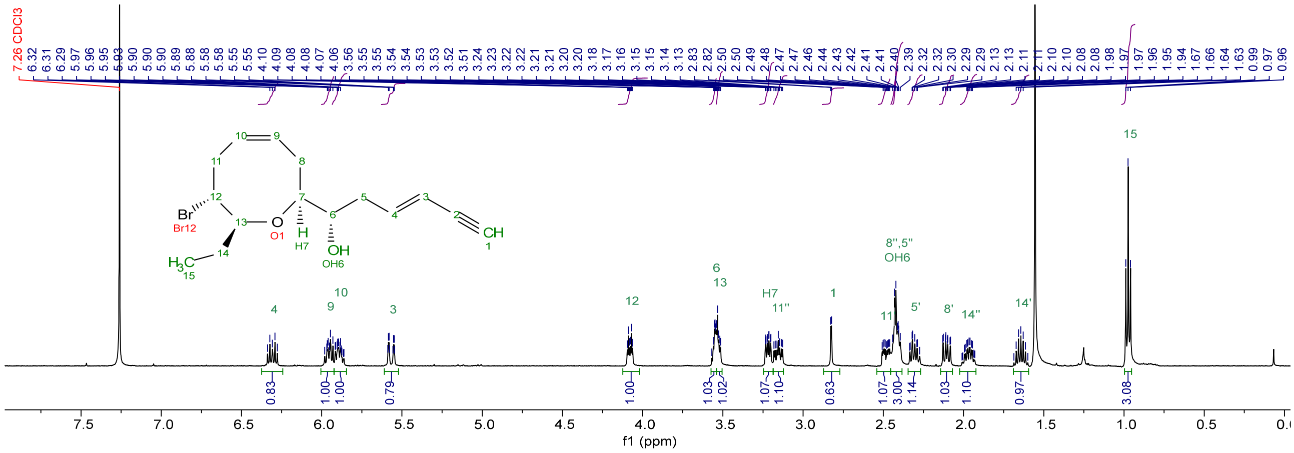

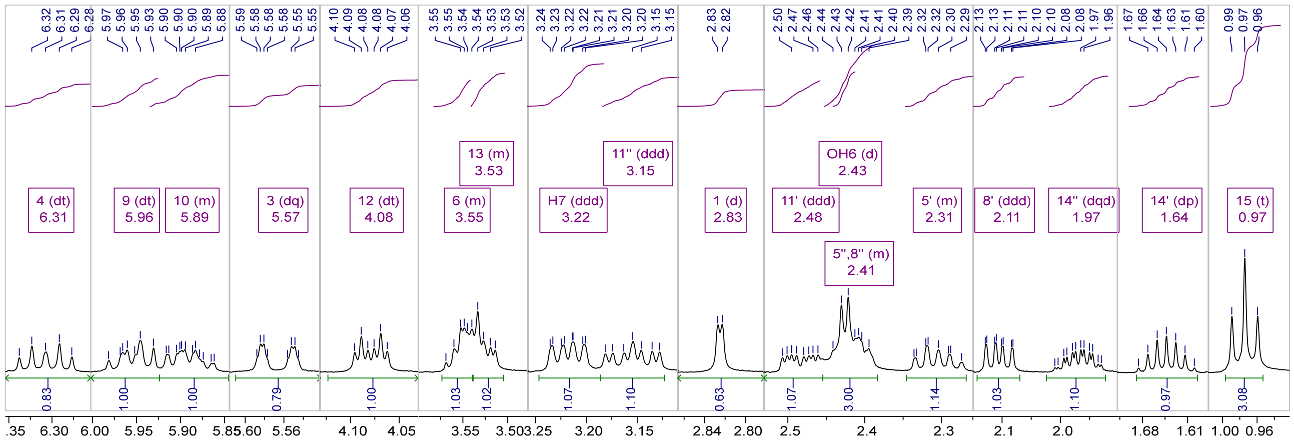

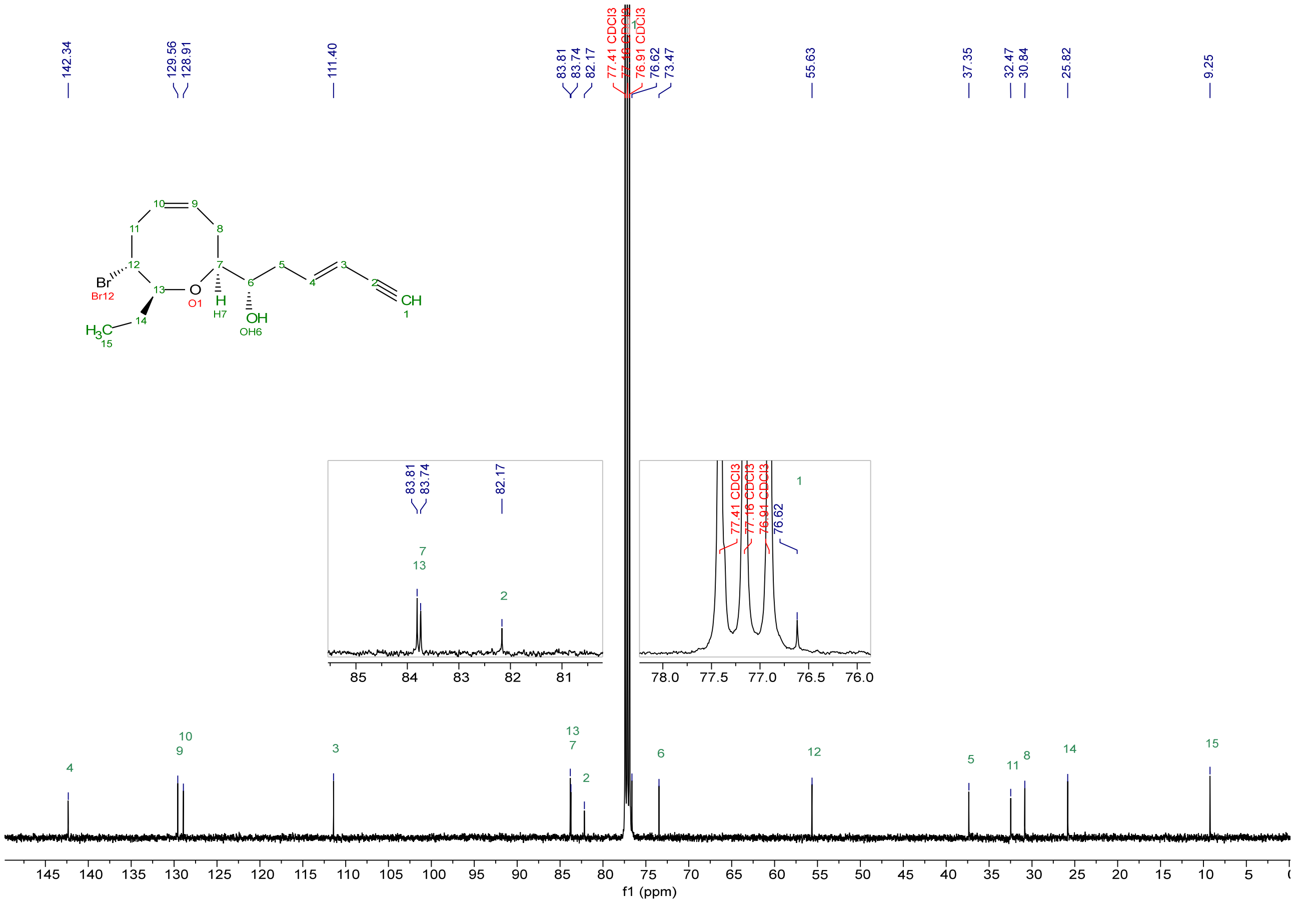

(*S*,*E*)-1-((2*S*,7*R*,8*S*,*Z*)-7-Bromo-8-ethyl-3,6,7,8-tetrahydro-2*H*-oxocin-2-yl)hex-3-en-5-yn-1-ol *ent*-deacetyllaurencin 23 (500MHz, C_6D_6)

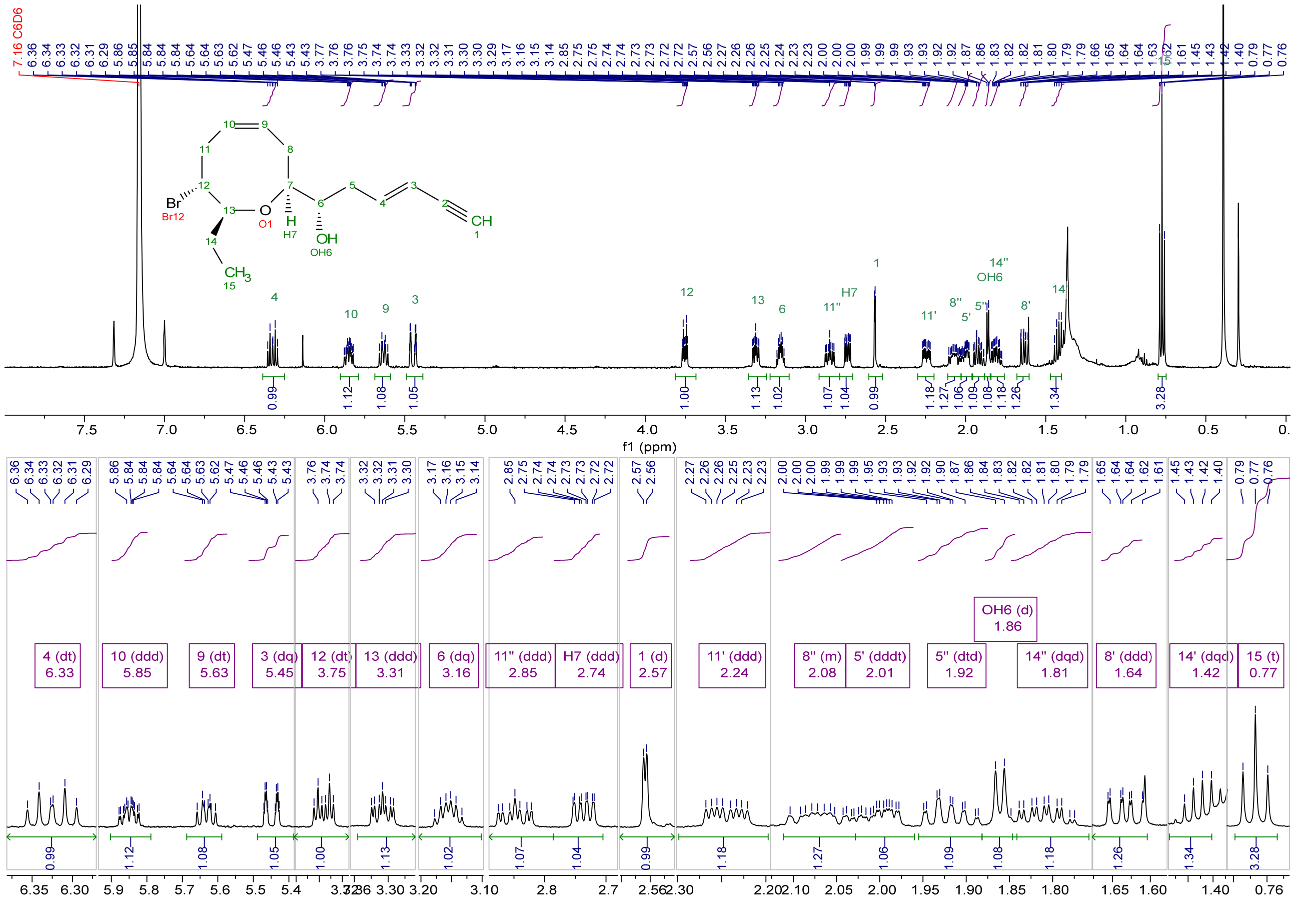

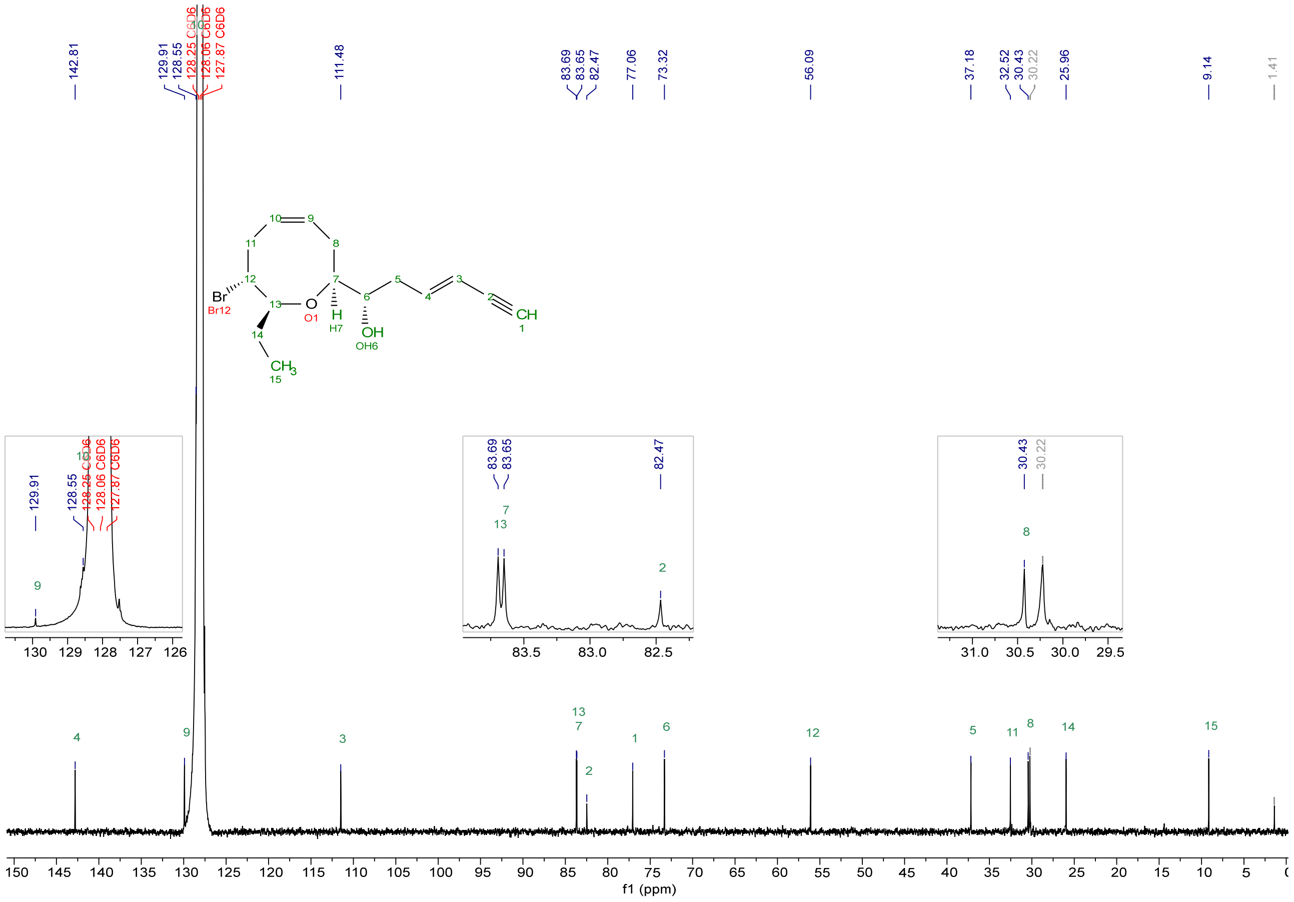

(*S*,*E*)-1-((2*S*,7*R*,8*S*,*Z*)-7-Bromo-8-ethyl-3,6,7,8-tetrahydro-2*H*-oxocin-2-yl)hex-3-en-5-yn-1-yl acetate *ent*-laurencin 24 (500MHz, $CDCl_3$)

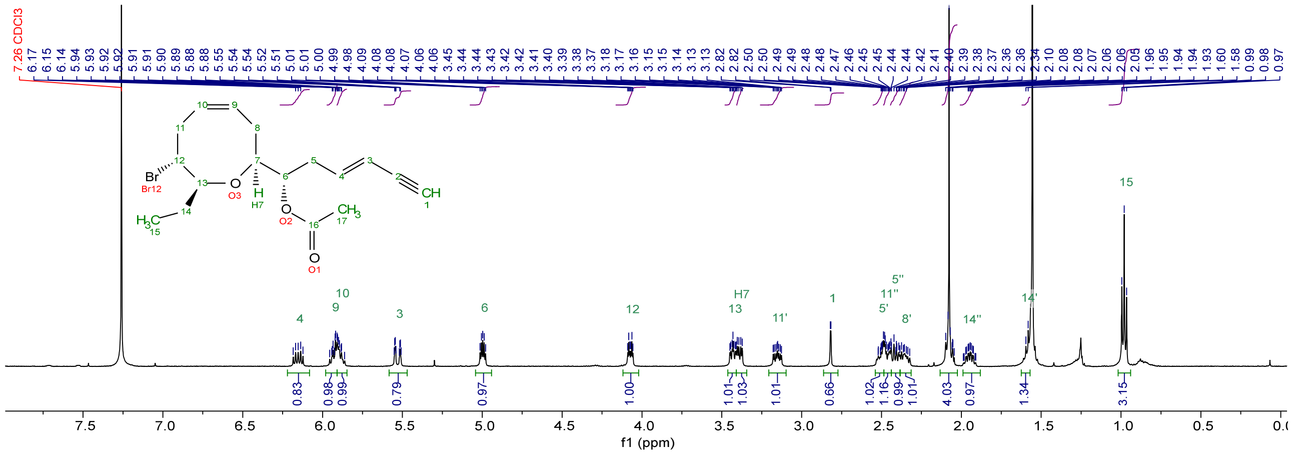

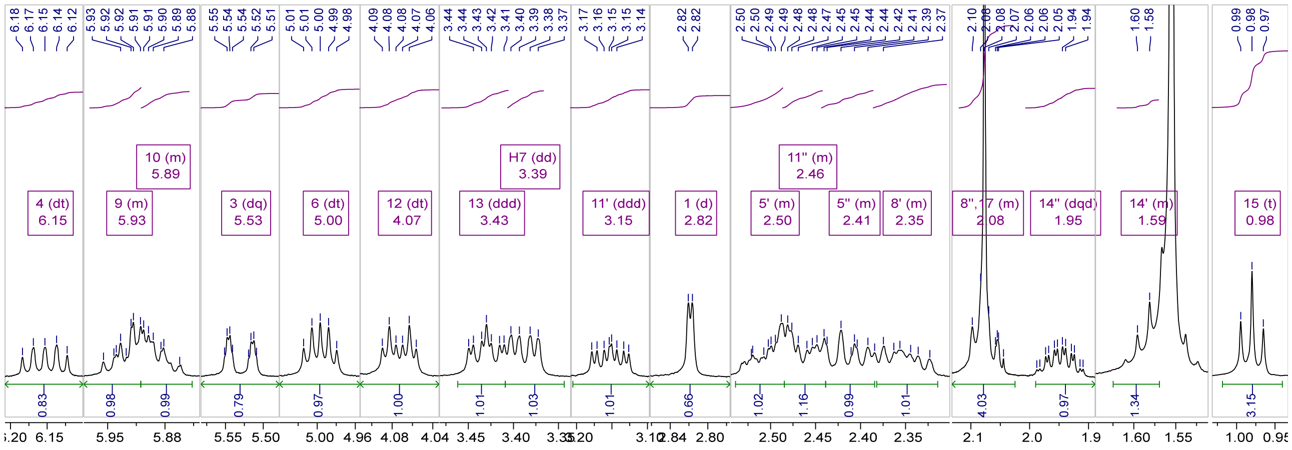

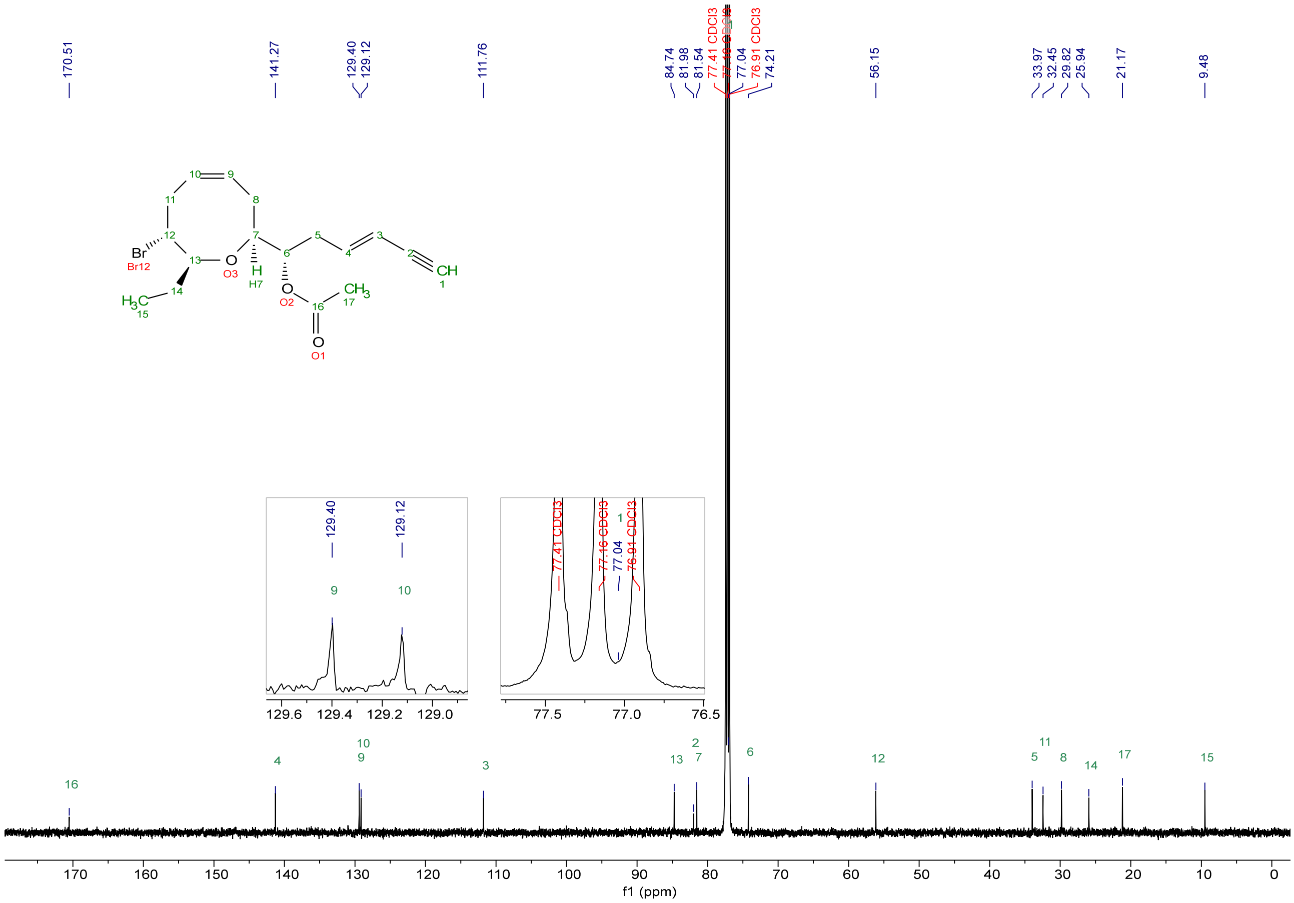

N'-((1S,3S,4R,6S,7S,9S)-9-allyl-4-bromo-3-ethyl-2,8-dioxabicyclo[5.2.1]decan-6-yl)-4-methylbenzenesulfonohydrazide S6 (500MHz, $CDCl_3$)

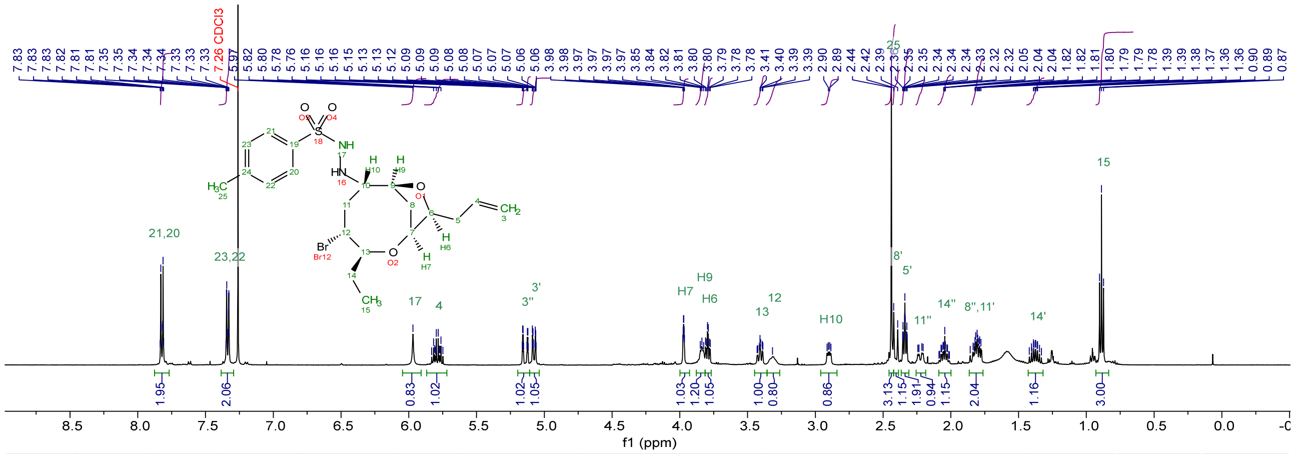

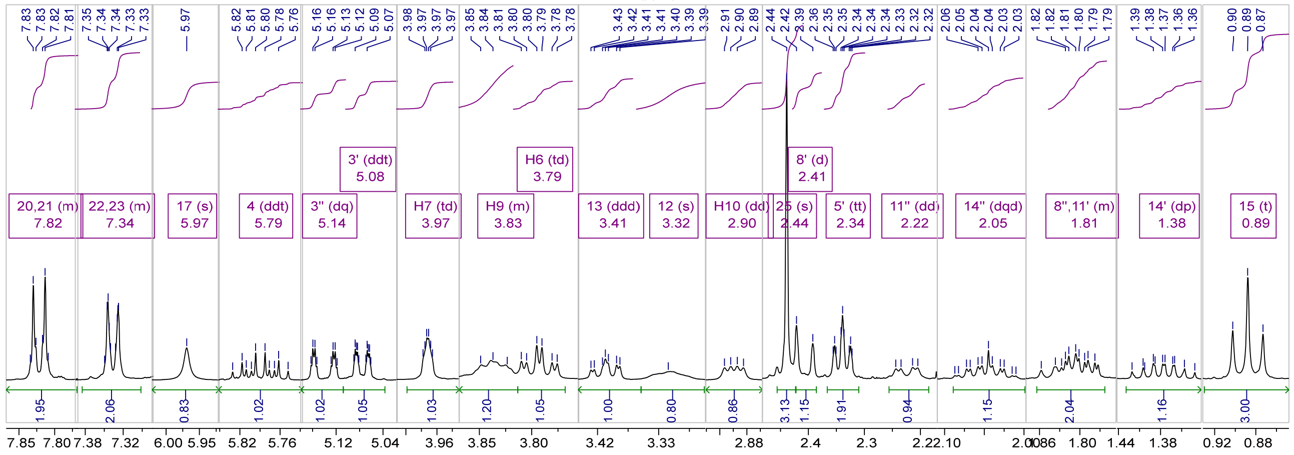

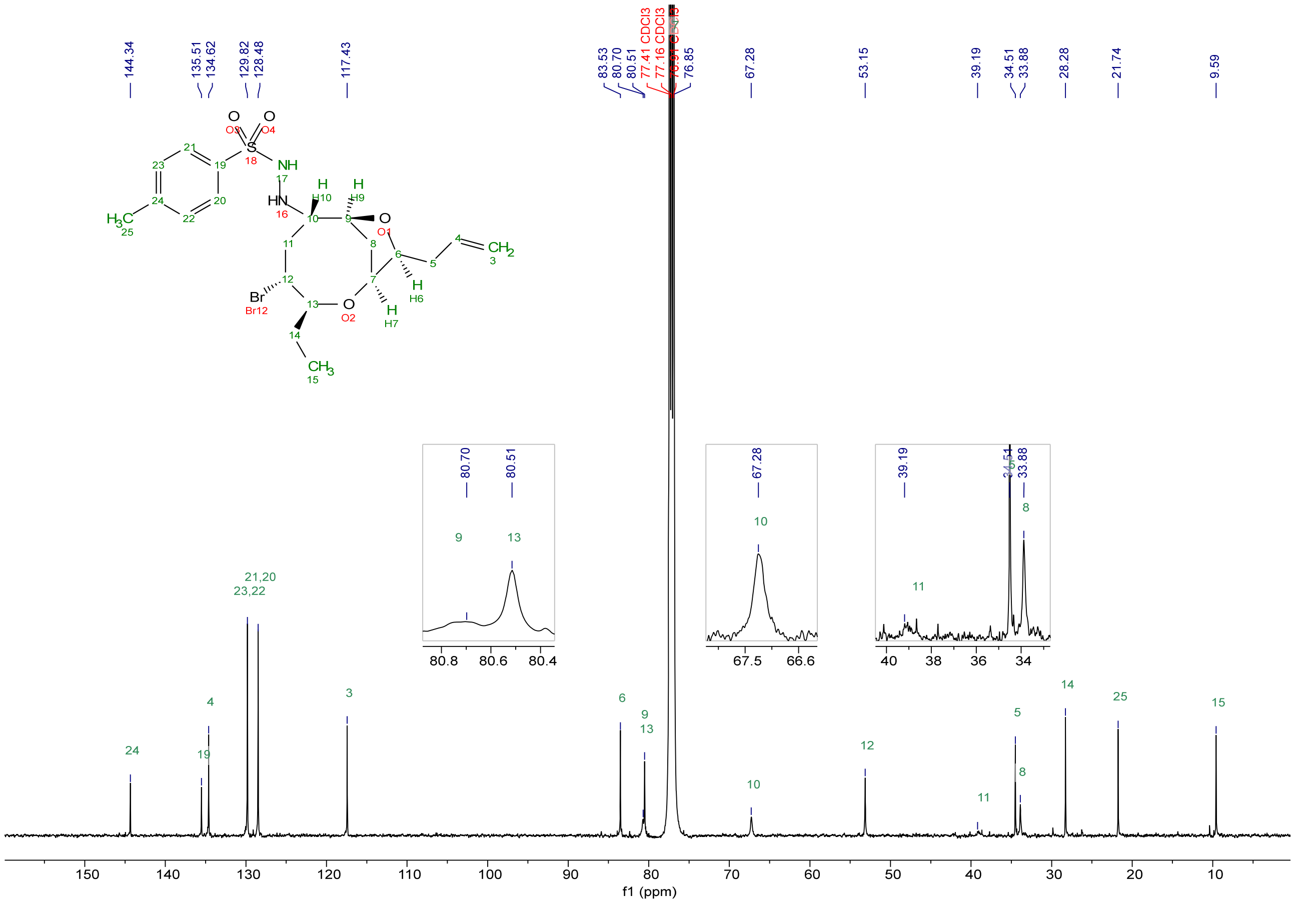

8) References

(1) Chan, H. S. S.; Nguyen, Q. N. N.; Paton, R. S.; Burton, J. W. *J. Am. Chem. Soc.* **2019**, *141*, 15951–15962.

(2) Kim, B.; Lee, M.; Mi, J. K.; Lee, H.; Kim, S.; Kim, D.; Koh, M.; Seung, B. P.; Kye, J. S. *J. Am. Chem. Soc.* **2008**, *130*, 16807–16811.

(3) Snyder, S. A.; Brucks, A. P.; Treitler, D. S.; Moga, I. *J. Am. Chem. Soc.* **2012**, *134*, 17714–17721.

(4) Fukuzawa, A.; Kurosawa, E.; Irie, T. *Tetrahedron Lett.* **1972**, *13*, 3–6.

(5) Voigtritter, K.; Ghorai, S.; Lipshutz, B. H. *J. Org. Chem.* **2011**, *76*, 4697–4702.

(6) Abdel-mageed, W. M.; Ebel, R.; Valeriote, F. A.; Jaspars, M. *Tetrahedron* **2010**, *66*, 2855–2862.

(7) Hoye, T. R.; Jeffrey, C. S.; Shao, F. *Nat. Protoc.* **2007**, *2*, 2451–2458.

(8) Shirokane, K.; Wada, T.; Yoritate, M.; Minamikawa, R.; Takayama, N.; Sato, T.; Chida, N. *Angew. Chem. Int. Ed.* **2014**, *53*, 512–516.

(9) Shepherd, D. J.; Broadwith, P. A.; Dyson, B. S.; Paton, R. S.; Burton, J. W. *Chem. Eur. J* **2013**, *19*, 12644–12648.

(10) Irie, T.; Suzuki, M.; Masamune, T. *Tetrahedron* **1968**, *24*, 4193–4205.

(11) König, G. M.; Wright, A. D. *J. Nat. Prod.* **1994**, *57*, 477–485.

(12) Burton, J. W.; Clark, J. S.; Derrer, S.; Stork, T. C.; Bendall, J. G.; Holmes, A. B. *J. Am. Chem. Soc.* **1997**, *119*, 7483–7498.

(13) Robinson, R. A.; Clark, J. S.; Holmes, A. B. *J. Am. Chem. Soc.* **1993**, *115*, 10400–10401.

(14) Bratz, M.; Bullock, W. H.; Overman, L. E.; Takemoto, T. *J. Am. Chem. Soc.* **1995**, *117*, 5958–5966.

(15) Baek, S.; Jo, H.; Kim, H.; Kim, H.; Kim, S.; Kim, D. *Org. Lett.* **2005**, *7*, 75–77.

(16) Tsushima, K.; Murai, A. *Tetrahedron Lett.* **1992**, *33*, 4345–4348.

(17) Crimmins, M. T.; Emmitte, K. A. *Org. Lett.* **1999**, *1*, 2029–2032.

(18) Fujiwara, K.; Yoshimoto, S.; Takizawa, A.; Souma, S. I.; Mishima, H.; Murai, A.; Kawai, H.; Suzuki, T. *Tetrahedron Lett.* **2005**, *46*, 6819–6822.

(19) Murai, A.; Murase, H.; Matsue, H.; Masamune, T. *Tetrahedron Lett.* **1977**, *18*, 2507–2510.

(20) Fujiwara, K. *J. Synth. Org. Chem. Japan* **2007**, *65*, 502–510.

(21) Irie, T.; Suzuki, M.; Masamune, T. *Tetrahedron Lett.* **1965**, *16*, 1091–1099.

(22) Crimmins, M. T.; Choy, A. L. *J. Am. Chem. Soc.* **1999**, *121*, 5653–5660.
